# Supplementary material for: White matter connections of human ventral temporal cortex are organized by cytoarchitecture, eccentricity, and category-selectivity from birth
Source: bioRxiv. 2025 Jan 23:2024.07.29.605705. Originally published 2024 Jul 29. Preprint. [Version 2] doi: 10.1101/2024.07.29.605705 (PMC11312531; doi:10.1101/2024.07.29.605705)
Supplement: Supplement 1 [file media-1.pdf]

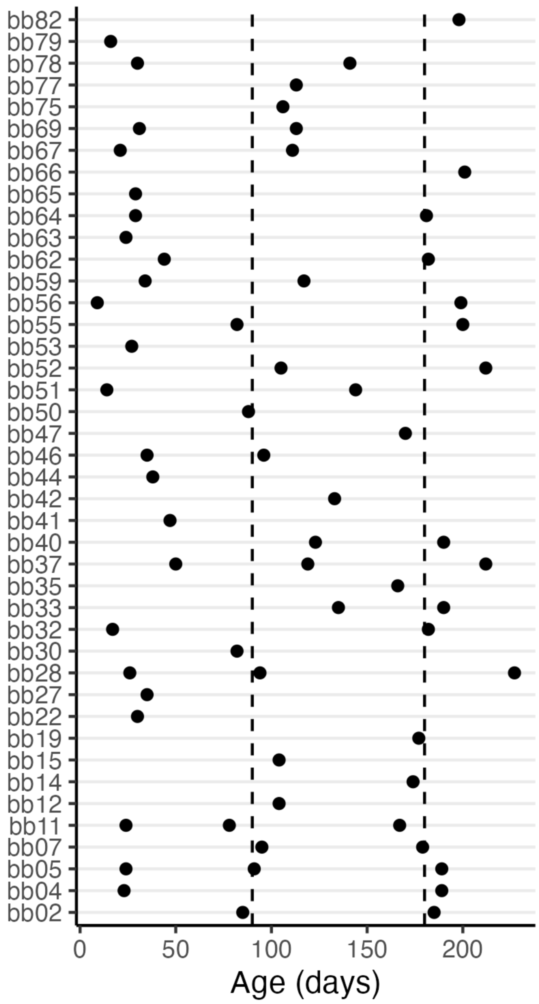

Supplementary Figure 1. Summary of the longitudinal visits for each infant participant. X-axis is the age in days at visit, y-axis is the subject number. Each dot represents a visit. Each infant was scanned one, two, or three times with visits near the three month and six month time points (vertical dashed lines at 90 days (~3 months) and 180 days (~6 months).

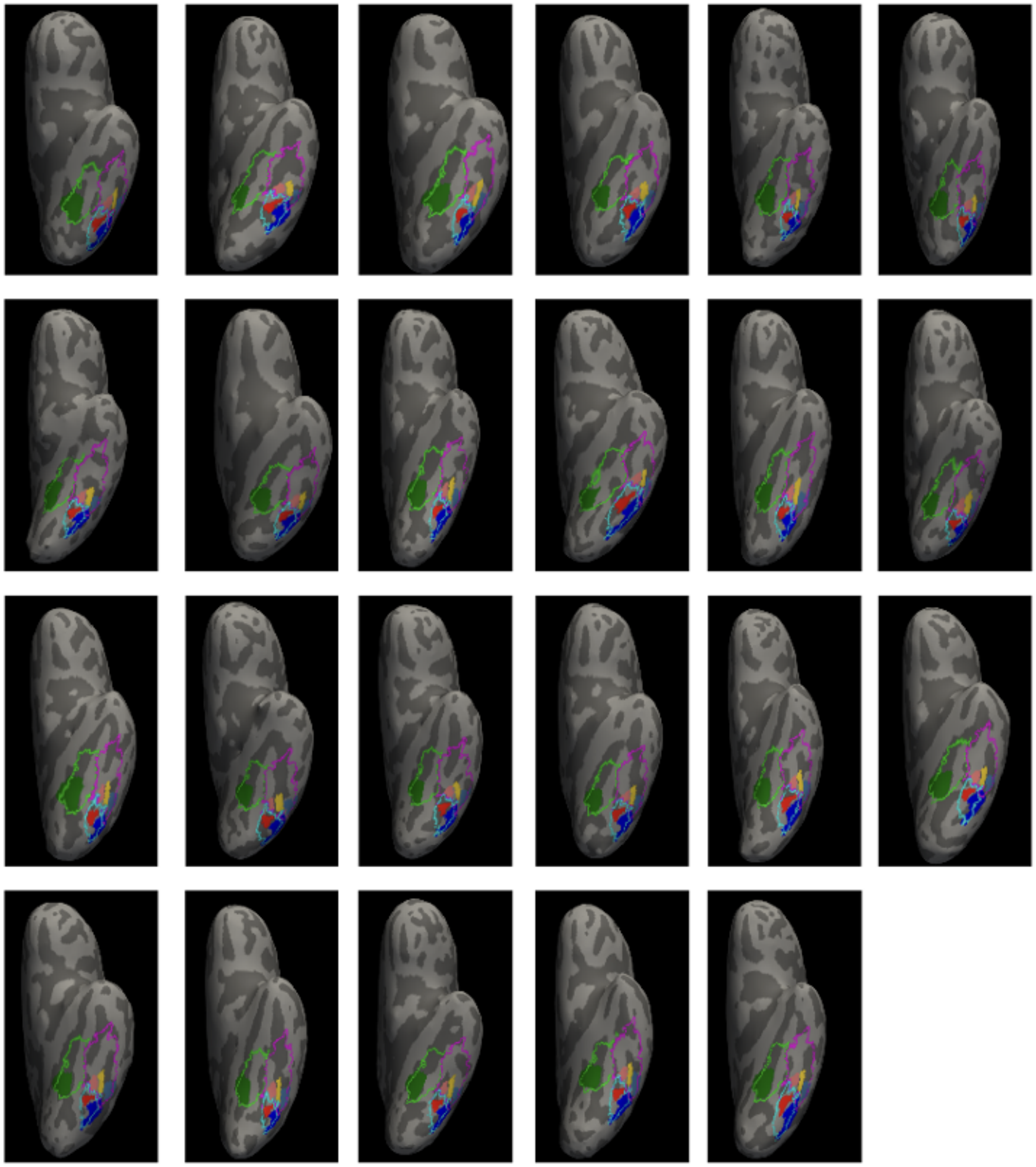

Supplementary Figure 2. Left hemisphere maximal probability map (MPM) of functional regions of interest (fROIs) mapped to the brain of each individual infant subject in the 0-month old age group ordered by age from the upper left to the lower right. Colors indicate functional ROI: *green:* CoS-places; *pink:* mFus-faces, *red:* pFus-faces; *yellow:* OTS-bodies; *light blue:* mOTS-words*; blue:* pOTS-words. Outlines indicate cytoarchitectonic area: *cyan:* FG2, *green:* FG3, *magenta:* FG4.

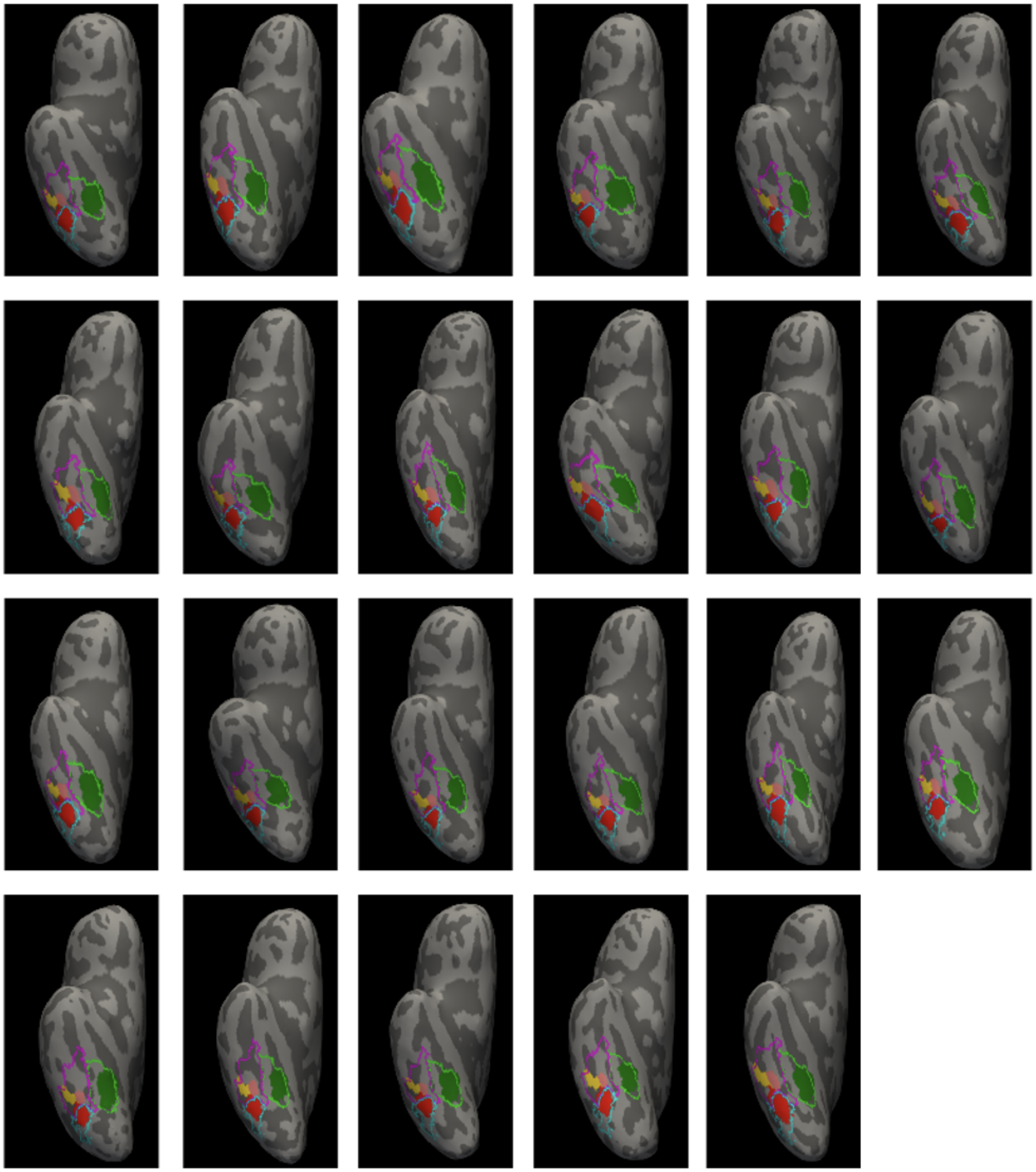

Supplementary Figure 3. Right hemisphere MPM ROIs mapped to the brain of each individual infant subject in the 0-month old age group ordered by age. Colors indicate functional ROI: *green:* CoS-places; *pink:* mFus-faces, *red:* pFus-faces; *yellow:* OTS-bodies; *light blue:* mOTS-words; *blue:* pOTS-words. Outlines indicate cytoarchitectonic area: *cyan:* FG2, *green:* FG3, *magenta:* FG4.

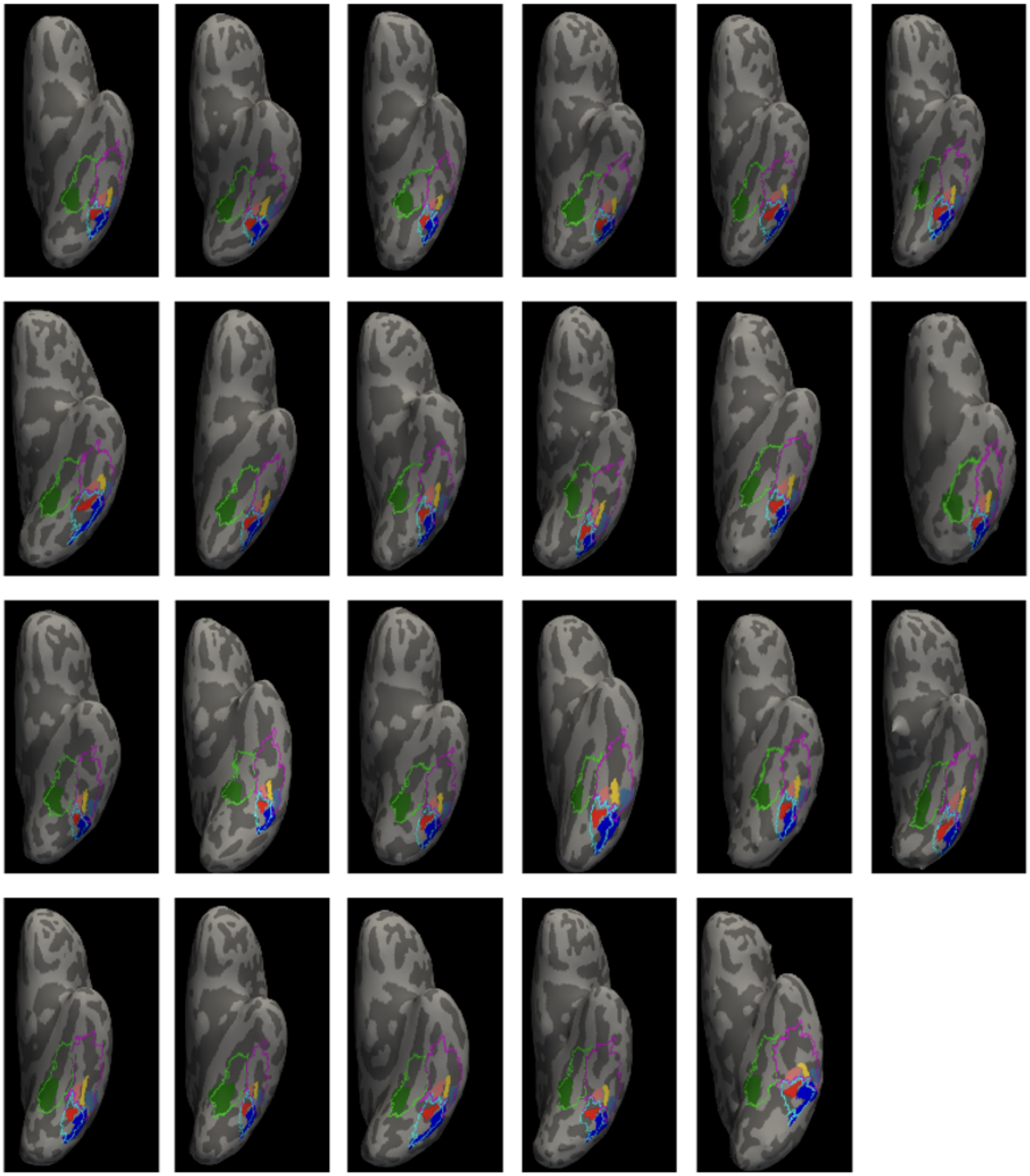

Supplementary Figure 4. Left hemisphere MPM fROIs mapped to the brain of each individual infant subject in the 3-month old age group ordered by age. Colors indicate functional ROI: *green:* CoS-places; *pink:* mFus-faces, *red:* pFus-faces; *yellow:* OTS-bodies; *light blue*: mOTS-words; *blue:* pOTS-words. Outlines indicate cytoarchitectonic area: *cyan:* FG2, *green:* FG3, *magenta:* FG4.

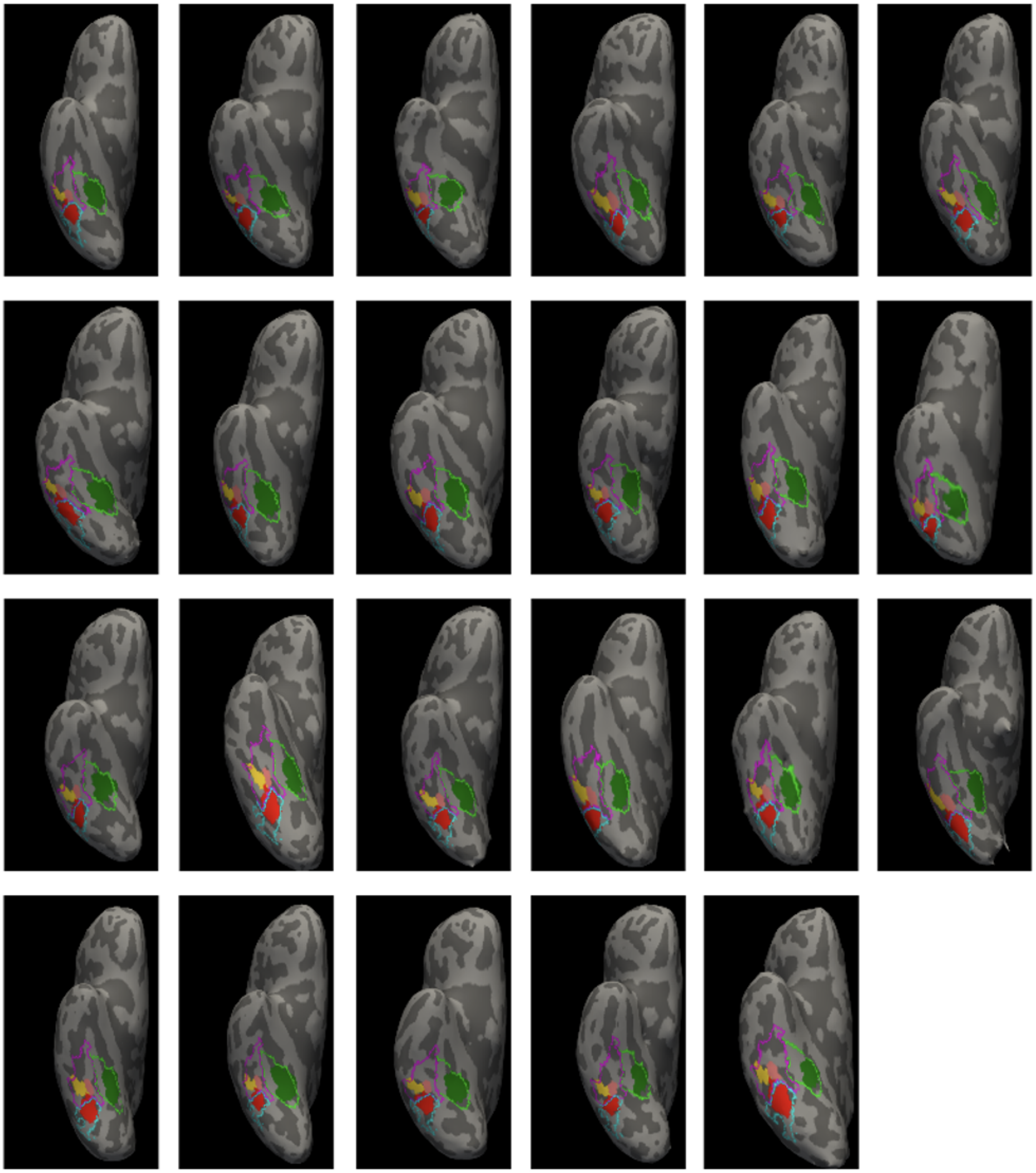

Supplementary Figure 5. Right hemisphere MPM ROIs mapped to the brain of each individual infant subject in the 3-month old age group ordered by age. Colors indicate functional ROI: *green:* CoS-places; *pink:* mFus-faces, *red:* pFus-faces; *yellow:* OTS-bodies; *light blue:* mOTS-words; *blue:* pOTS-words. Outlines indicate cytoarchitectonic area: *cyan:* FG2, *green:* FG3, *magenta:* FG4.

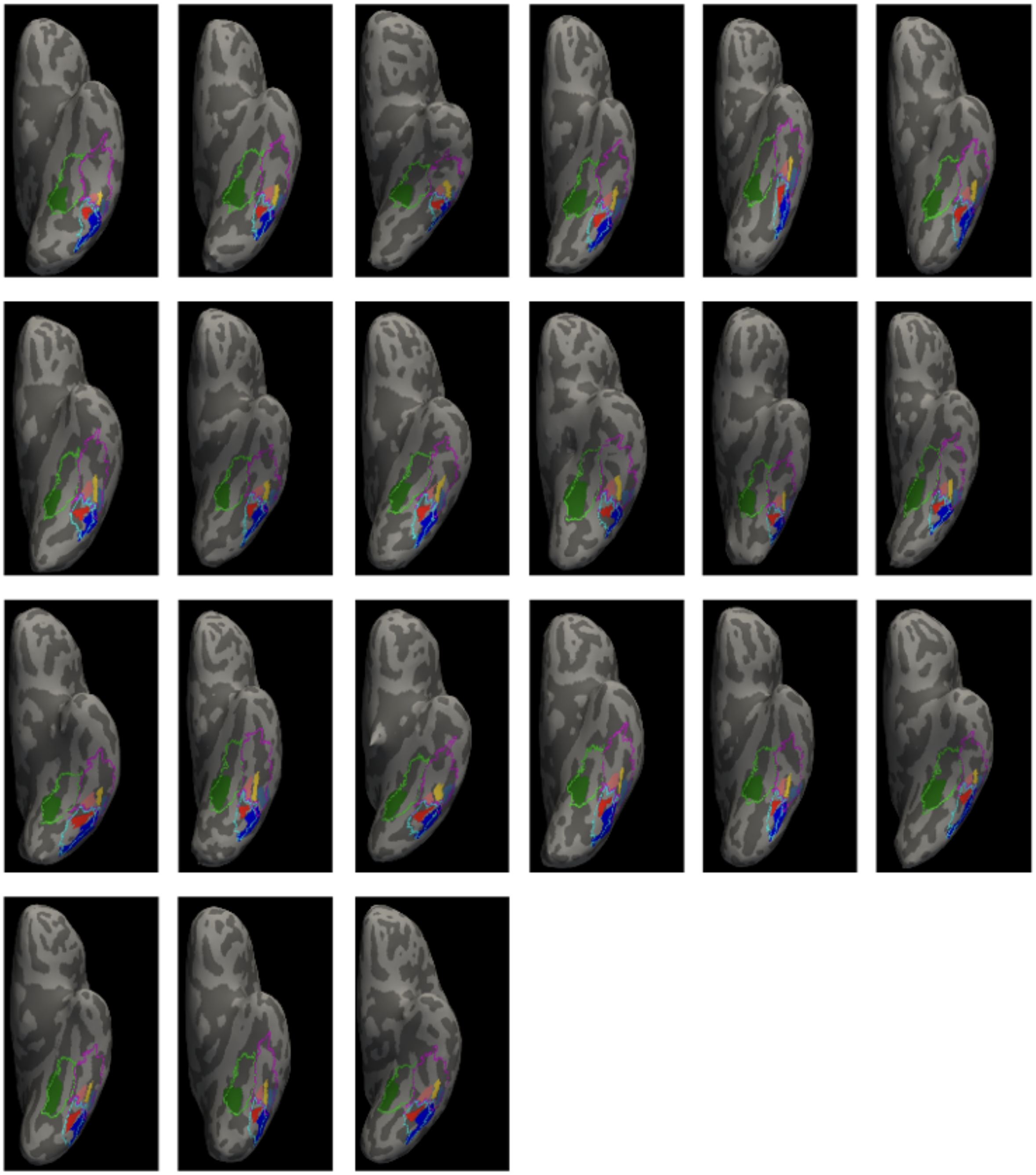

Supplementary Figure 6. Left hemisphere MPM fROIs mapped to the brain of each individual infant subject in the 6-month old age group ordered by age. Colors indicate functional ROI: green: CoS-places; pink: mFus-faces, red: pFus-faces; yellow: OTS-bodies; light blue: mOTS-;words; blue: pOTS-words. Outlines indicate cytoarchitectonic area: *cyan:* FG2, *green:* FG3, *magenta:* FG4.

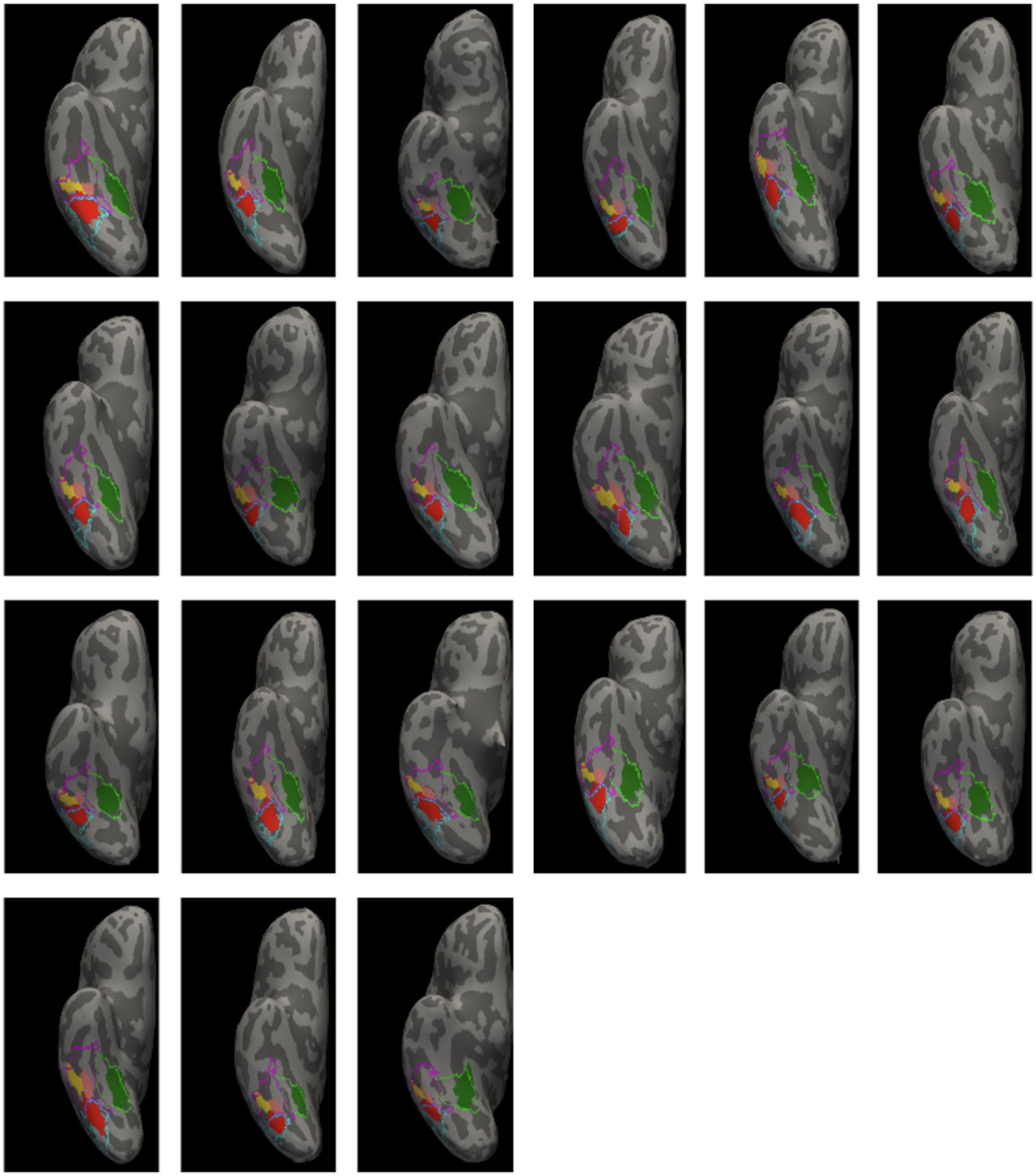

Supplementary Figure 7. Right hemisphere MPM fROIs mapped to the brain of each individual infant subject in the 6-month old age group ordered by age. Colors indicate functional ROI: green: CoS-places; pink: mFus-faces, red: pFus-faces; yellow: OTS-bodies; light blue: mOTS-words; blue: pOTS-words. Outlines indicate cytoarchitectonic area: *cyan:* FG2, *green:* FG3, *magenta:* FG4.

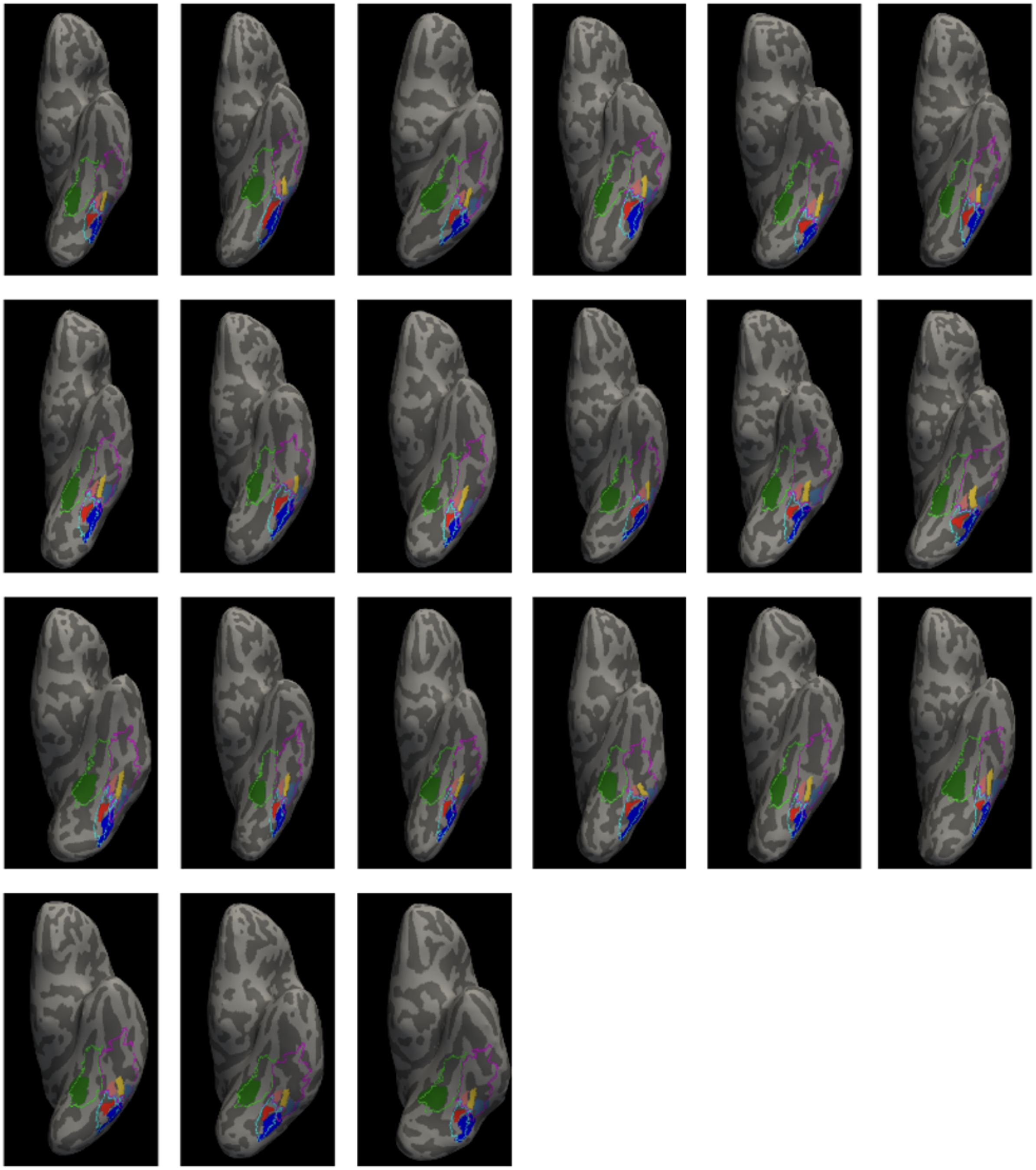

Supplementary Figure 8. Left hemisphere MPM ROIs mapped to the brain of each individual subject in the adult age group ordered by age. Colors indicate functional ROI: green: CoS-places; pink: mFus-faces, red: pFus-faces; yellow: OTS-bodies; light blue: mOTS-words; blue: pOTS-words. Outlines indicate cytoarchitectonic area: *cyan:* FG2, *green:* FG3, *magenta:* FG4.

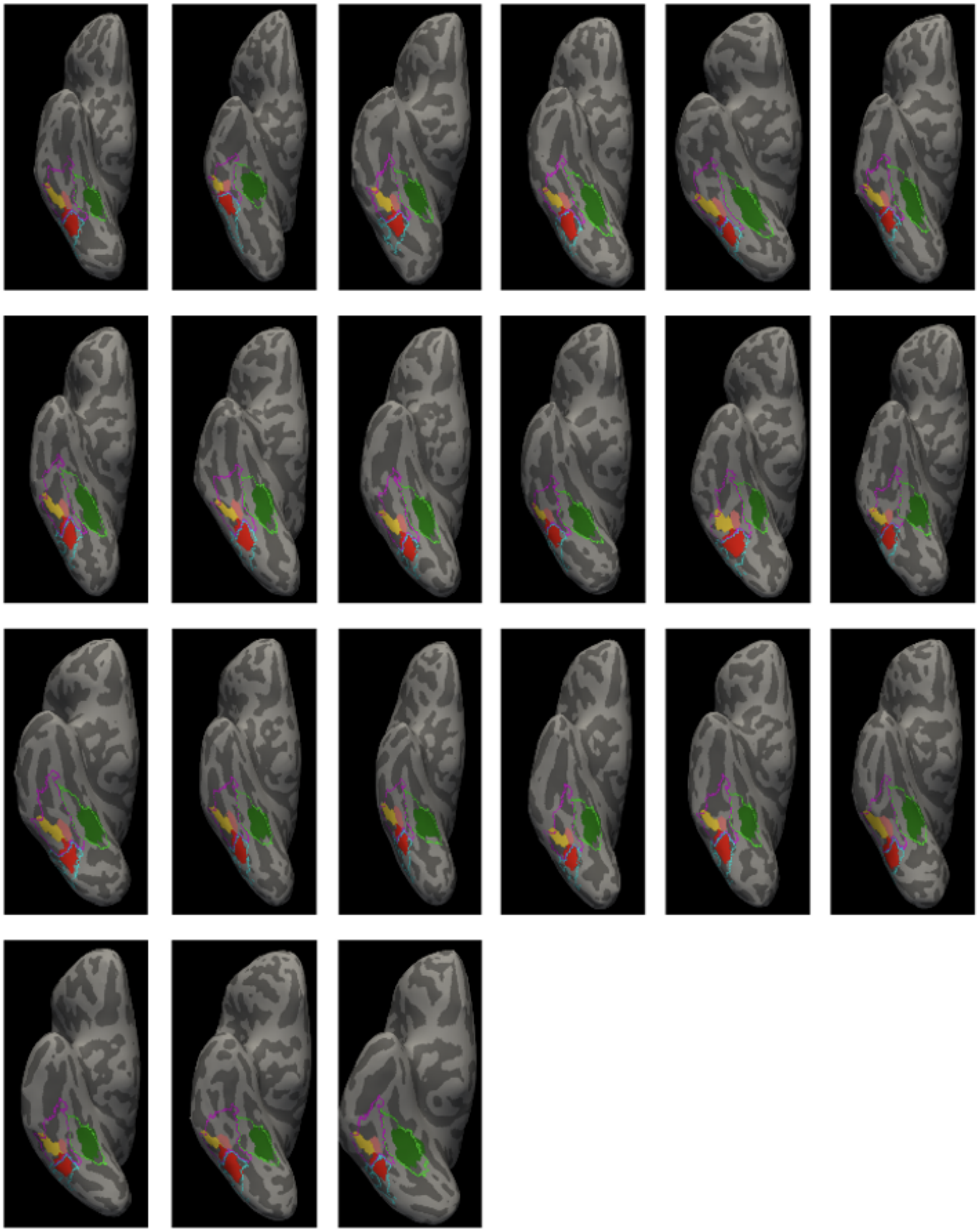

Supplementary Figure 9. Right hemisphere MPM fROIs mapped to the brain of each individual infant subject in the adult old age group ordered by age. Colors indicate functional ROI: green: CoS-places; pink: mFus-faces, red: pFus-faces; yellow: OTS-bodies; light blue: mOTS-words; blue: pOTS-words. Outlines indicate cytoarchitectonic area: *cyan:* FG2, *green:* FG3, *magenta:* FG4.

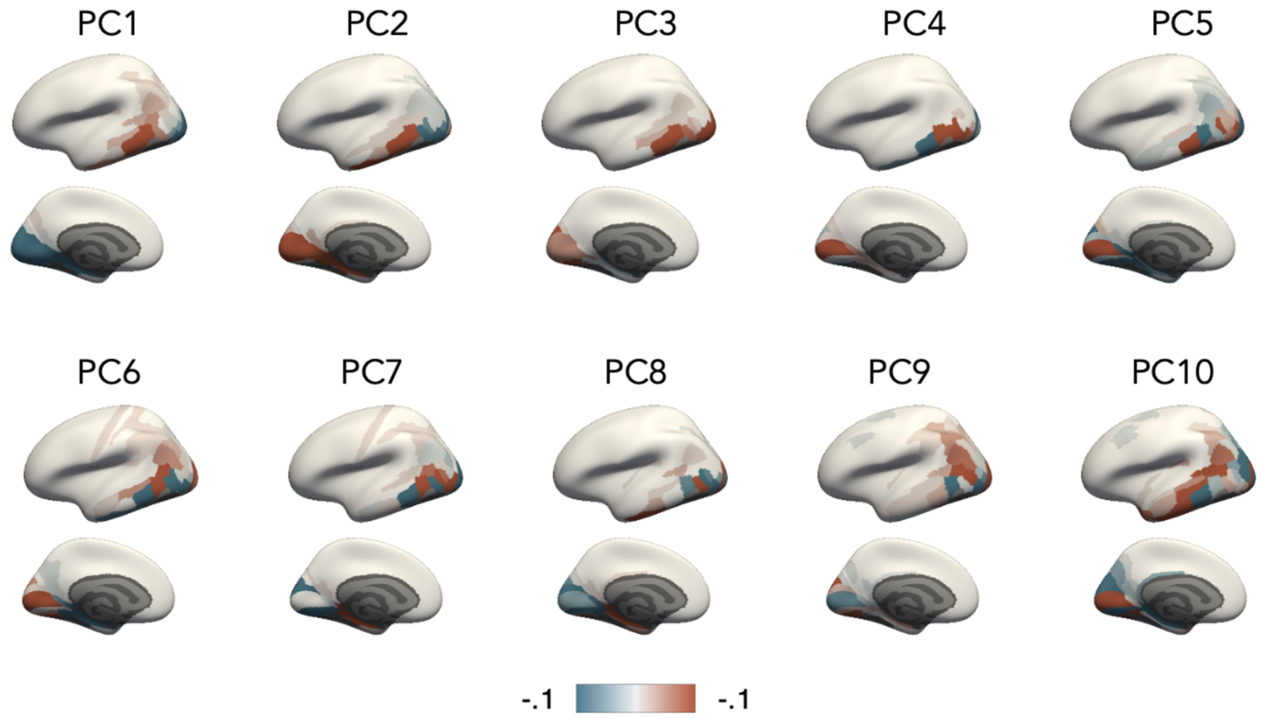

Supplementary Figure 10. Loadings of each of the 10 first principal components (PCs) across Glasser ROIs. Colors indicate the coefficient (see color bar).

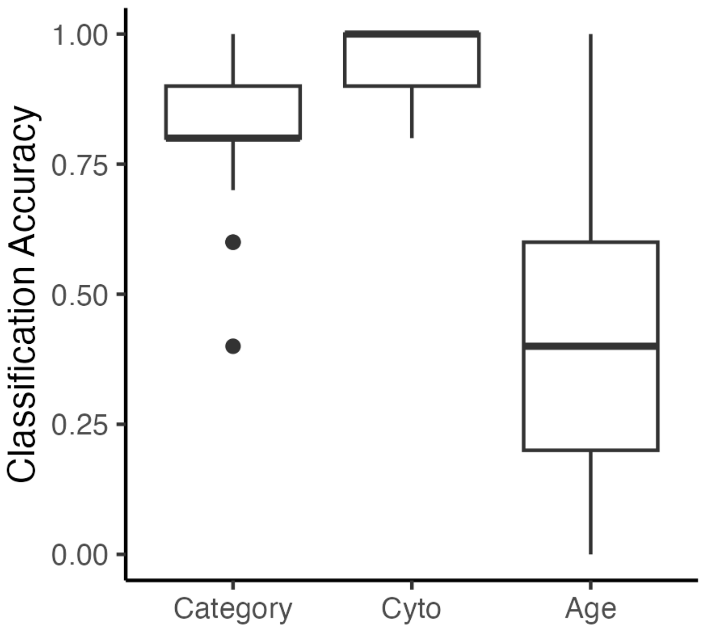

Supplementary Figure 11. Boxplots depicting distribution of average leave-one-out classification accuracy of category, cytoarchitecture (Cyto), and age from white matter connectivity profiles (n=88). Center line indicates median, whiskers depict upper and lower quartiles, and points indicate outliers.

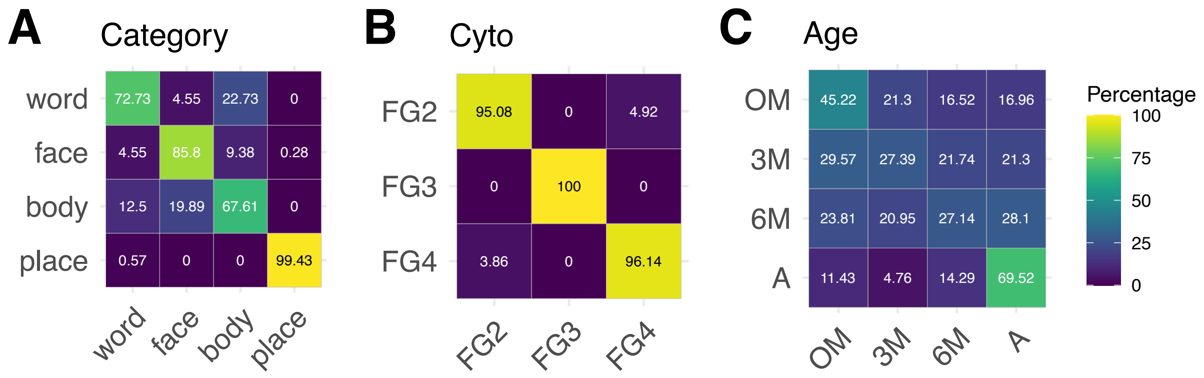

Supplementary Figure 12. Confusion matrices for classification of the white matter connectivity profiles of VTC fROIs (n=10) in each participant (n=88) using a balanced training set with 200 examples per each label (sampled with replacement). A) Category classification. B) Cytoarchitecture classification. C) Age classification. All panels: Rows sum to 100. Color depicts the percentage of samples classified within each bin; brighter colors indicate higher percentage of samples (see color bar). On diagonal values are % correct classification and off diagonal values are % incorrect classification.

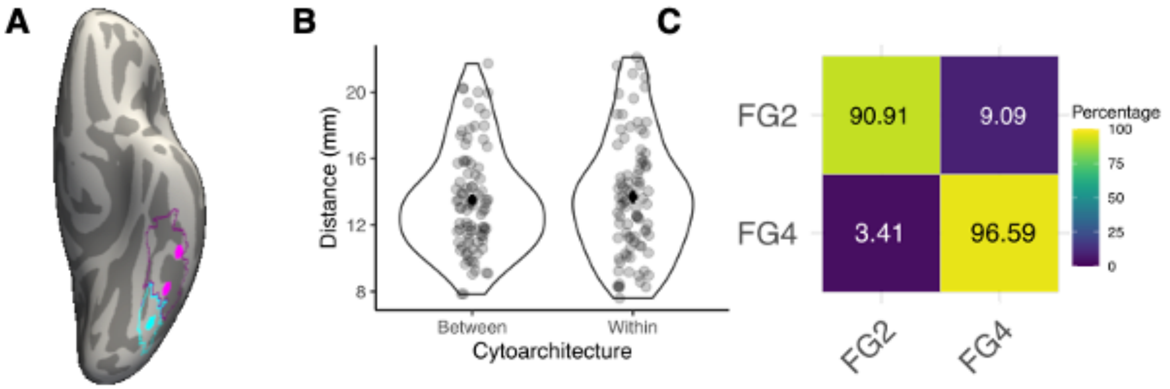

Supplementary Figure 13. When holding distance between fROIs constant, cytoarchitecture can be successfully classified from connectivity profiles. A) Disks placed on the cortical surface such that the middle disk is equidistant from two disks, one within the same cytoarchitectonic area (FG4; magenta) and the other in a different cytoarchitectonic area (FG2; cyan). B) Violin plots depicting distance between the ROIs within the same cytoarchitectonic area or between cytoarchitectonic areas, showing no difference in distance ($t(87) = .49$, $p = 0.62$); Black dot and error bar indicate mean± standard error of the mean; transparent dots (n = 88) indicate individual participants C) Confusion matrices depicting leave-one-out cross validation classification accuracy of cytoarchitecture classification in each participant (n=88) in each control ROI (n=3). Rows sum to 100. Color depicts the percentage of samples classified within each bin; brighter colors indicate higher percentage of samples (see color bar). On diagonal values are percentage correct classification and off diagonal values are percentage incorrect classification. Cytoarchitecture is classified successfully and significantly above chance (mean accuracy ± SD=95% ± 13%, $t(87)= 31.62$, $p = 2.2\times10^{-16}$).

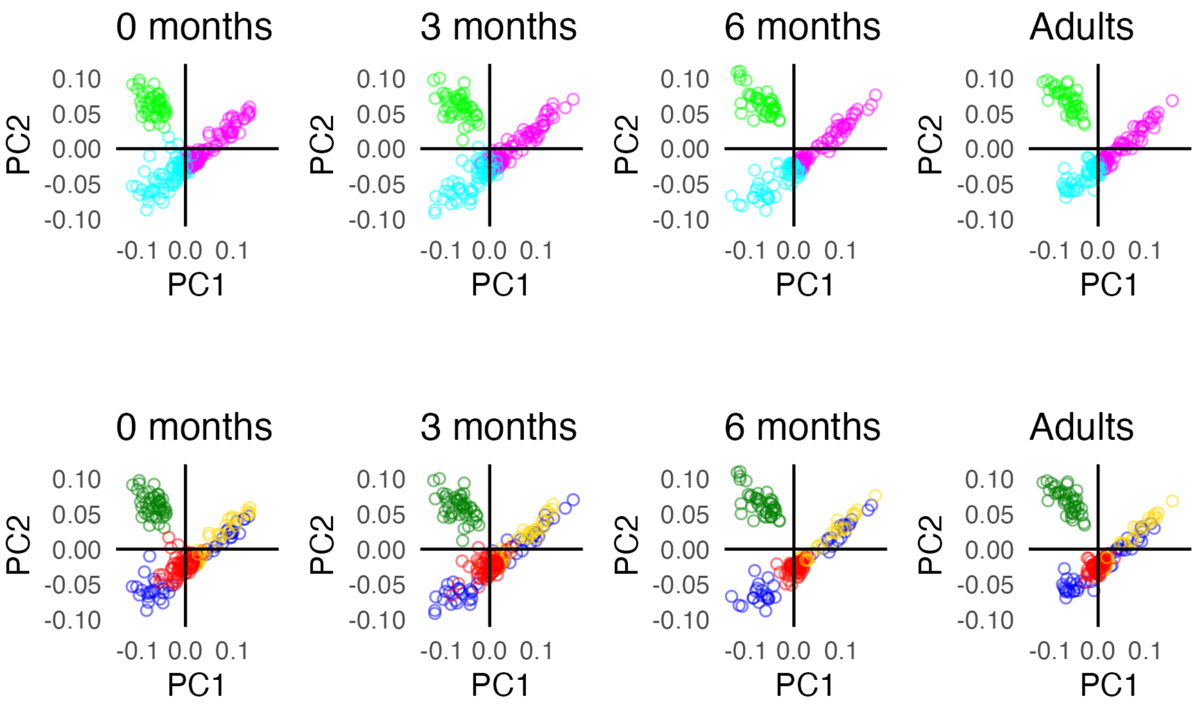

Supplementary Figure 14. White matter connectivity profiles of all participants and fROIs projected to the first two principal components (PC1 and PC2). Each dot is a connectivity profile. Each column is an age group. Top row: colored by cytoarchitectonic area: *cyan:* FG2, *green:* FG3, *magenta:* FG4. Bottom row: colored by category: *red*: faces, *green:* places, *blue*: words, *yellow:* bodies. Mean classification accuracy±SD for cytoarchitecture : 0 months: 97%± 16%, 3-months: 97%± 18%, 6-months: 96%±20%, adults: 97%±18%. Mean classification accuracy±SD for category: 0 months: 84%± 37%, 3-months: 82%± 39%, 6-months: 83%±37%, adults: 81%±39%.

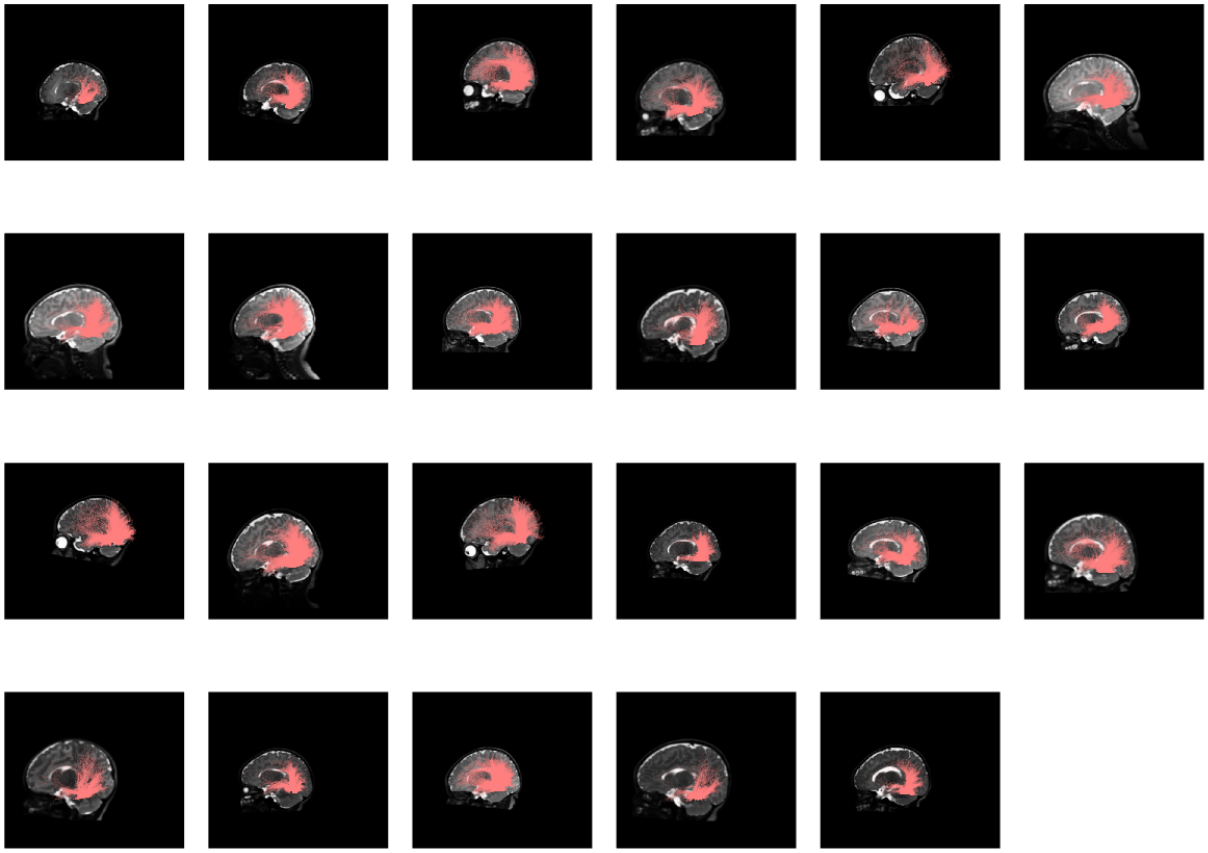

Supplementary Figure 15. White matter connections of mFus-faces in the 0-month age group in the left hemisphere. Each panel is a different individual and session. Panels are organized by age from top left to bottom right, with the youngest in the top left.

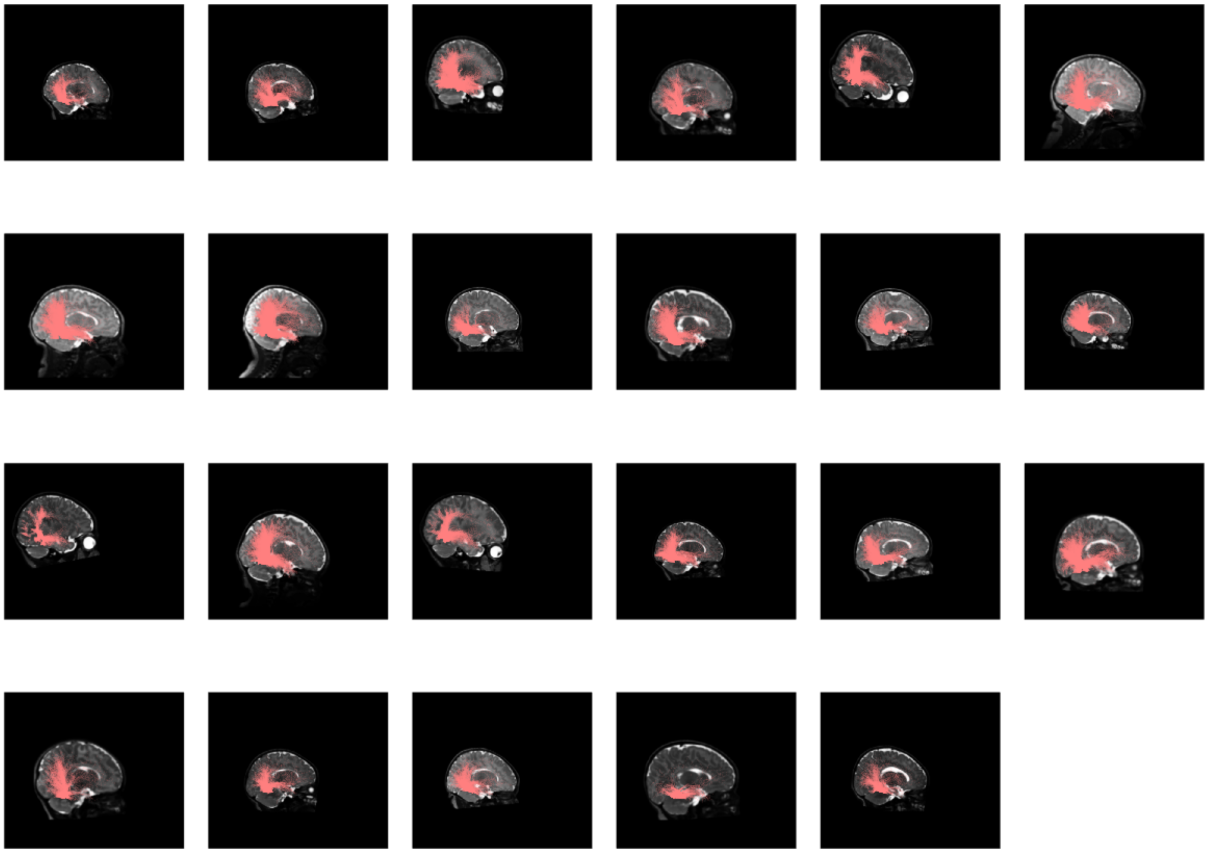

Supplementary Figure 16. White matter connections of mFus-faces in the 0-month age group organized by age in the right hemisphere.

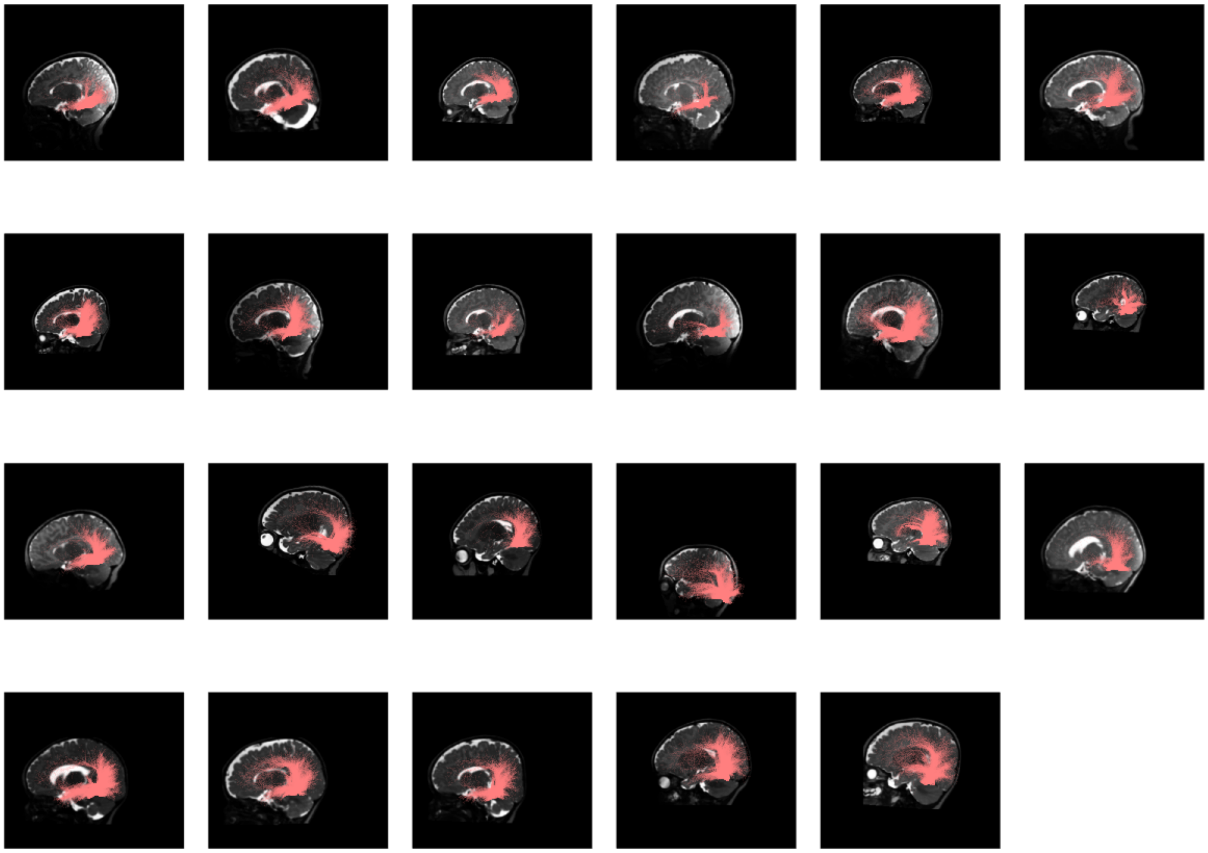

Supplementary Figure 17. White matter connections of mFus-faces in the 3-month age group organized by age in the left hemisphere.

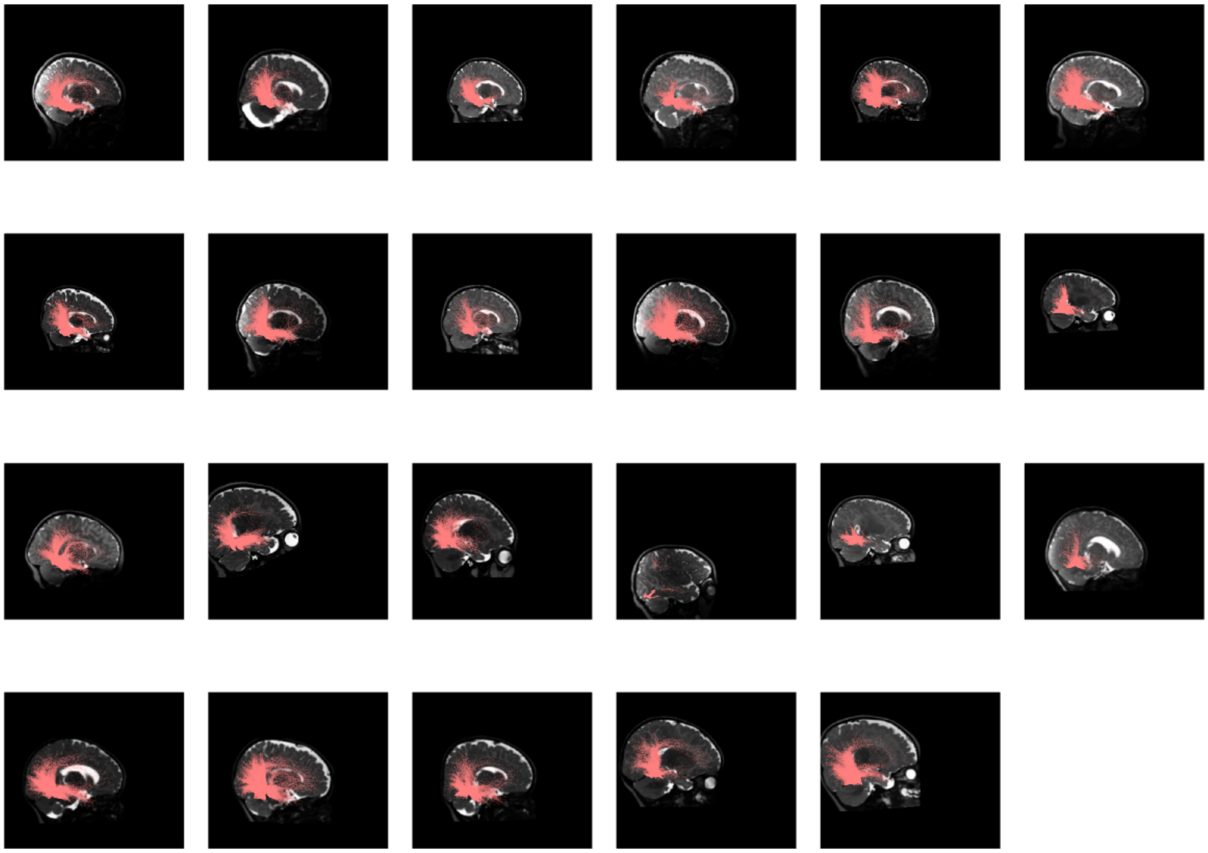

Supplementary Figure 18. White matter connections of mFus-faces in the 3-month age group organized by age in the right hemisphere.

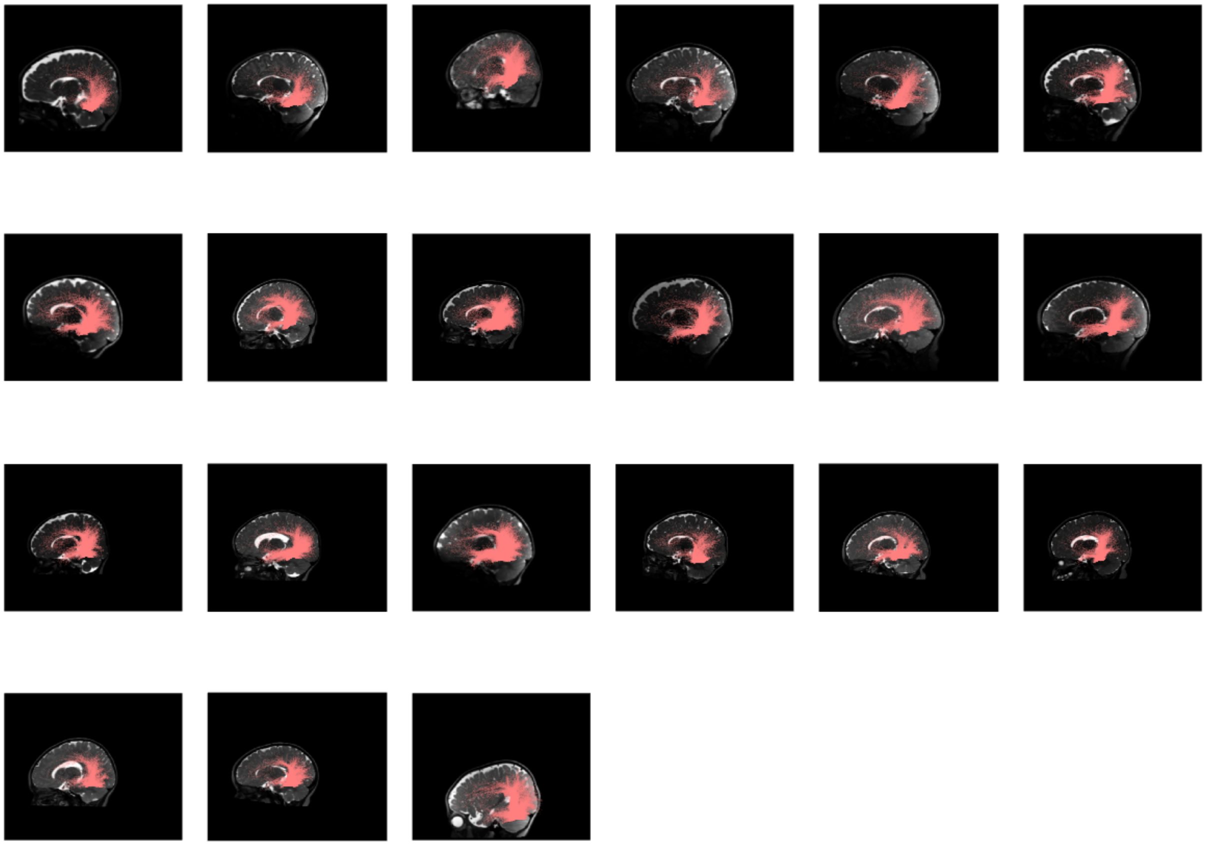

Supplementary Figure 19. White matter connections of mFus-faces in the 6-month age group organized by age in the left hemisphere.

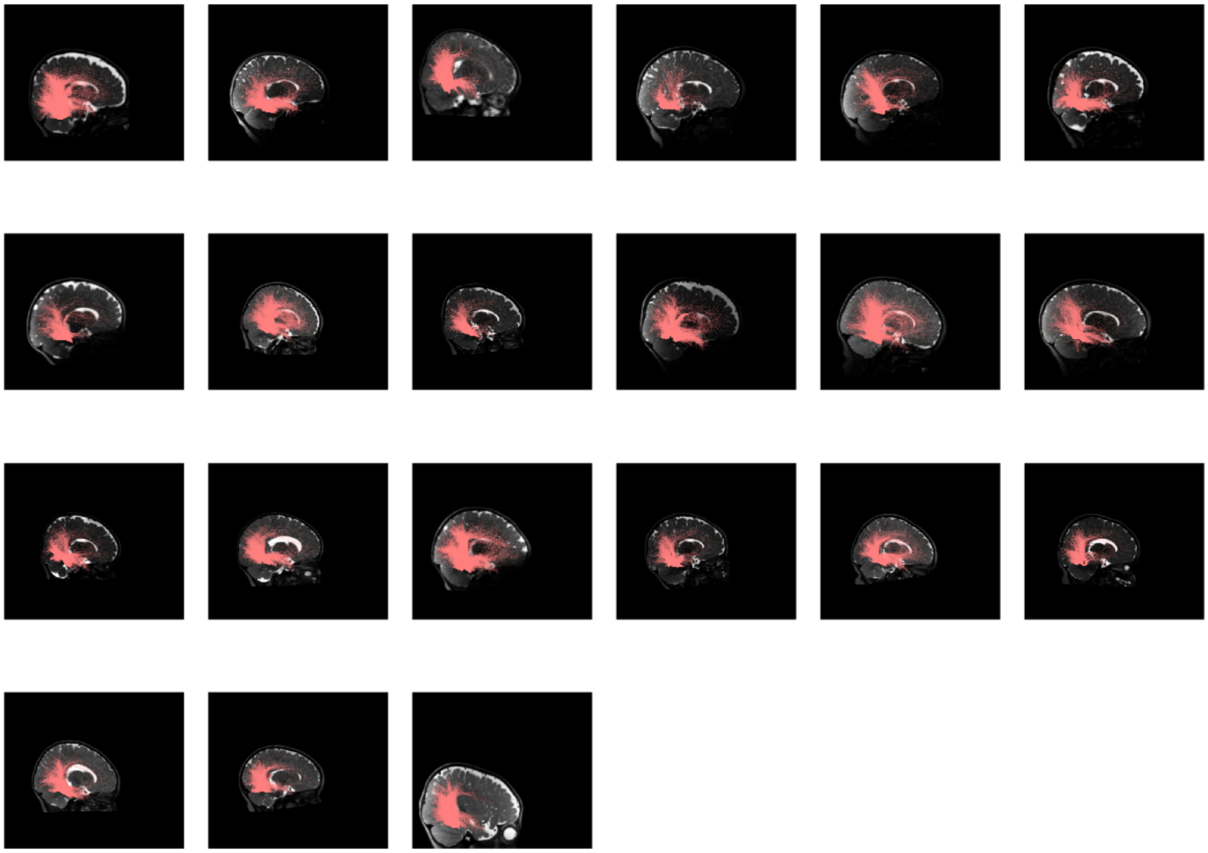

Supplementary Figure 20. White matter connections of mFus-faces in the 6-month age group organized by age in the right hemisphere.

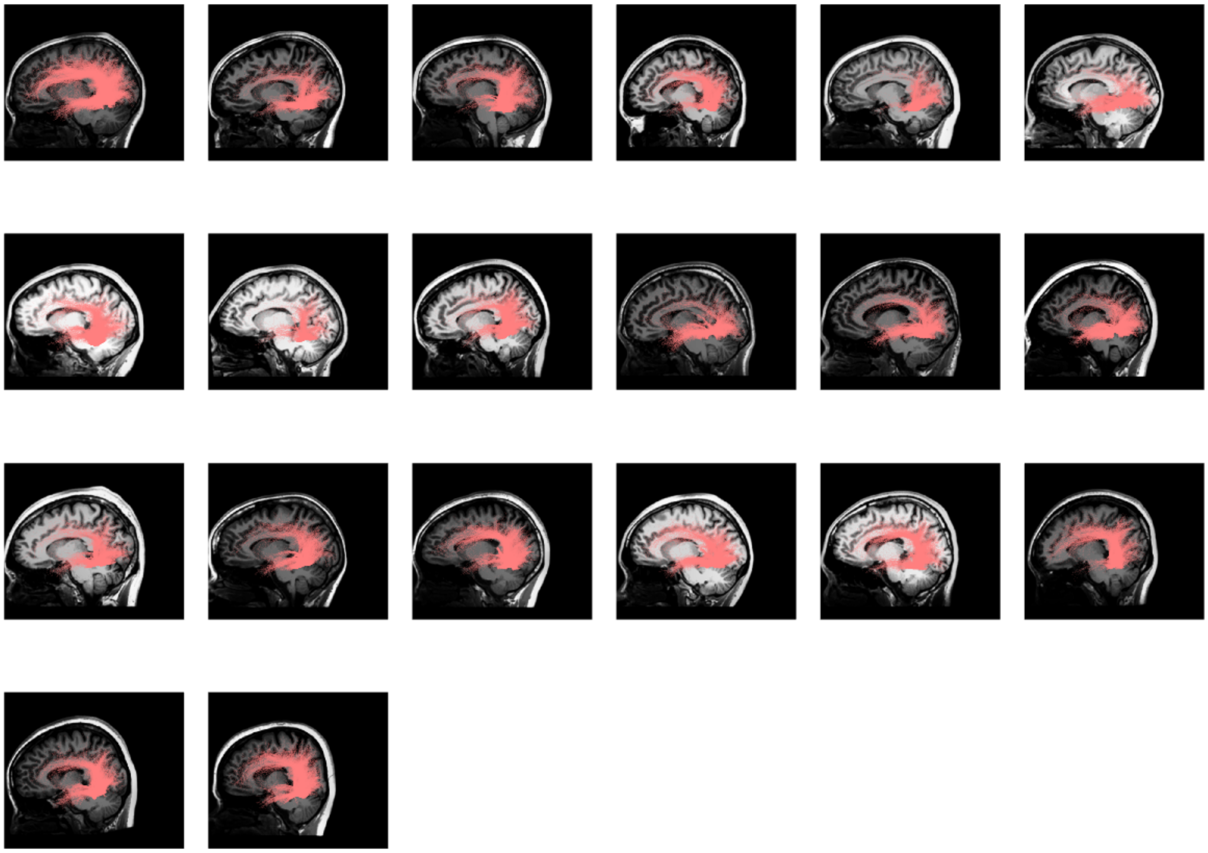

Supplementary Figure 21. White matter connections of mFus-faces in the adult age group organized by age in the left hemisphere.

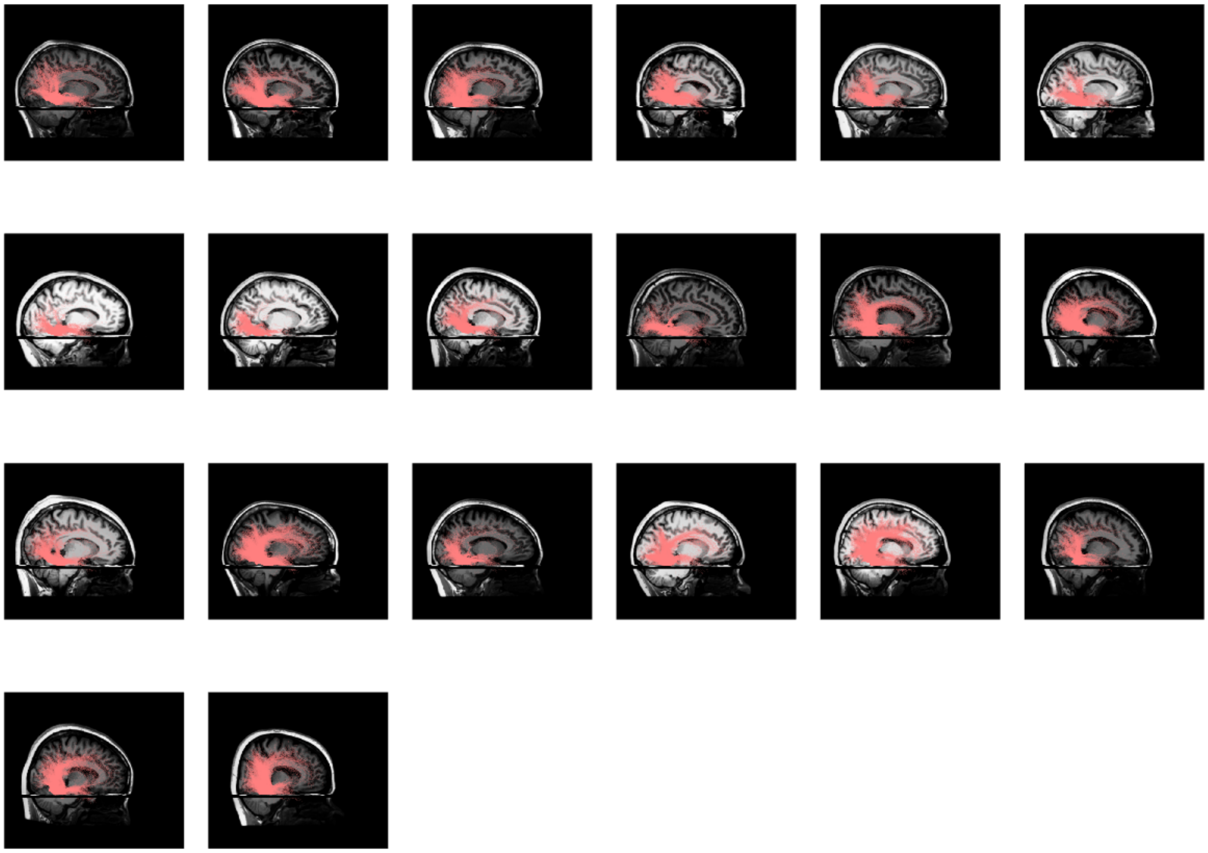

Supplementary Figure 22. White matter connections of mFus-faces in the adult age group organized by age in the right hemisphere.

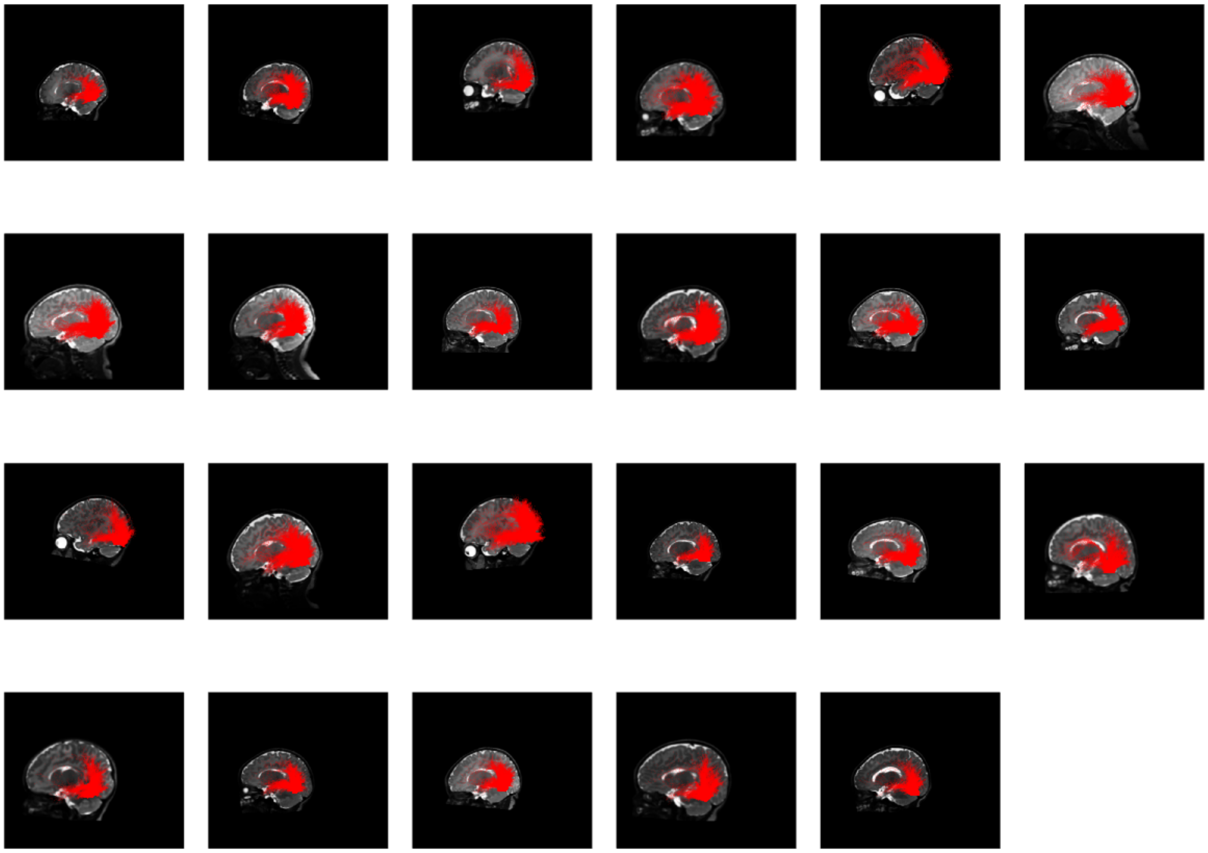

Supplementary Figure 23. White matter connections of pFus-faces in the 0-month age group organized by age in the left hemisphere.

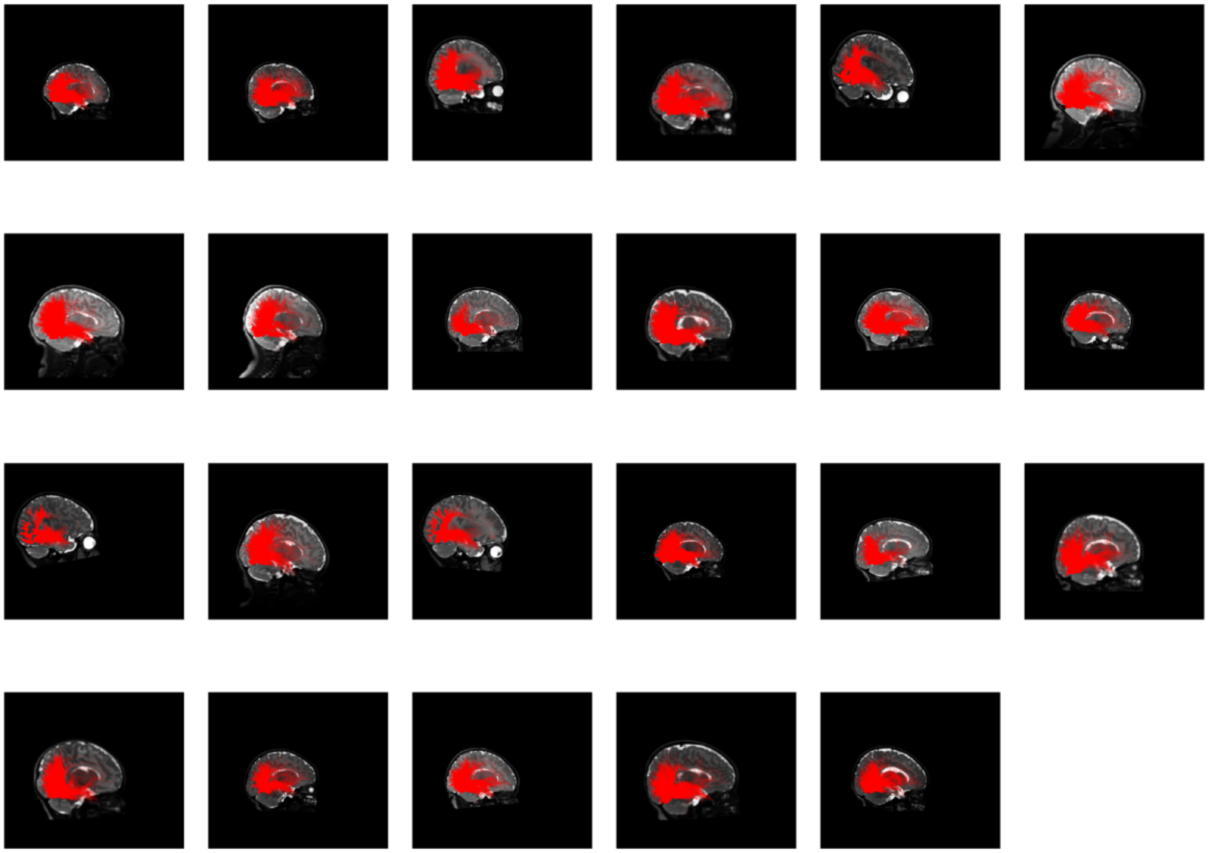

Supplementary Figure 24. White matter connections of pFus-faces in the 0-month age group organized by age in the right hemisphere.

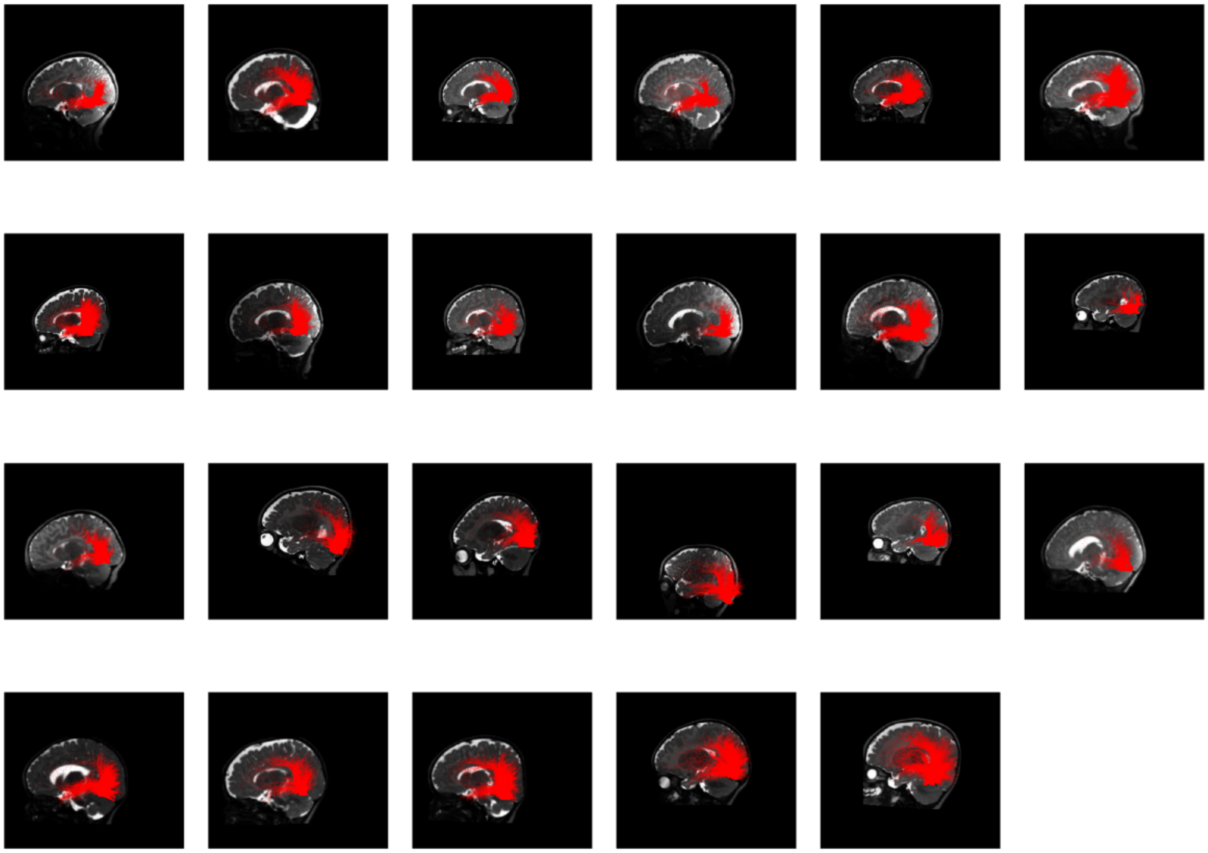

Supplementary Figure 25. White matter connections of pFus-faces in the 3-month age group organized by age in the left hemisphere.

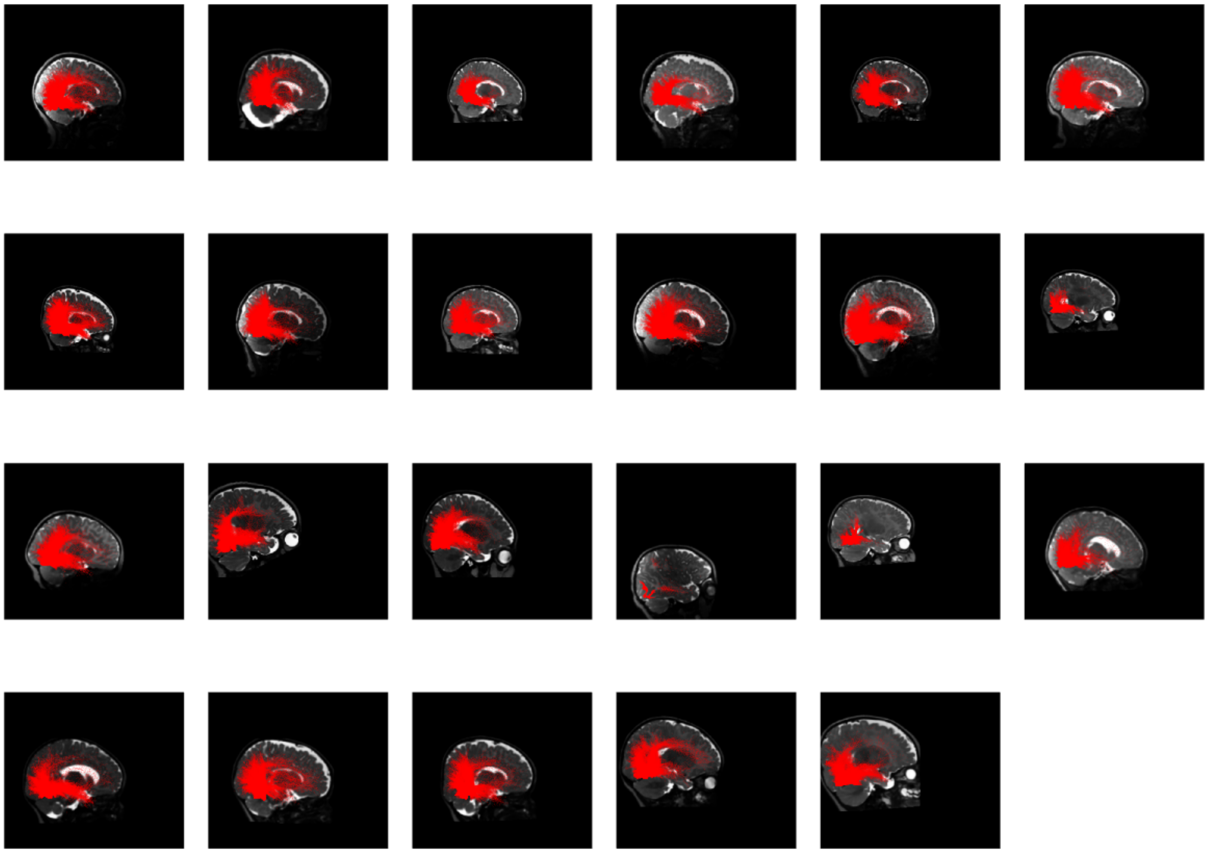

Supplementary Figure 26. White matter connections of pFus-faces in the 3-month age group organized by age in the right hemisphere.

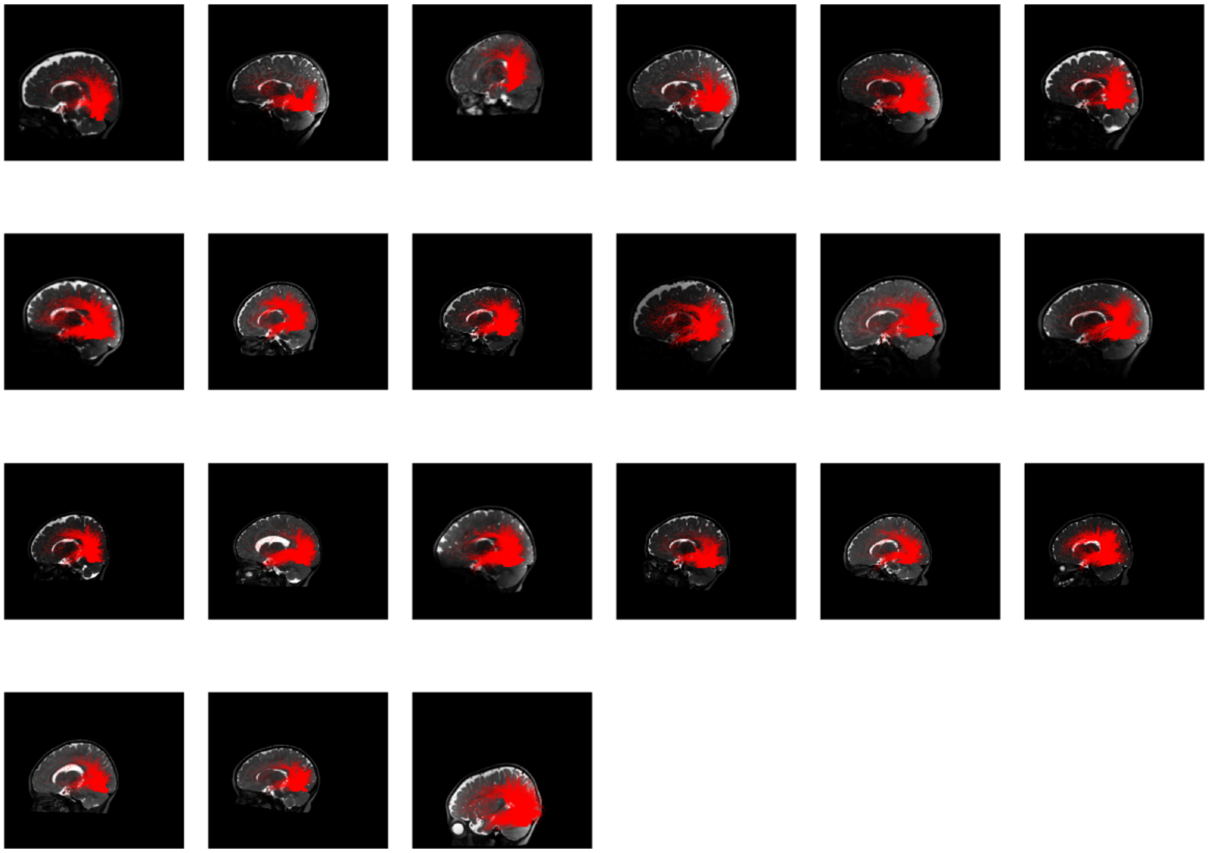

Supplementary Figure 27. White matter connections of pFus-faces in the 6-month age group organized by age in the left hemisphere.

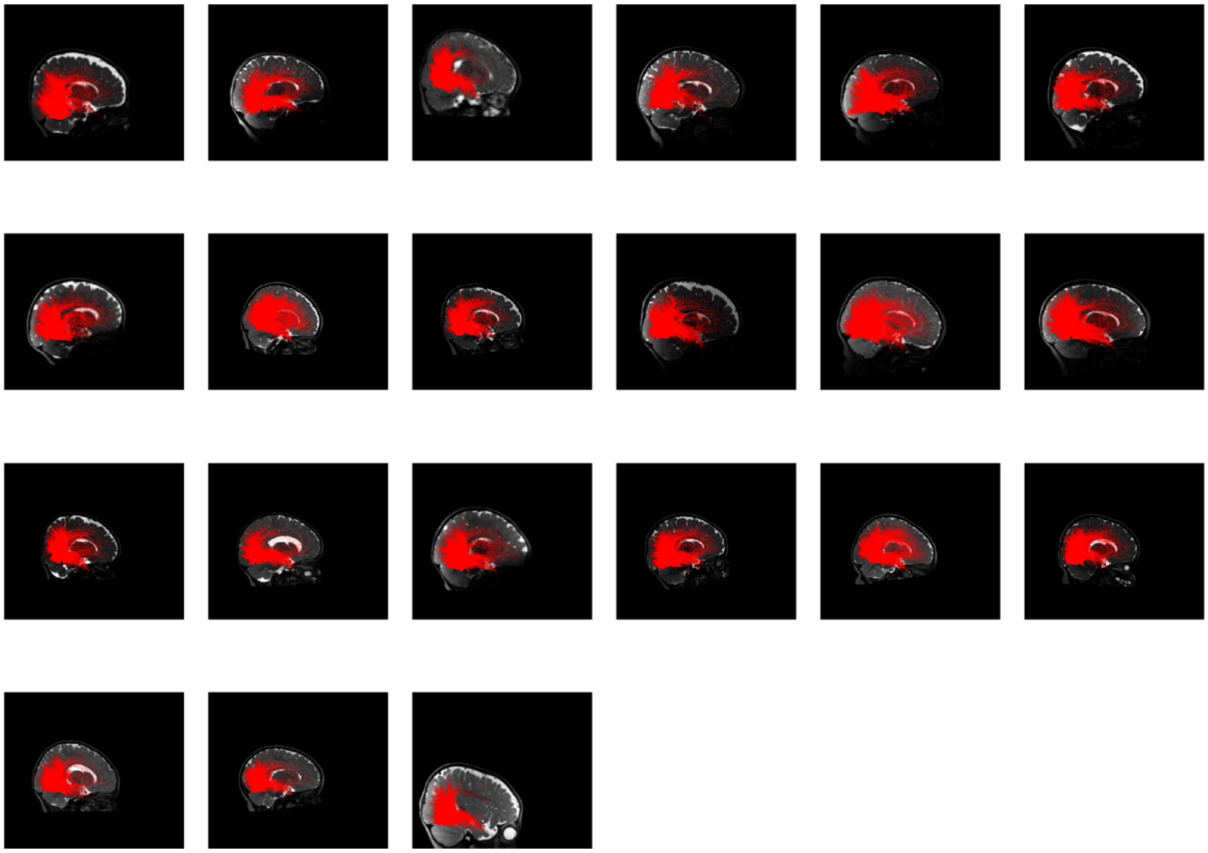

Supplementary Figure 28. White matter connections of pFus-faces in the 6-month age group organized by age in the right hemisphere.

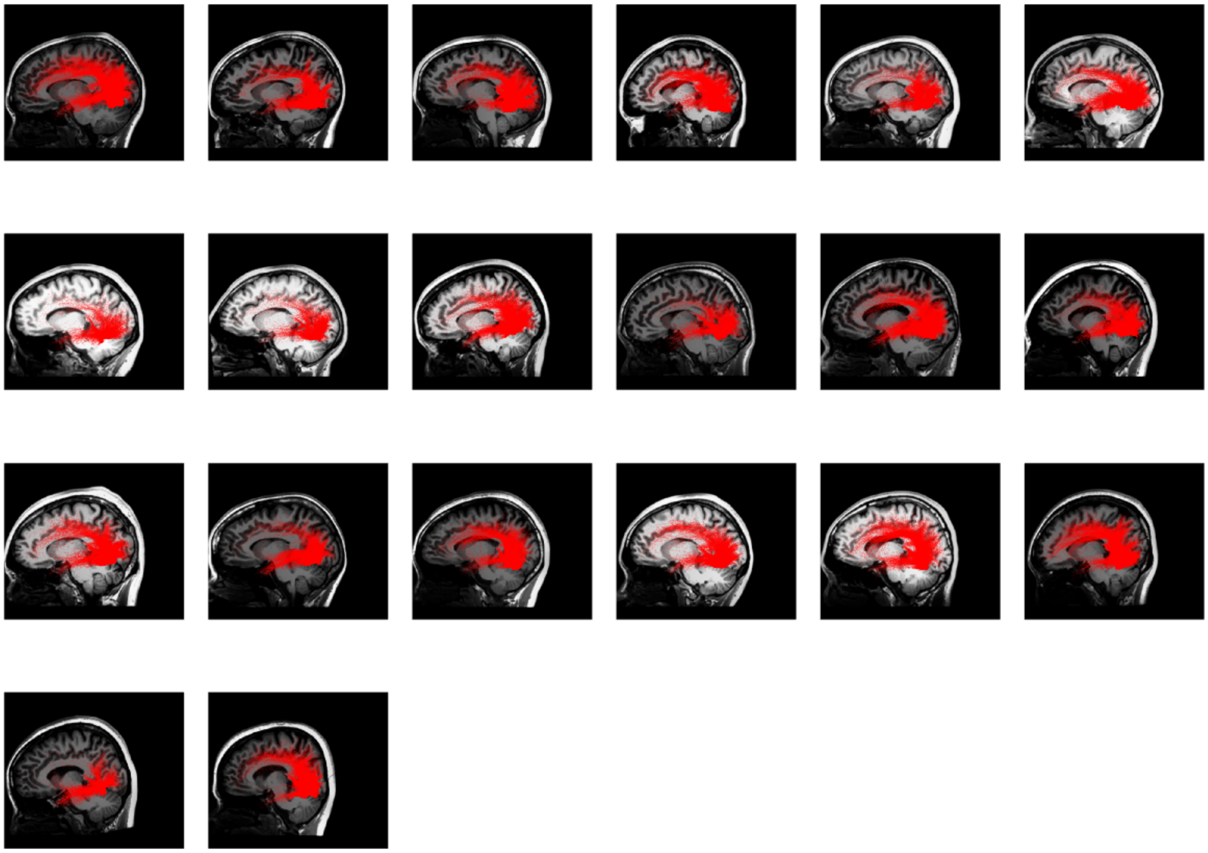

Supplementary Figure 29. White matter connections of pFus-faces in the adult age group organized by age in the left hemisphere.

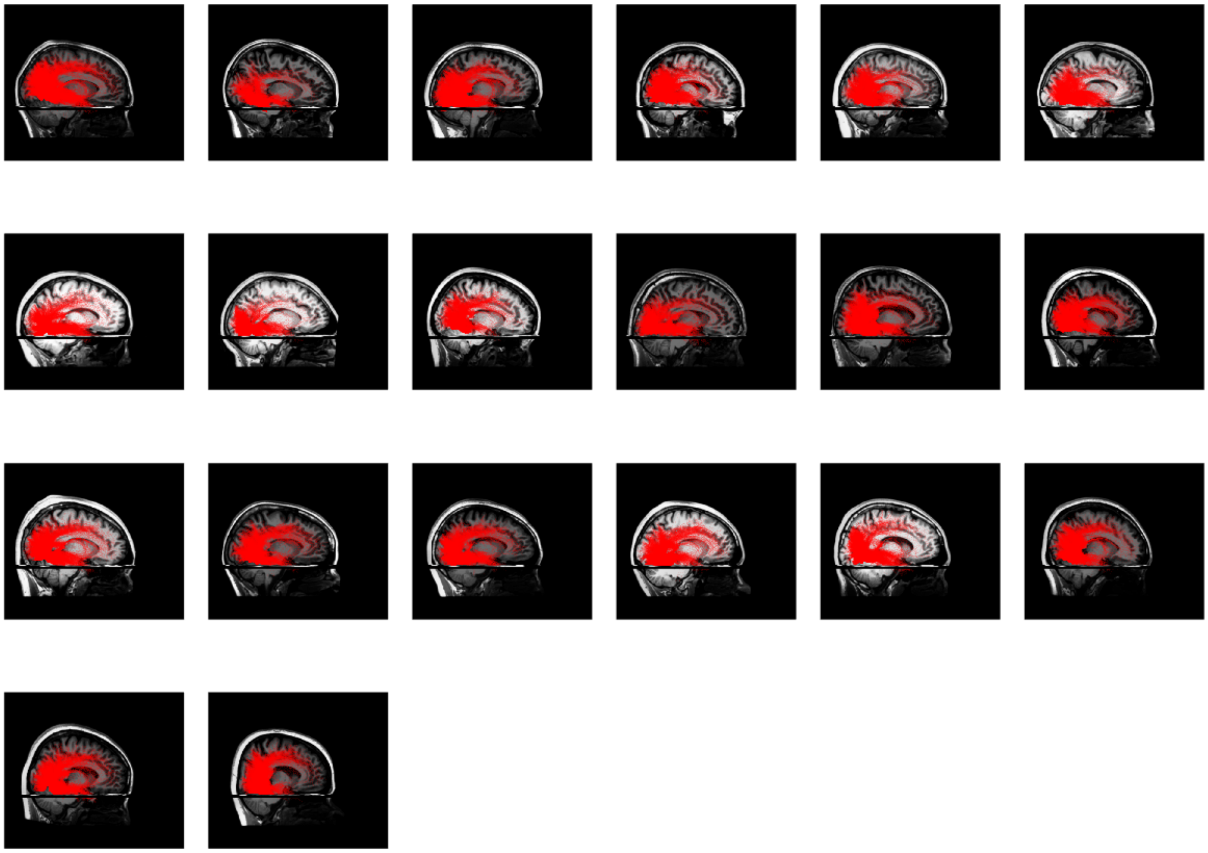

Supplementary Figure 30. White matter connections of pFus-faces in the adult age group organized by age in the right hemisphere.

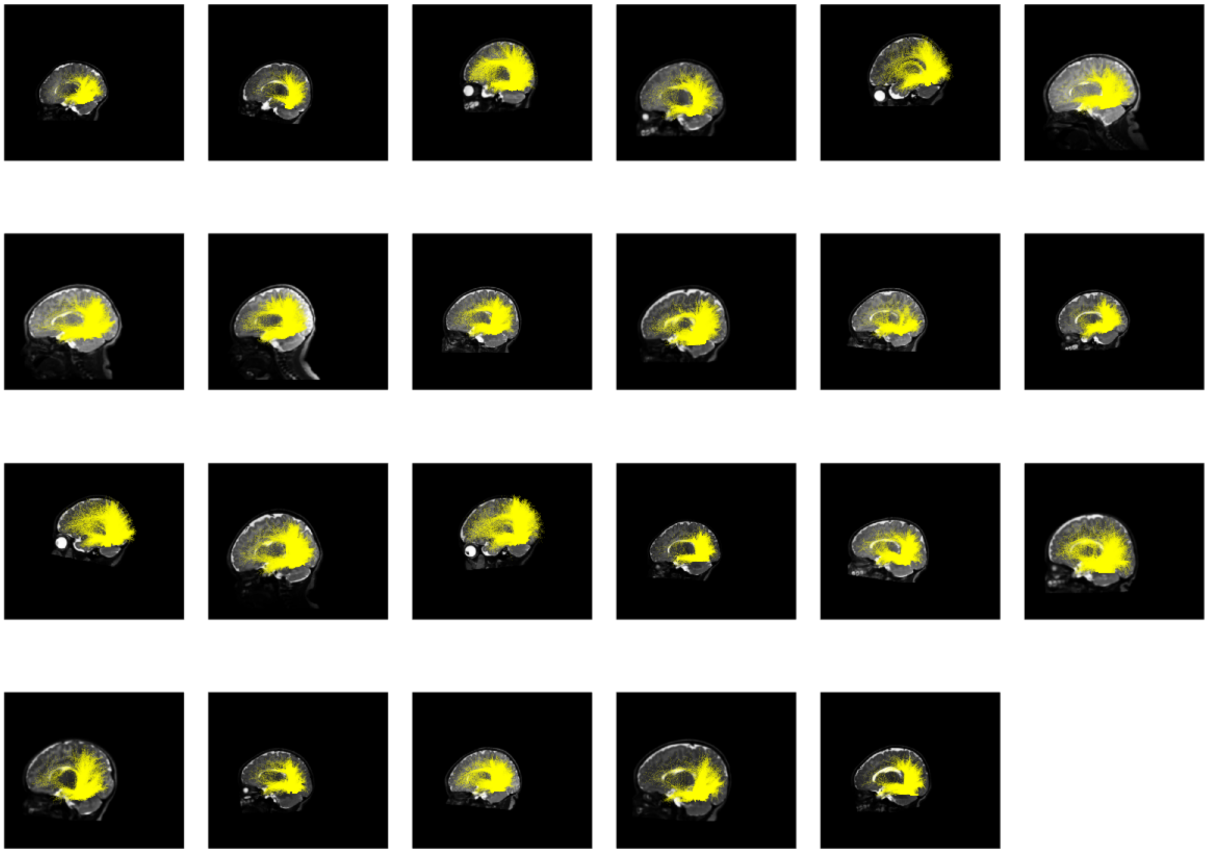

Supplementary Figure 31. White matter connections of OTS-bodies words in the 0-month age group organized by age in the left hemisphere.

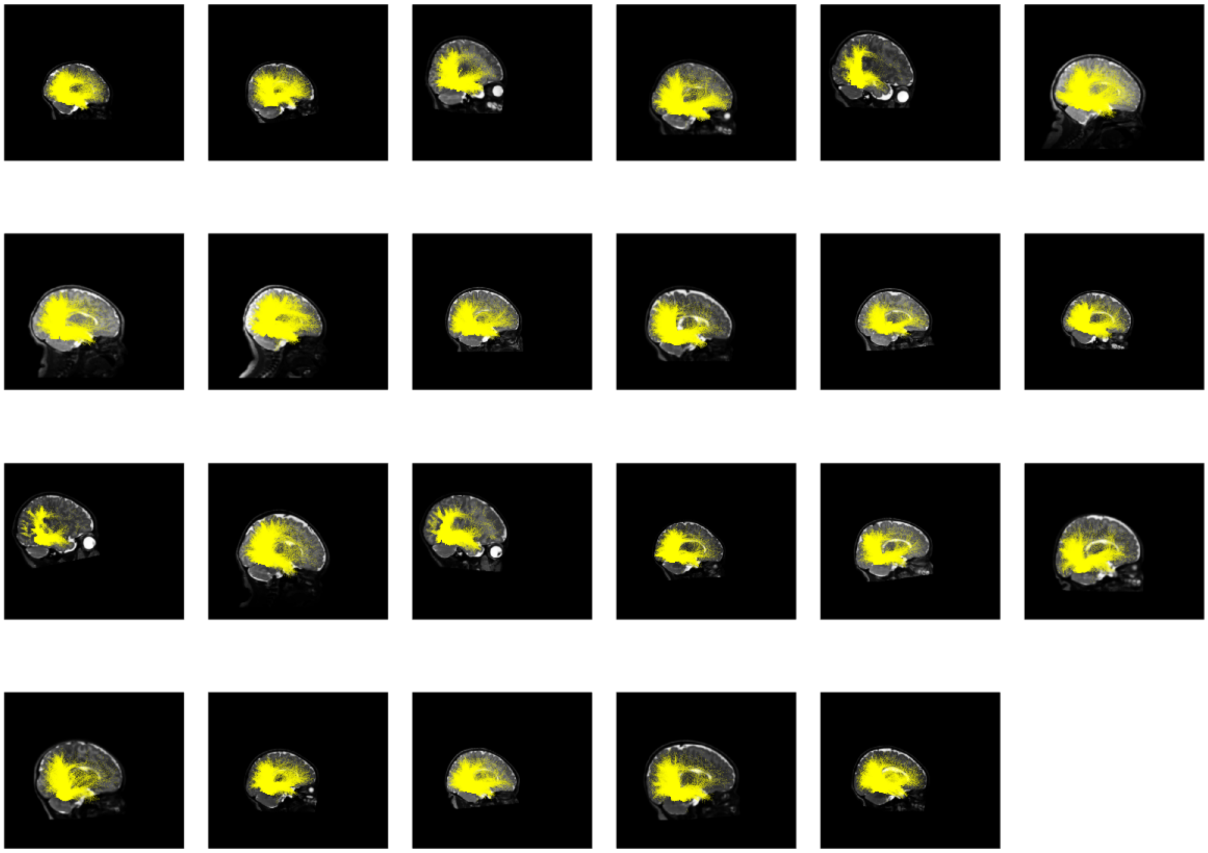

Supplementary Figure 32. White matter connections of OTS-bodies words in the 0-month age group organized by age in the right hemisphere.

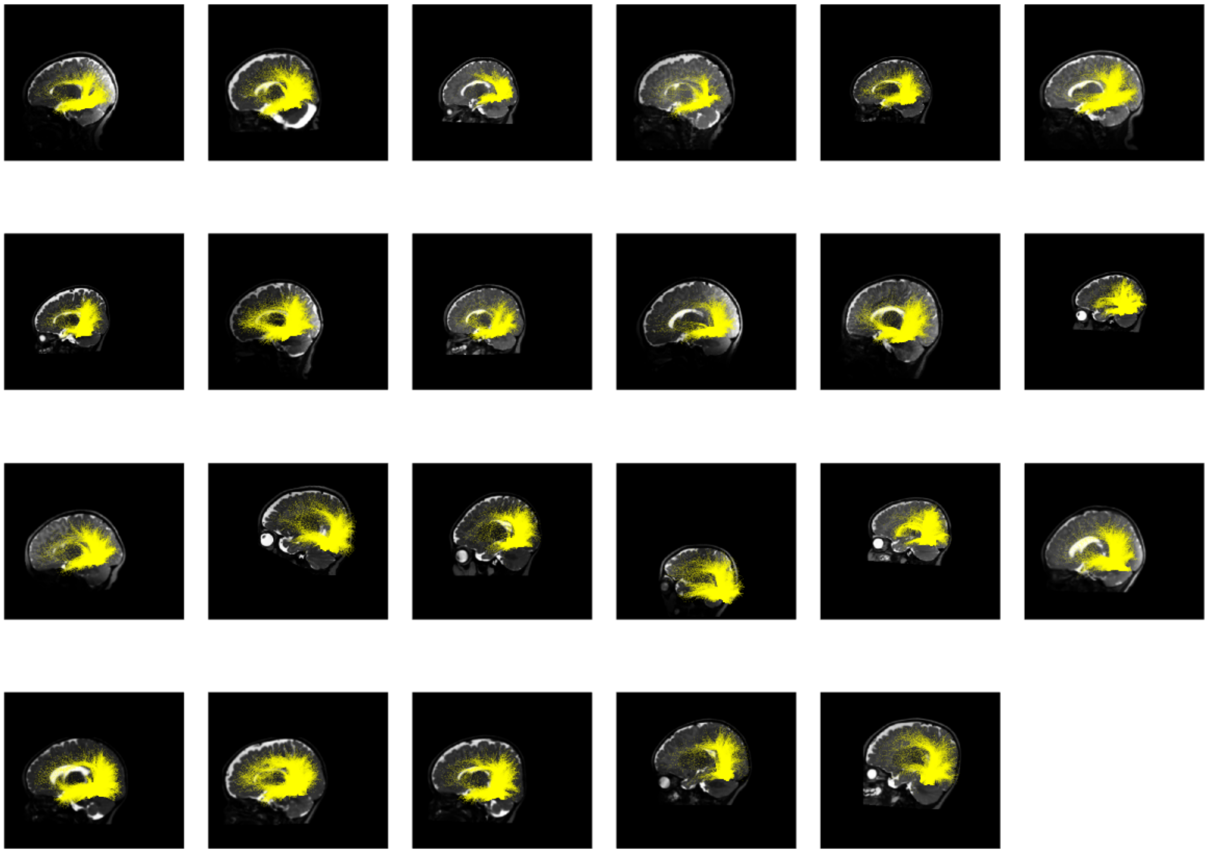

Supplementary Figure 33. White matter connections of OTS-bodies words in the 3-month age group organized by age in the left hemisphere.

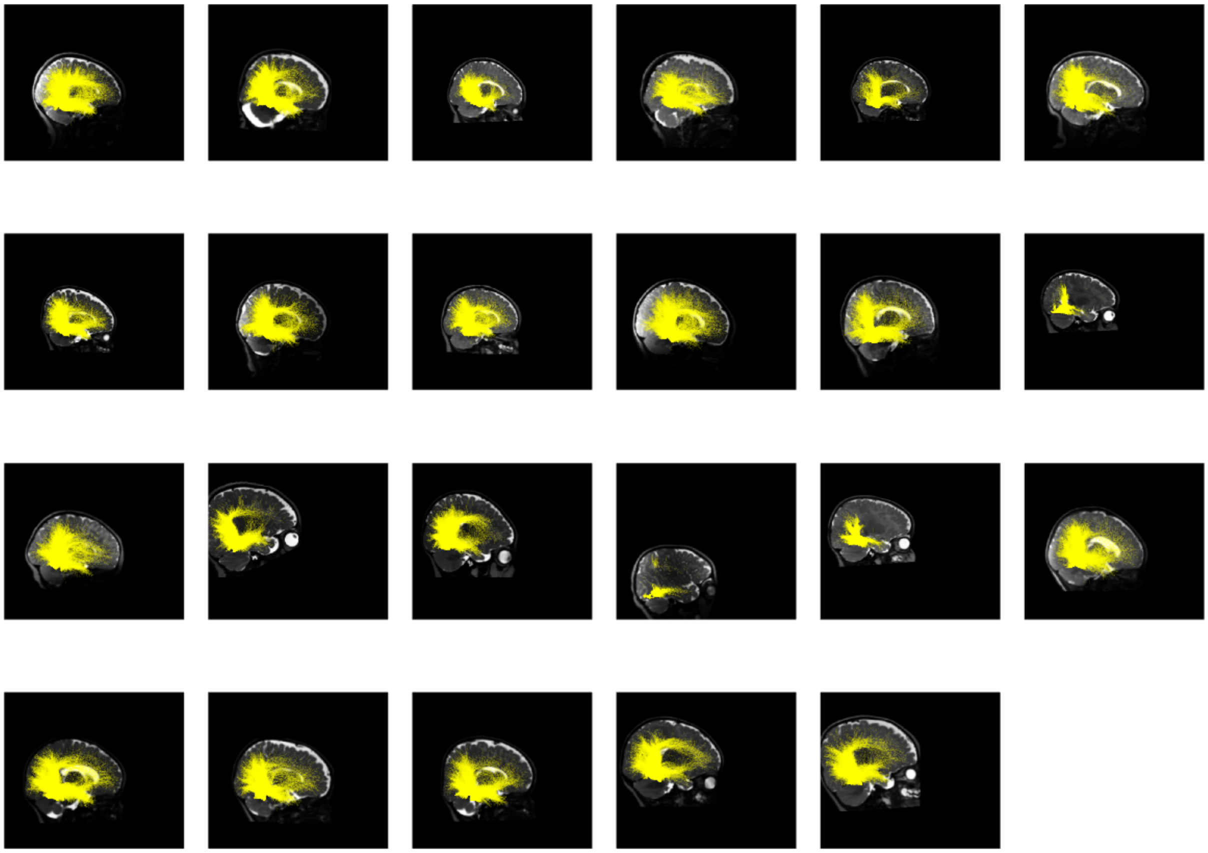

Supplementary Figure 34. White matter connections of OTS-bodies words in the 3-month age group organized by age in the right hemisphere.

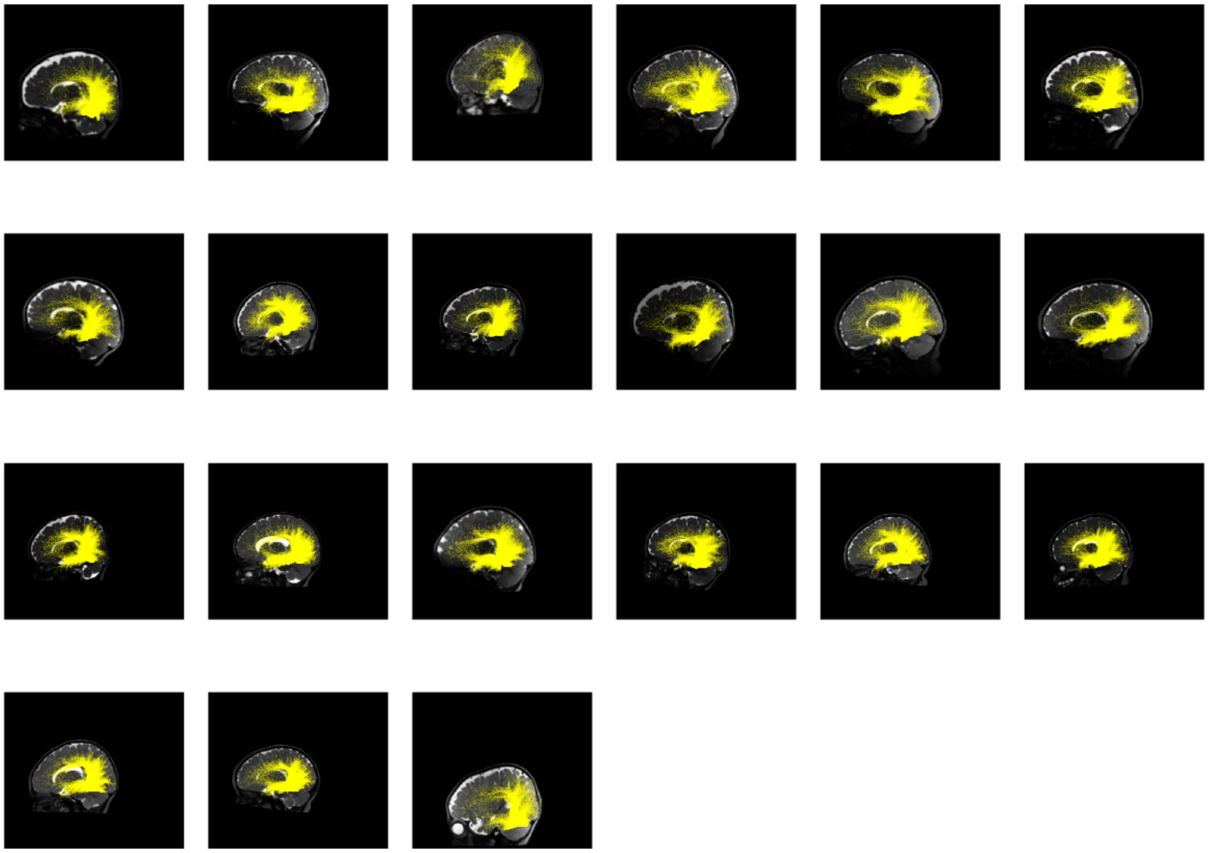

Supplementary Figure 35. White matter connections of OTS-bodies words in the 6-month age group organized by age in the left hemisphere.

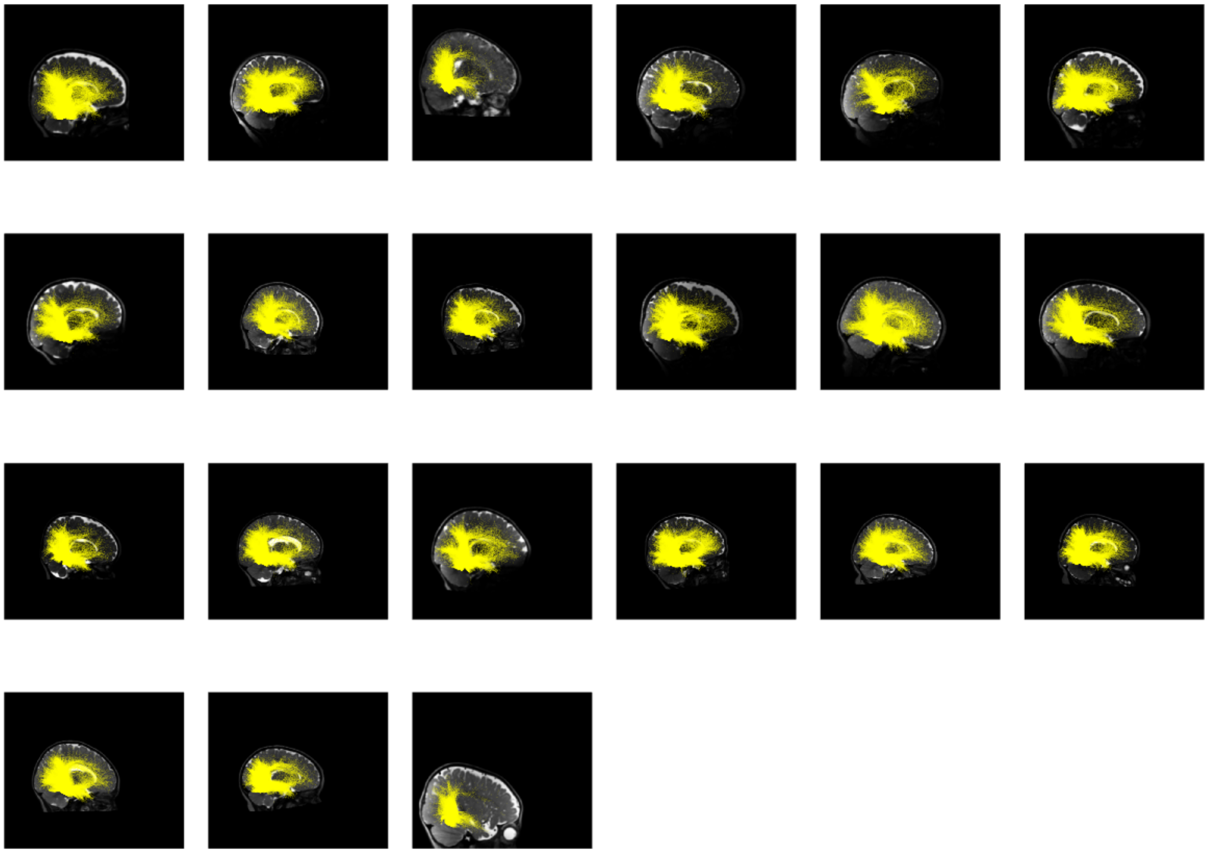

Supplementary Figure 36. White matter connections of OTS-bodies words in the 6-month age group organized by age in the right hemisphere.

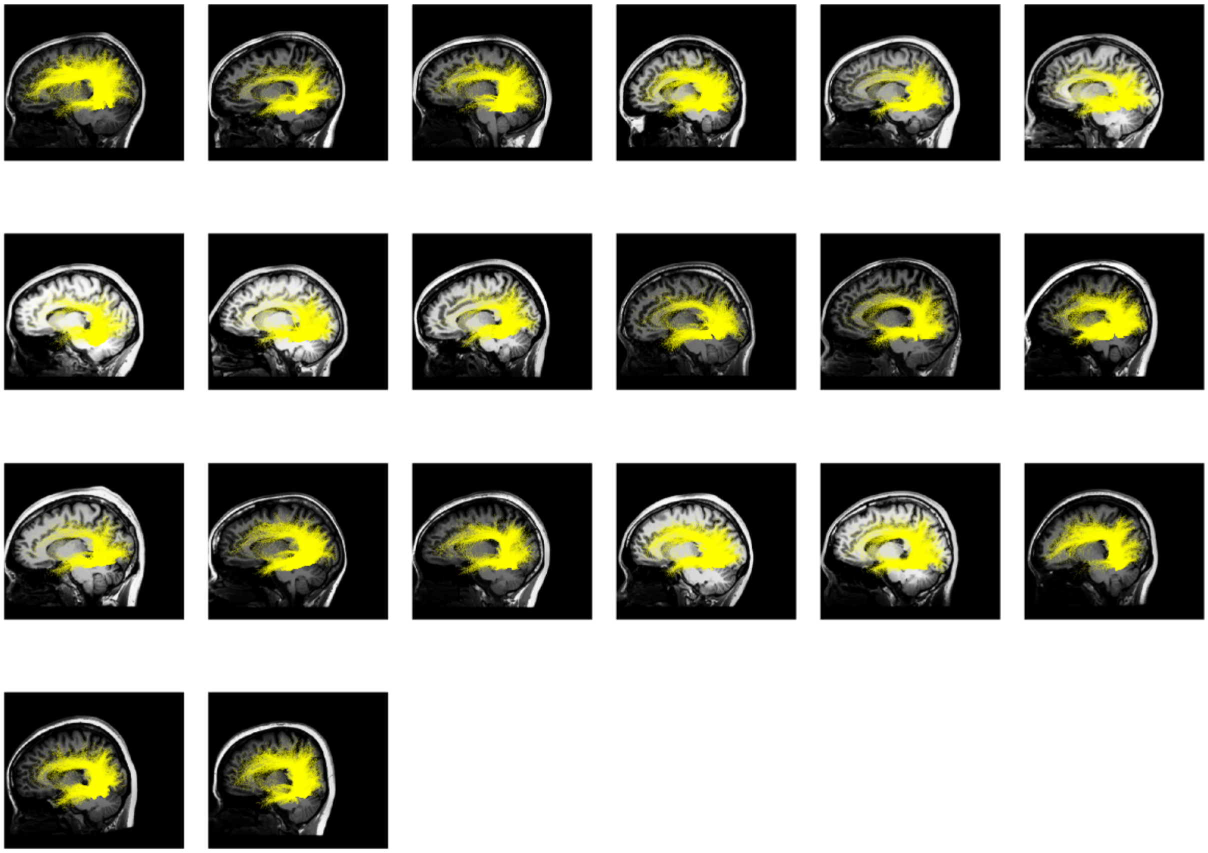

Supplementary Figure 37. White matter connections of OTS-bodies in the adult age group organized by age in the left hemisphere.

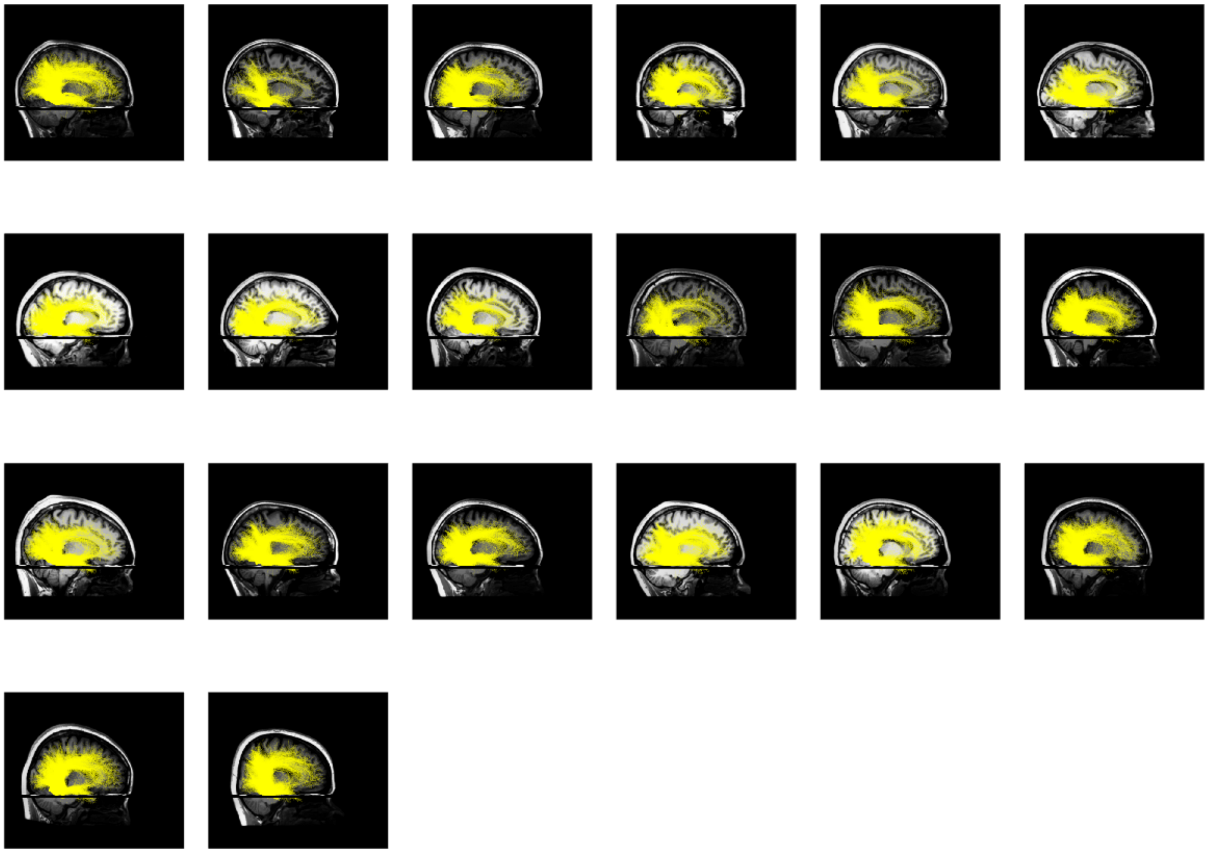

Supplementary Figure 38. White matter connections of OTS-bodies in the adult age group organized by age in the right hemisphere.

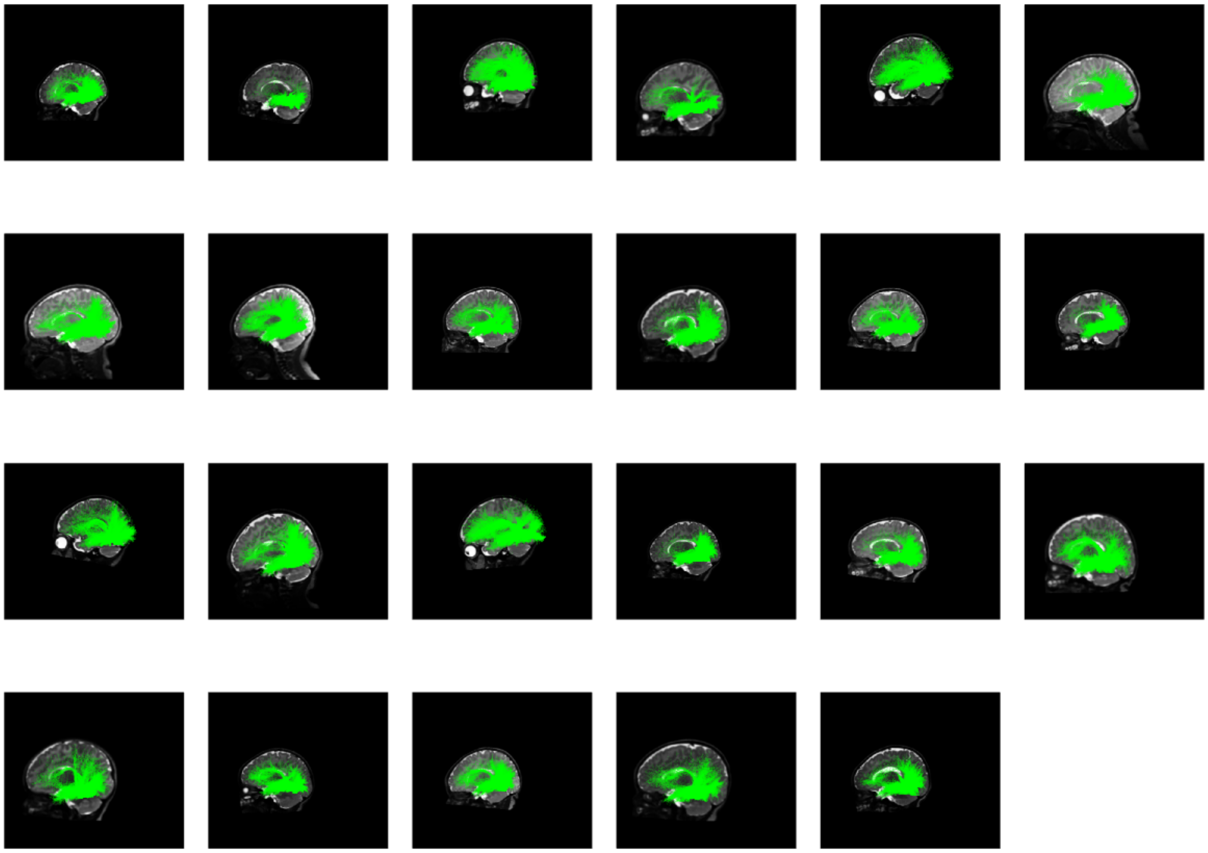

Supplementary Figure 39. White matter connections of CoS-places in the 0-month age group organized by age in the left hemisphere.

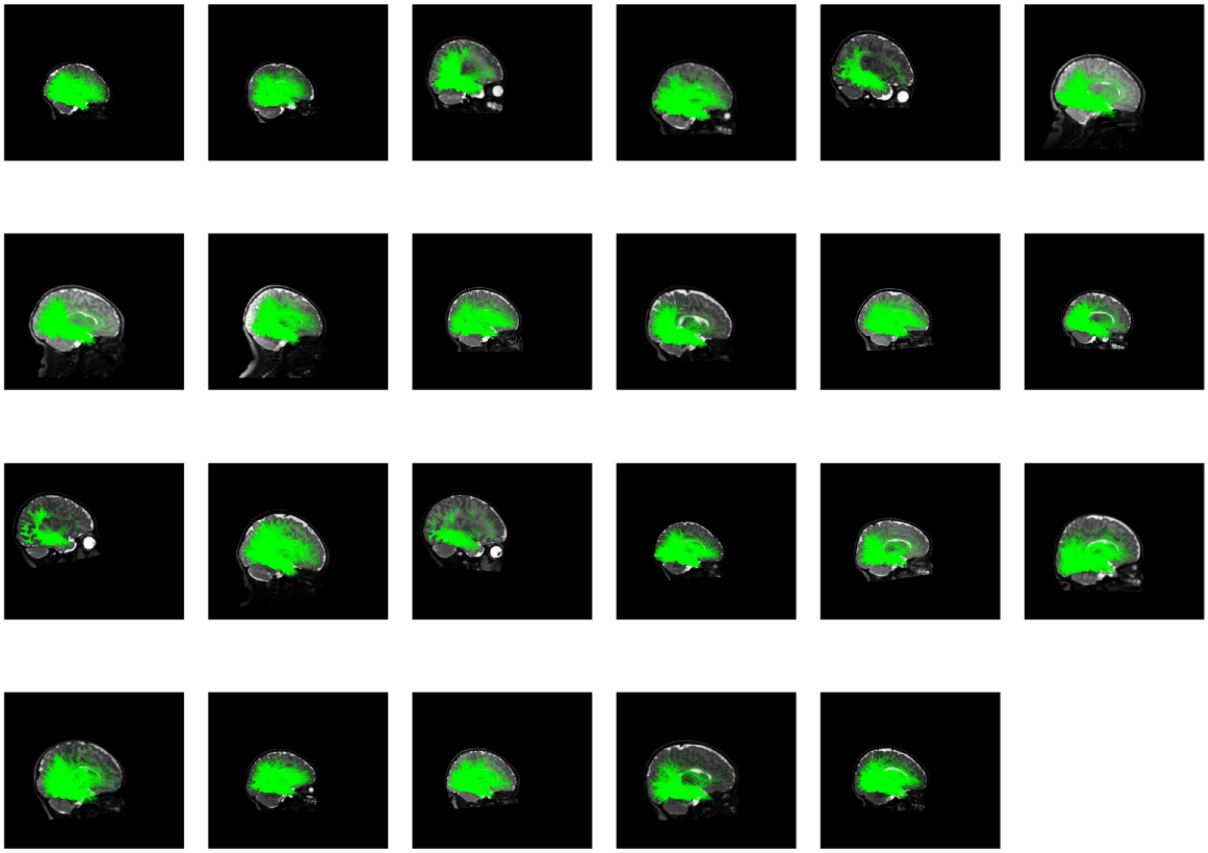

Supplementary Figure 40. White matter connections of CoS-places in the 0-month age group organized by age in the right hemisphere.

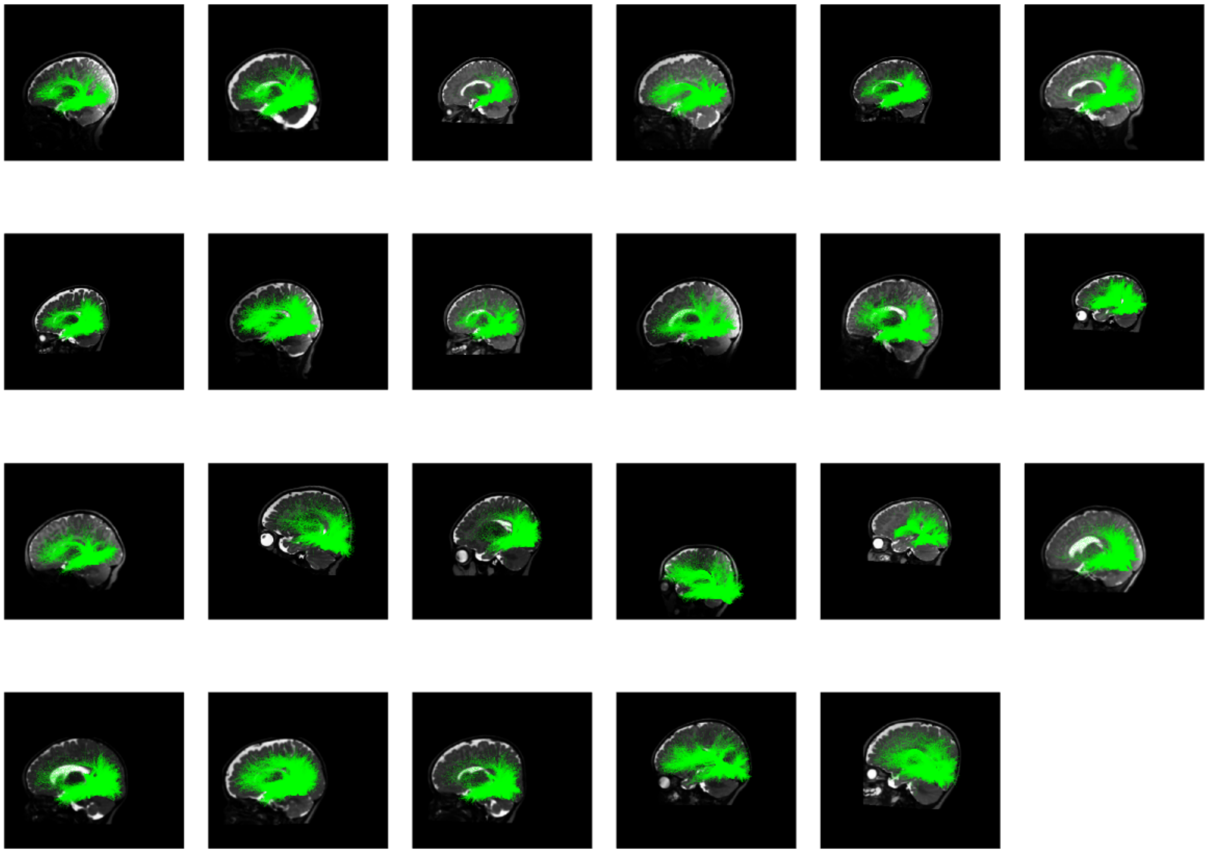

Supplementary Figure 41. White matter connections of CoS-places in the 3-month age group organized by age in the left hemisphere.

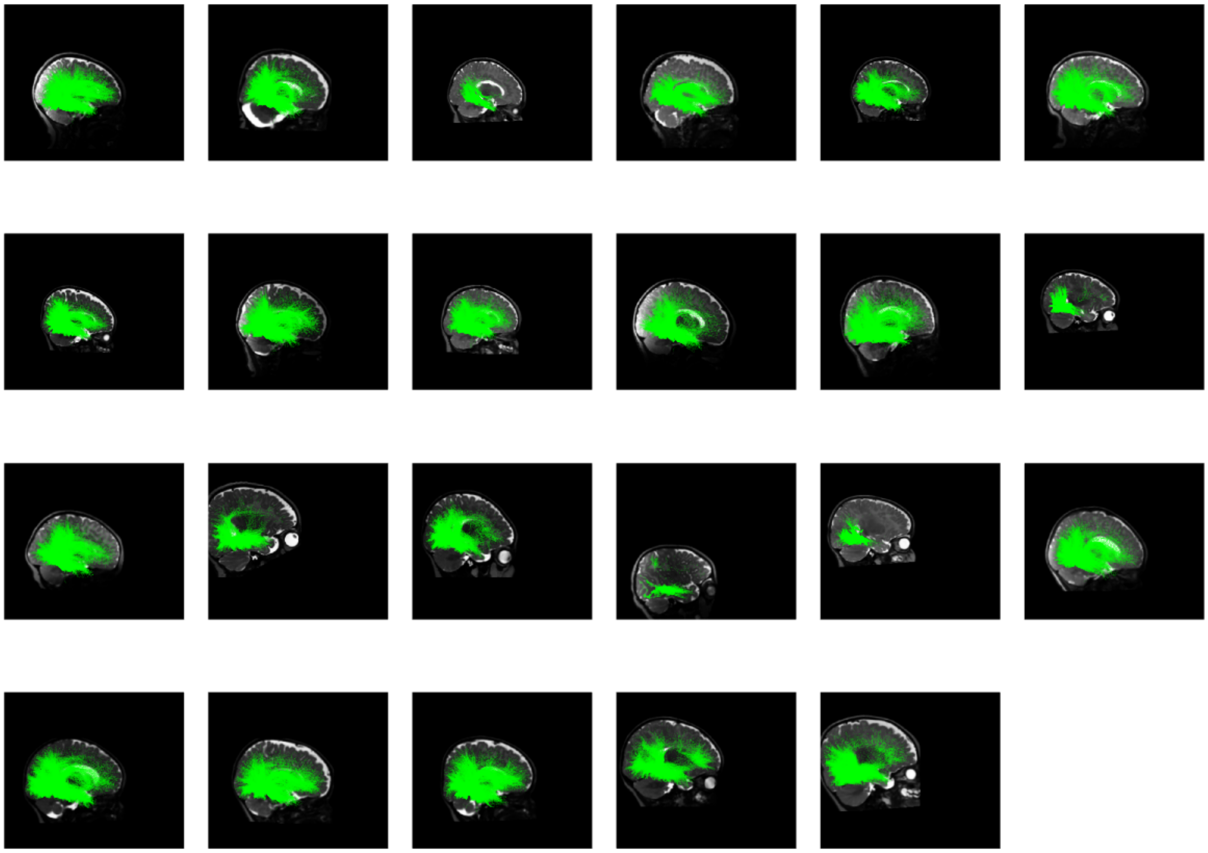

Supplementary Figure 42. White matter connections of CoS-places in the 3-month age group organized by age in the right hemisphere.

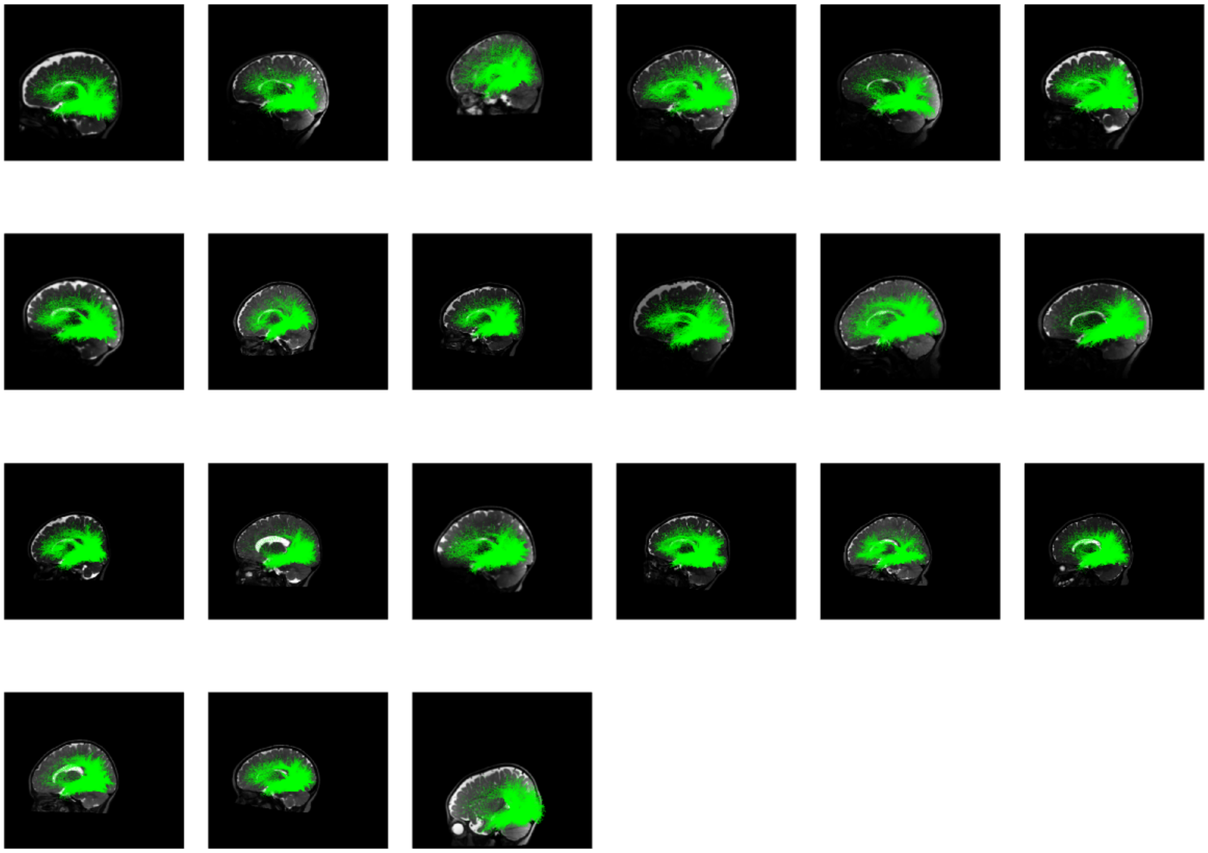

Supplementary Figure 43. White matter connections of CoS-places in the 6-month age group organized by age in the left hemisphere.

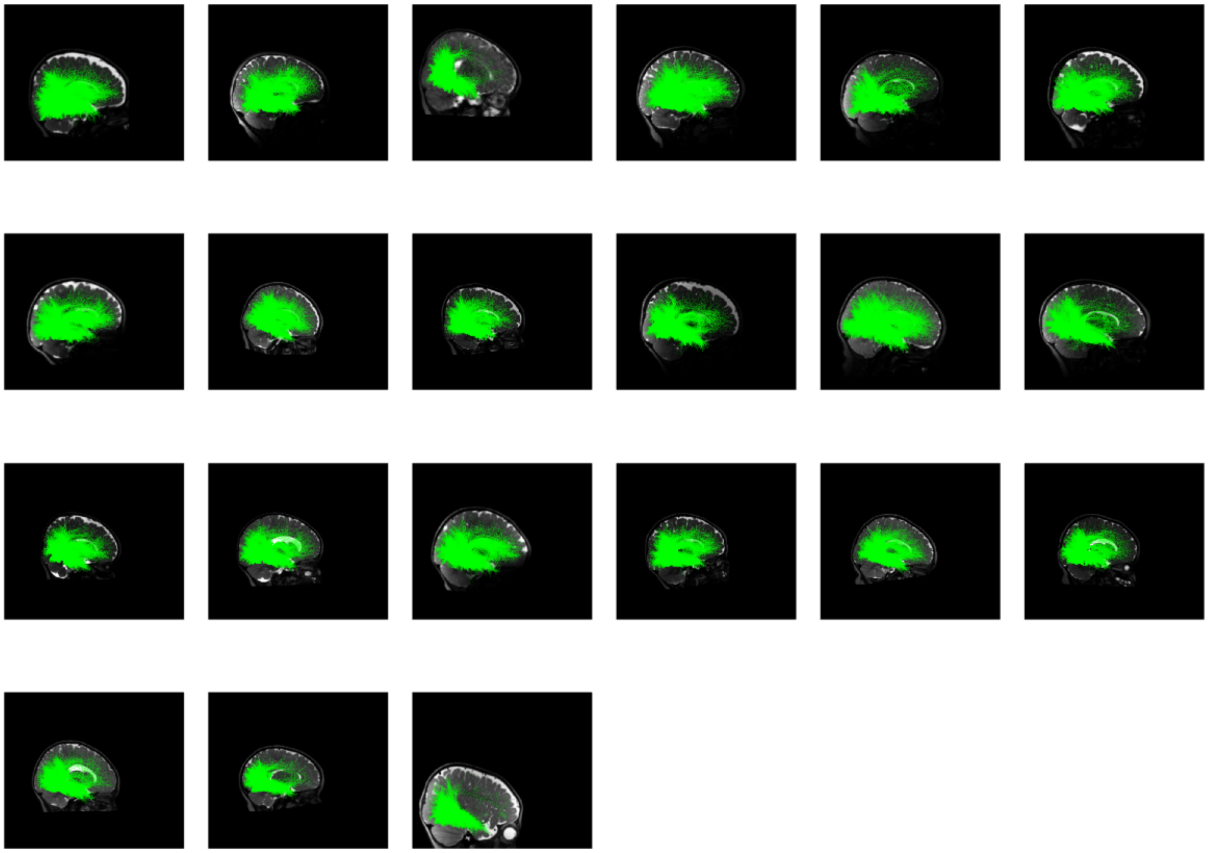

Supplementary Figure 44. White matter connections of CoS-places in the 6-month age group organized by age in the right hemisphere.

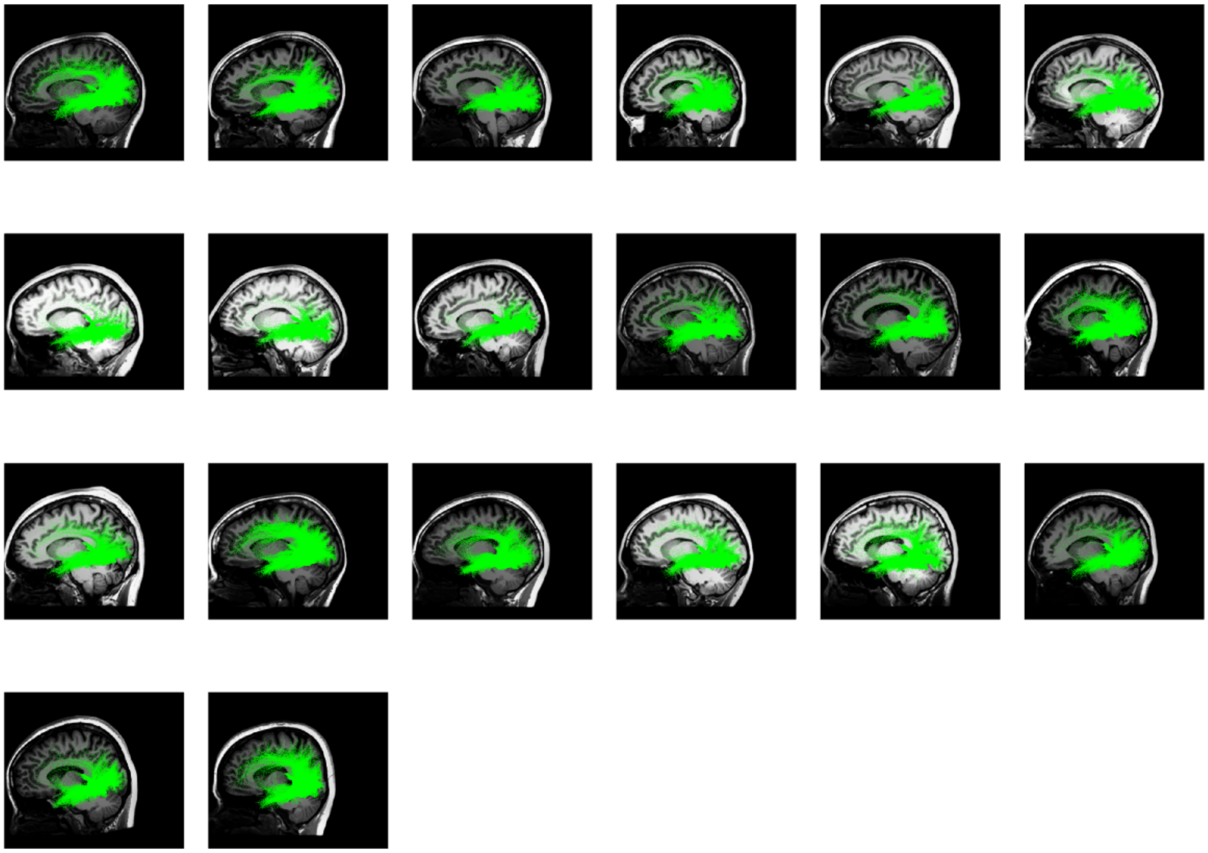

Supplementary Figure 45. White matter connections of CoS-places in the adult age group organized by age in the left hemisphere.

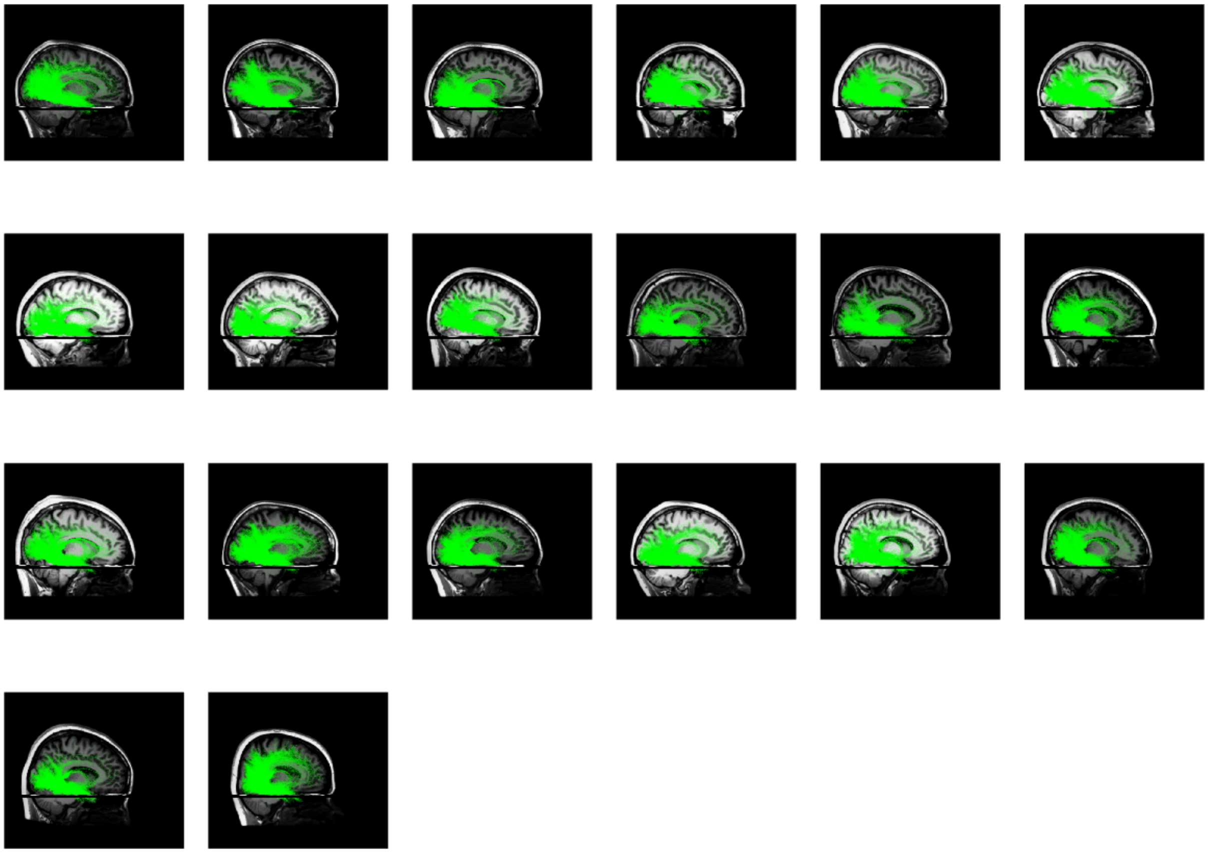

Supplementary Figure 46. White matter connections of CoS-places in the adult age group organized by age in the right hemisphere.

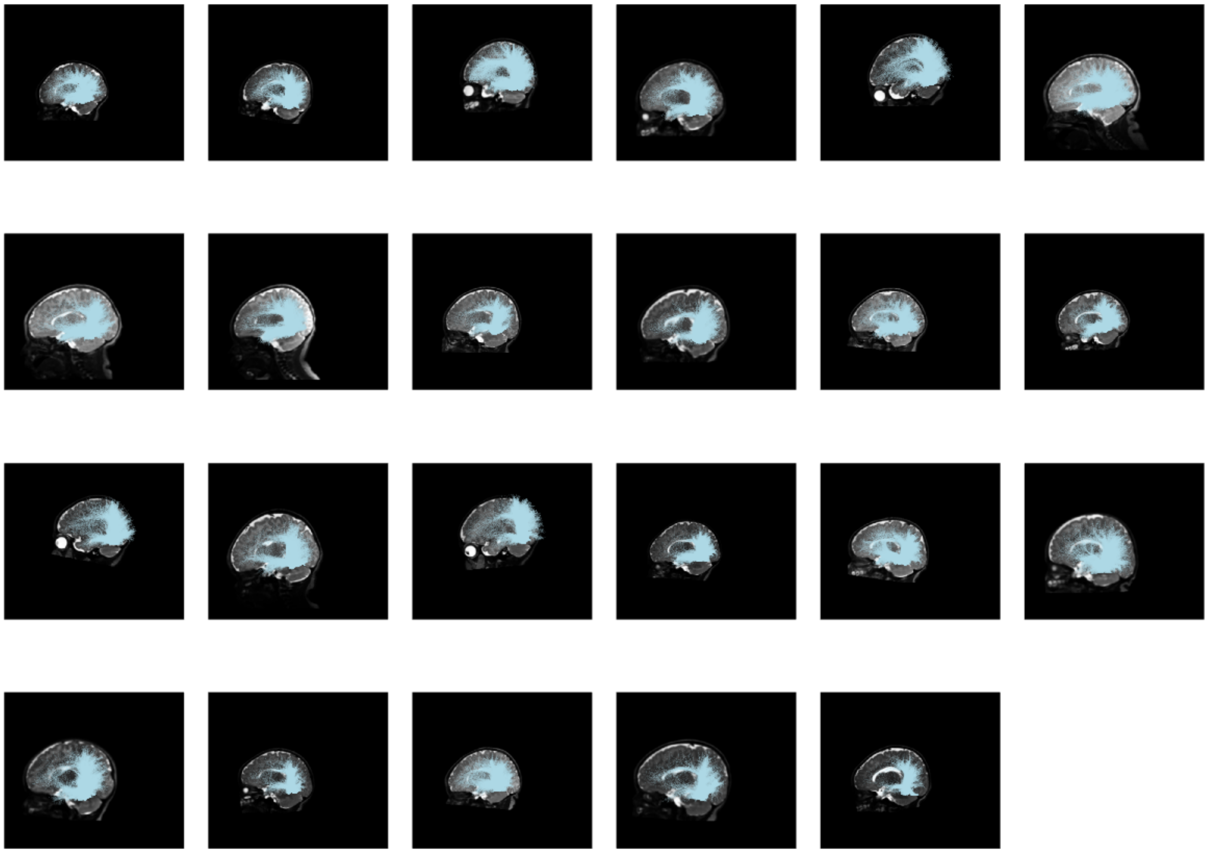

Supplementary Figure 47. White matter connections of mOTS-words in the 0-month age group organized by age in the left hemisphere.

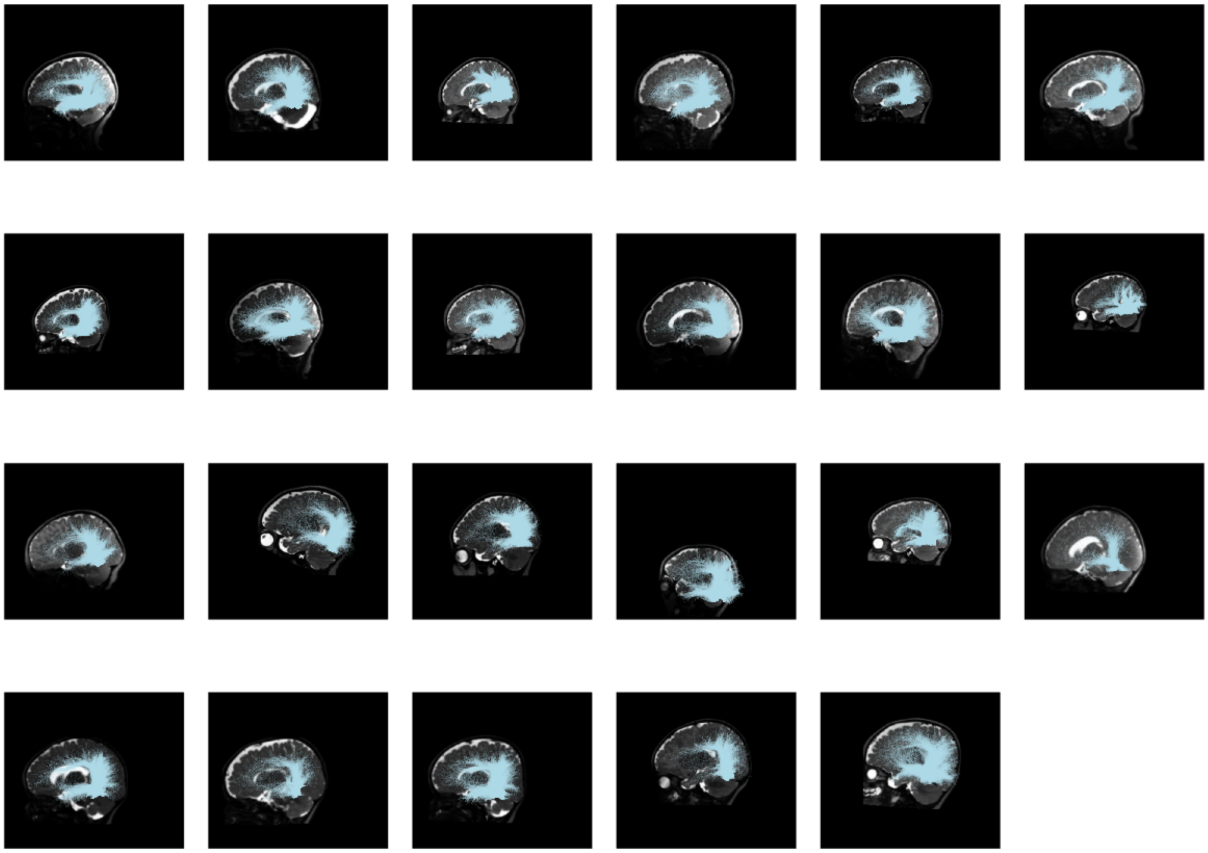

Supplementary Figure 48. White matter connections of mOTS-words in the 3-month age group organized by age in the left hemisphere.

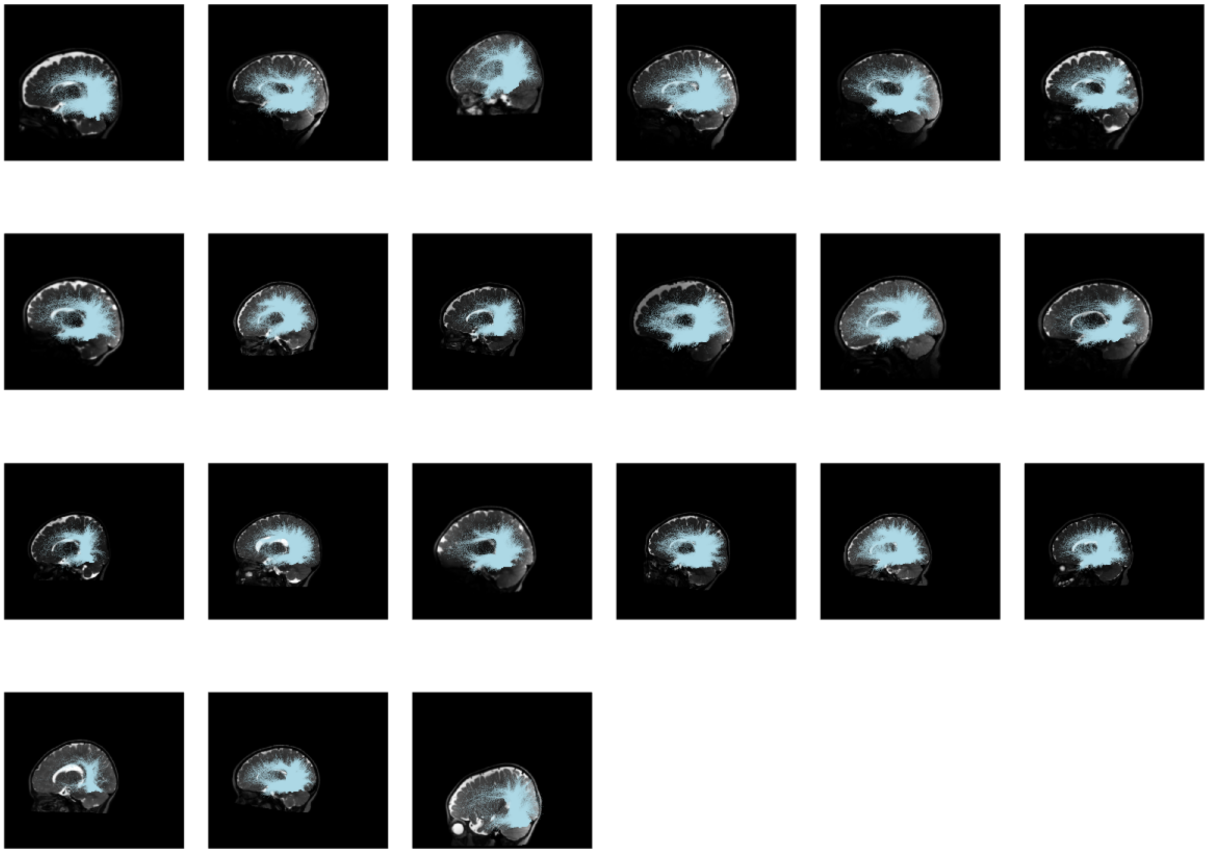

Supplementary Figure 49. White matter connections of mOTS-words in the 6-month age group organized by age in the left hemisphere.

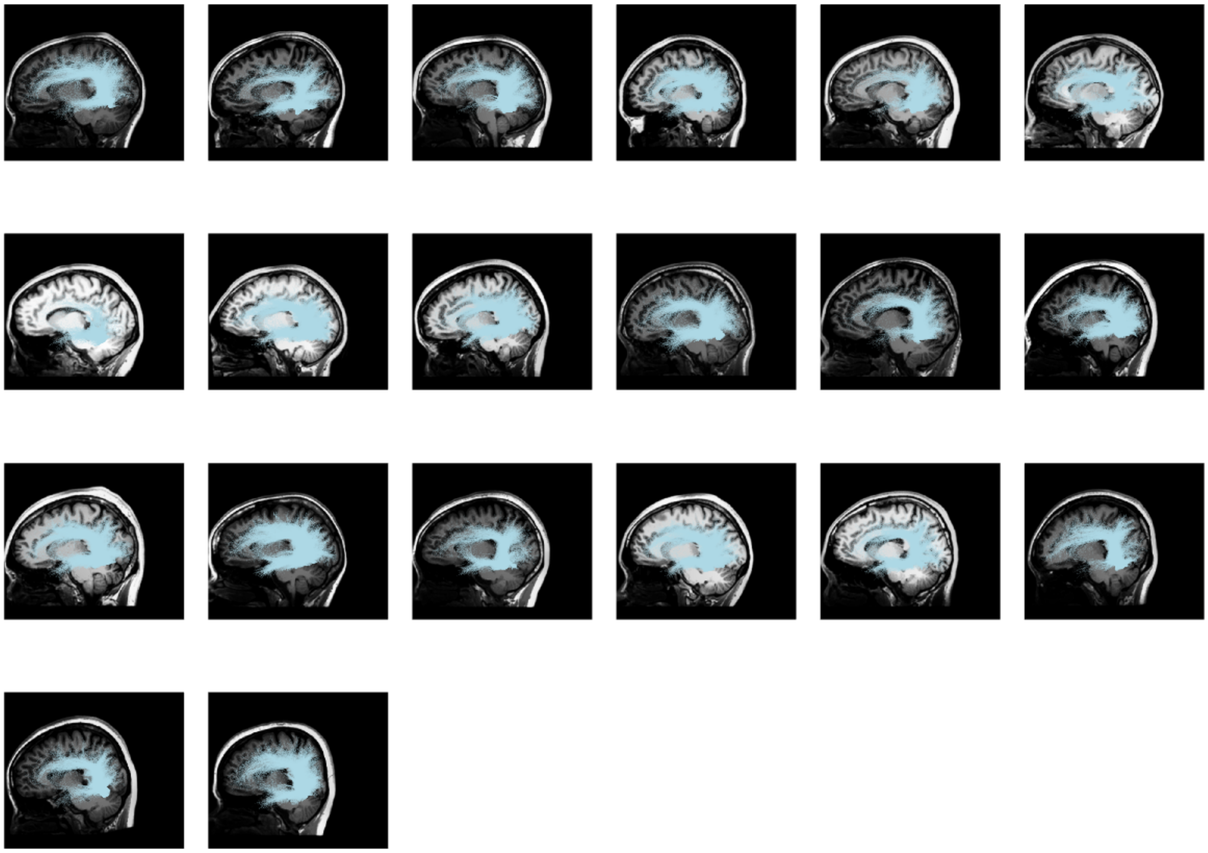

Supplementary Figure 50. White matter connections of mOTS-words in the adult age group organized by age in the left hemisphere.

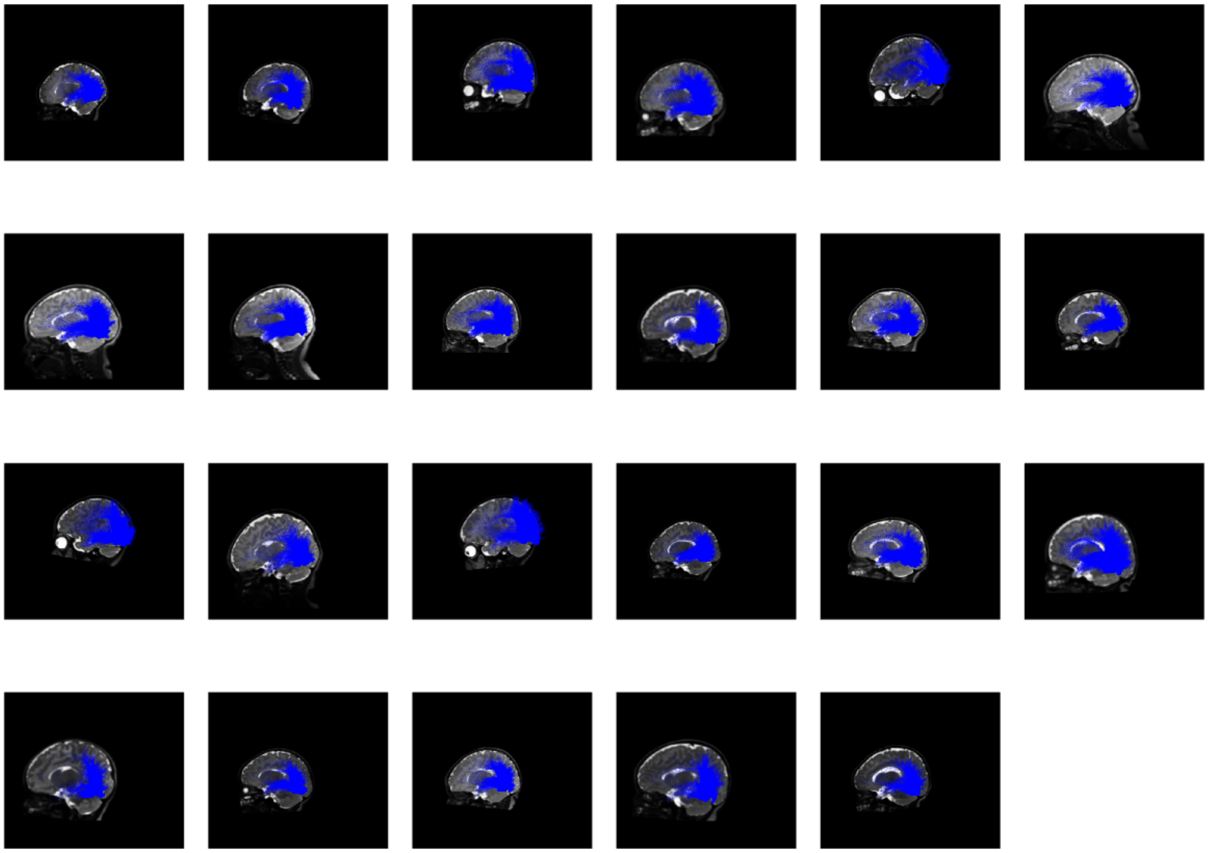

Supplementary Figure 51. White matter connections of pOTS-words in the 0-month age group organized by age in the left hemisphere.

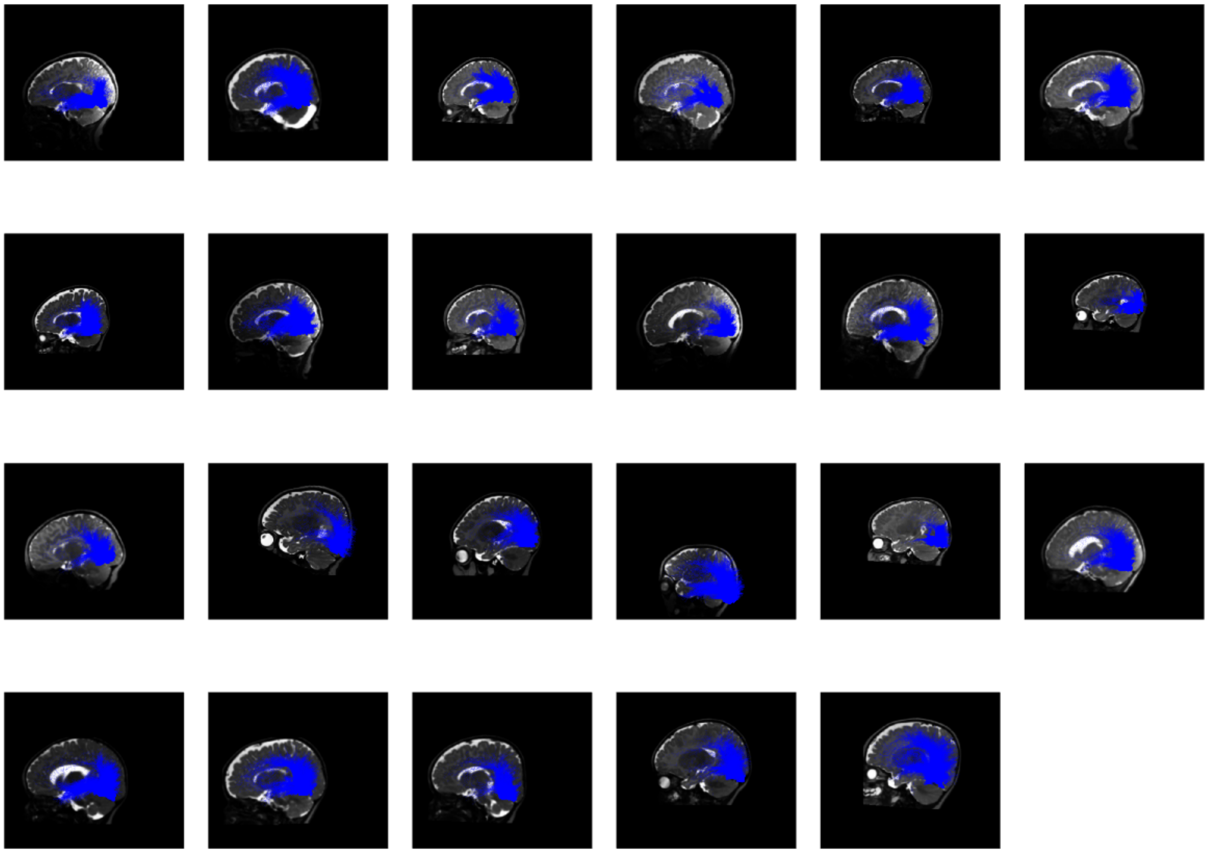

Supplementary Figure 52. White matter connections of pOTS-words in the 3-month age group organized by age in the left hemisphere.

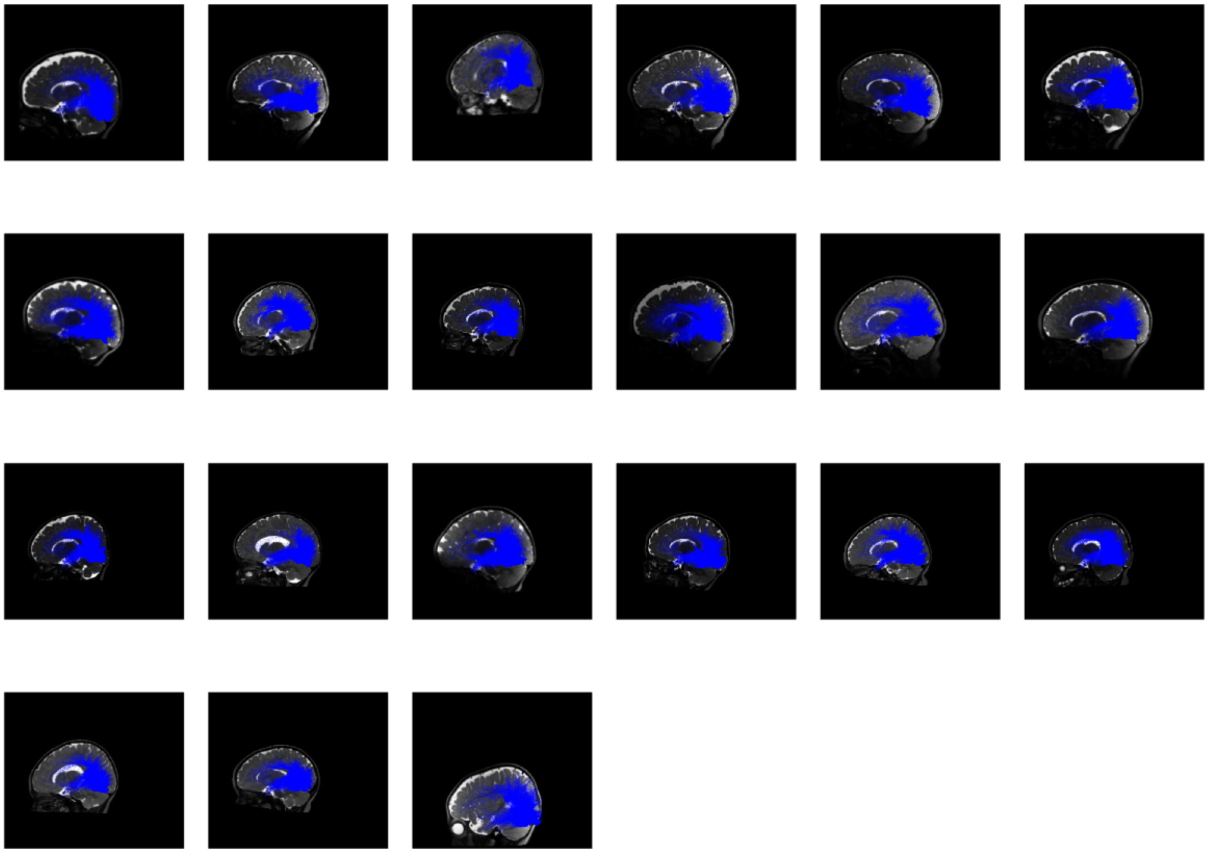

Supplementary Figure 53. White matter connections of pOTS-words in the 3-month age group organized by age in the left hemisphere.

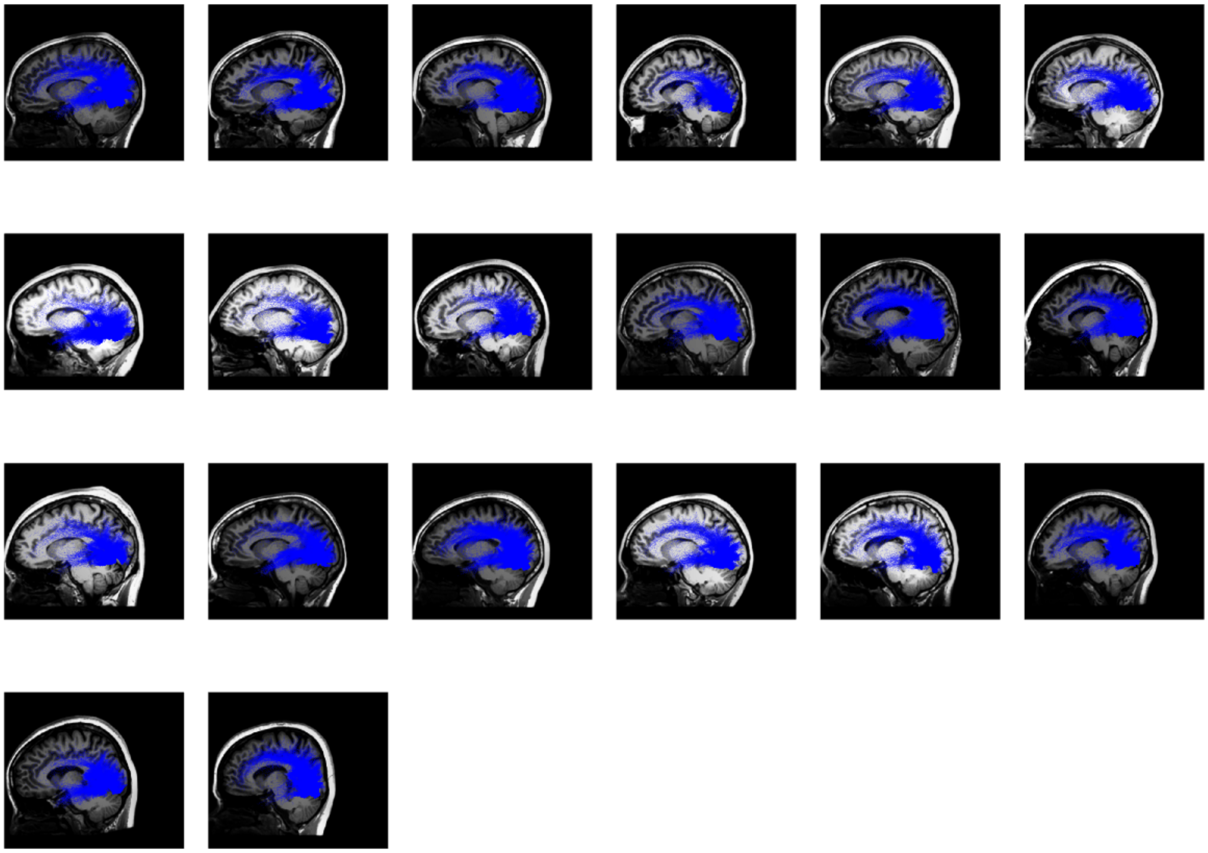

Supplementary Figure 54. White matter connections of pOTS-words in the adult age group organized by age in the left hemisphere.

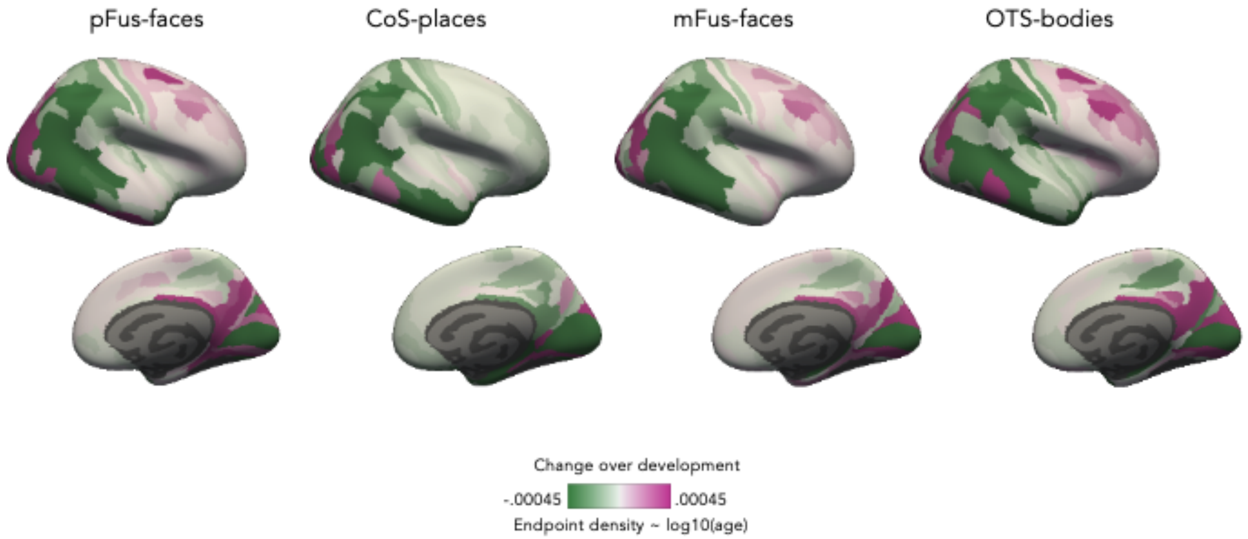

Supplementary Figure 55. Change in connectivity endpoint density over development within each Glasser Atlas ROI in the right hemisphere. For each fROI's connectivity profile and Glasser ROI, we fit a linear model predicting endpoint density by log10(age in days). The color of the Glasser ROI indicates the slope. *Green:* decreasing endpoint density over development; *magenta:* increasing endpoint density over development.

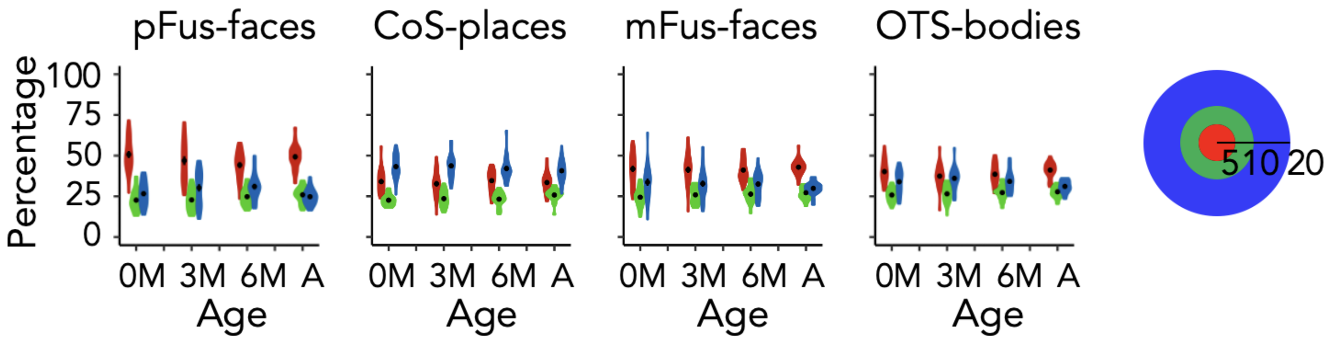

Supplementary Figure 56. White matter connections by early visual cortex eccentricity band in the right hemisphere. Quantification of the percentage of connections belonging to each eccentricity band across each age group. *0M:* newborns (n = 23), *3M:* 3 month-olds (n = 23), *6M:* 6 month-olds (n = 21), *A*: adults (n = 21). All panels: *red:* 0-5°, *green:* 5-10°, *blue:* 10-20°, see colorbar at right. Black dot and error bar indicate mean± standard error of the mean.

@mFus_bb05.mov

Supplementary Video 1. Video of white matter connections between mFus-faces and early visual cortex in an example newborn participant. Streamlines are colored by eccentricity band. *Red:* 0-5°, *green:* 5-10°, *blue:* 10-20°.

@CoS-bb05.mov

Supplementary Video 2. Video of white matter connections between CoS-places and early visual cortex in an example newborn participant. Streamlines are colored by eccentricity band. *Red:* 0-5°, *green:* 5-10°, *blue:* 10-20°.

@mFus-adult.mov

Supplementary Video 3. Video of white matter connections between mFus-faces and early visual cortex in an example adult. Streamlines are colored by eccentricity band. *Red:* 0-5°, *green:* 5-10°, *blue:* 10-20°.

@Cos-adult.mov

Supplementary Video 4. Video of white matter connections between CoS-places and early visual cortex in an example adult participant. Streamlines are colored by eccentricity band. *Red:* 0-5°, *green:* 5-10°, *blue:* 10-20°.

Supplementary Table 1. Linear models (lm in R https://www.rdocumentation.org/packages/stats/versions/3.6.2/topics/lm) predicting endpoint density by Glasser ROI and log10(age) for each fROI. P-values are Bonferroni corrected for multiple comparisons.

(a) Main effect of Glasser ROI:
Model comparison: endpoint density ~ log10(age in days)
vs endpoint density ~ Glasser_ROI + log10(age in days)

**fROI**	**df**	**Sum of Squares**	**F**	***p***
pFus-faces	168	0.22	91.52	<0.001
pOTS-words	168	1.91	454.02	<0.001
CoS-places	168	1.87	591.41	<0.001
mFus-faces	168	0.05	74.94	<0.001
OTS-bodies	168	0.08	81.61	<0.001
mOTS-words	168	1.04	210.14	<0.001

(b) Glasser ROI x Age interaction:
Model comparison: endpoint density ~ Glasser_ROI + log10(age in days)
vs endpoint density ~ GlasserROI X log10(age in days)

**fROI**	**df**	**Sum of Squares**	**F**	***p***
pFus-faces	168	0.01	3.97	<0.001
pOTS-words	168	0.03	6.36	<0.001
CoS-places	168	0.03	11.86	<0.001

mFus-faces	168	0.002	3.54	<0.001
OTS-bodies	168	0.005	4.96	<0.001
mOTS-words	168	0.017	3.63	<0.001

Supplementary Table 2. Linear models (lm in R https://www.rdocumentation.org/packages/stats/versions/3.6.2/topics/lm) predicting endpoint density by Glasser ROI and log10(corrected_age) for each fROI, where corrected age is the age in days minus the number of days born before 40 weeks of gestation. Two babies (four sessions) had missing gestational ages, and one baby had a negative corrected age, therefore, five sessions are excluded from these analyses. For adults, we do not have data for gestational age, and use age in days in the place of the corrected age. P-values are Bonferroni corrected for multiple comparisons.

(a) Main effect of Glasser ROI:
Model comparison: endpoint density ~ log10(corrected age in days)
vs endpoint density ~ Glasser_ROI + log10(corrected age in days)

**fROI**	**df**	**Sum of Squares**	**F**	***p***
pFus-faces	168	0.22	89.44	<0.001
pOTS-words	168	1.88	445.17	<0.001
CoS-places	168	1.86	584.80	<0.001
mFus-faces	168	0.05	74.15	<0.001
OTS-bodies	168	0.08	80.41	<0.001
mOTS-words	168	1.04	210.42	<0.001

(b) Glasser ROI x Age interaction:
Model comparison: endpoint density ~ Glasser_ROI + log10(corrected age in days)
vs endpoint density ~ GlasserROI X log10(corrected age in days)

**fROI**	**df**	**Sum of Squares**	**F**	***p****
pFus-faces	168	0.009	3.79	<0.001
pOTS-words	168	0.02	5.66	<0.001
CoS-places	168	0.03	11.30	<0.001
mFus-faces	168	0.002	3.45	<0.001
OTS-bodies	168	0.004	4.94	<0.001
mOTS-words	168	0.02	3.90	<0.001

Supplementary Table 3. Development of Endpoint Density for pFus-faces in the left hemisphere. Slopes and significance for linear models (lm in R https://www.rdocumentation.org/packages/stats/versions/3.6.2/topics/lm) for each Glasser ROI, relating pFus-faces endpoint density in each Glasser ROI to age. Model: endpoint density in Glasser ROI ~ log10(age in days). p-values are uncorrected for multiple comparisons.

Glasser ROI	Slope	p-value
1	-2.09E-07	0.99338142
2	-0.0002083	0.0574478
3a	-0.0001187	0.37294986
3b	-0.0002592	0.00230718
4	0.00014964	0.02324739
5L	-0.0001438	0.00097291

5m	-2.53E-05	0.01229257
5mv	-0.0001707	0.0002866
6a	0.00041474	5.41E-05
6d	6.69E-05	0.01341074
6ma	3.59E-05	0.06472006
6mp	-3.70E-05	0.35347198
6r	5.99E-05	0.05449107
6v	3.65E-05	0.00280483
7AL	-0.0002767	0.00170451
7Am	-0.000141	0.18281998
7m	0.00037707	0.00148766
7PC	-8.81E-05	0.05272821
7PL	0.00015659	0.05785285
7Pm	7.01E-05	0.28791273
8Ad	0.00021953	7.44E-08
8Av	0.00033762	4.42E-12
8BL	2.32E-05	0.00184995
8BM	1.93E-05	0.00885228
8C	0.00055047	1.31E-13
9-46d	0.00017071	8.20E-08
9a	3.20E-05	0.03919171
9m	2.13E-05	0.04544286
9p	2.79E-05	0.00264643
10d	9.08E-06	0.2987735
10pp	1.23E-05	0.24626557
10r	-7.55E-06	0.37098372
10v	1.31E-05	0.04177106
11l	7.84E-05	0.00200497
13l	-1.29E-05	0.71449167
23c	-7.35E-05	0.26284369
23d	-2.42E-05	0.16573759

24dd	-6.04E-06	0.81566722
24dv	3.75E-05	0.00750258
25	-1.51E-05	0.01761663
31a	5.72E-06	0.79393709
31pd	-5.05E-06	0.9548965
31pv	0.00013413	0.09042476
33pr	1.74E-06	0.53280805
43	2.28E-05	0.0004597
44	7.36E-05	1.13E-06
45	6.96E-05	1.69E-07
46	0.00017952	1.26E-09
47l	1.02E-05	0.00144494
47m	3.67E-06	0.45902841
47s	3.01E-06	0.51286675
52	-0.0003113	0.03136651
55b	0.00013095	6.22E-06
A1	-2.32E-05	0.03400918
A4	-6.62E-05	0.00536725
A5	-6.17E-05	0.00453509
a9-46v	4.40E-05	0.05142711
a10p	4.65E-05	3.92E-05
a24	-6.32E-06	0.29810705
a24pr	8.39E-06	0.21983492
a32pr	1.78E-05	0.01266449
a47r	7.73E-05	8.88E-05
AAIC	-4.15E-05	0.08836918
AIP	-0.0004529	0.00745212
AVI	7.83E-05	0.00310564
d23ab	0.00013037	0.0935627
d32	4.63E-06	0.56044889
DVT	-5.92E-05	0.83870568

EC	-4.82E-06	0.68549606
FEF	0.00015764	1.35E-05
FOP1	1.11E-05	0.00651079
FOP2	6.71E-05	0.0041442
FOP3	5.24E-05	0.0306128
FOP4	0.000147	0.00013654
FOP5	0.00014905	5.33E-08
FST	0.00027541	0.91724568
H	0.00034793	0.12740708
i6-8	0.00016286	3.67E-08
IFJa	0.00024407	7.60E-08
IFJp	0.0001866	0.00030513
IFSa	0.00014258	3.88E-09
IFSp	0.00010232	5.69E-08
Ig	4.71E-05	0.02334003
IP0	0.00097995	0.01005299
IP1	-0.0005362	0.05552553
IP2	-0.0008111	0.00052184
IPS1	-0.0002297	0.44863197
LBelt	-0.0001296	0.02191268
LIPd	-0.0008406	0.00133628
LIPv	-0.0001407	0.09063391
LO1	0.00148681	0.13240192
LO2	0.00165584	0.09334997
LO3	0.0010383	0.06957662
MBelt	-0.0001525	0.00065466
MI	-7.05E-05	0.01256127
MIP	-0.00011	0.64899889
MST	-0.0031381	0.01933475
MT	-1.73E-05	0.96516077
OFC	1.37E-05	0.39906078

OP1	1.19E-05	0.68570702
OP2-3	5.53E-05	0.21499906
OP4	1.69E-05	0.1093269
p9-46v	0.00021787	1.60E-10
p10p	2.02E-05	0.03443541
p24	2.10E-06	0.61407698
p24pr	2.37E-06	0.69589233
p32	-3.18E-06	0.64718462
p32pr	2.63E-05	0.01099533
p47r	6.22E-05	0.00181224
PBelt	-0.0001116	0.00428385
PCV	-0.0001933	0.00349511
PeEc	6.86E-05	0.13366908
PEF	4.50E-05	0.11153424
PF	-0.0001723	0.01844862
PFcm	-4.06E-05	0.39540053
PFm	-0.0008419	0.01053865
PFop	-4.97E-06	0.85196107
PFt	-0.0001814	0.02102498
PGi	-0.0017626	0.0011029
PGp	0.00189586	3.10E-05
PGs	-1.02E-05	0.97822421
PHA1	0.00067176	2.68E-15
PHT	-0.0012493	0.00120075
PI	-2.94E-05	0.66992671
Pir	-7.28E-05	0.01919062
pOFC	-2.33E-05	0.04942619
PoI1	-3.03E-05	0.52467075
PoI2	-9.86E-05	0.00066285
POS1	0.00052004	1.03E-08
POS2	0.00061776	0.00138497

PreS	0.00111406	8.67E-14
ProS	-0.000531	0.2354044
PSL	-9.92E-05	0.00509584
RI	-0.0001358	0.01150878
RSC	0.00074679	1.81E-10
s6-8	4.44E-05	3.71E-06
s32	-4.77E-06	0.30383799
SCEF	3.05E-05	0.10164402
SFL	2.24E-05	0.14006858
STGa	-4.23E-05	0.00505938
STSda	-0.0002265	0.00028429
STSdp	-0.0003712	0.00021977
STSva	-0.000255	0.09759018
STSvp	-0.0010191	0.00031424
STV	-1.17E-05	0.22466533
TA2	-4.87E-05	0.05520746
TE1a	-0.0001769	0.26337709
TE1m	-0.0002722	0.05336723
TE1p	-0.0005211	0.02362634
TE2a	0.00060681	0.02399751
TGd	-9.54E-05	0.54061283
TGv	2.91E-05	0.68183832
TPOJ1	-0.0002342	0.0269904
TPOJ2	-0.0010167	0.01236571
TPOJ3	-0.0007176	0.11670988
V1	-0.0024928	0.07417306
V2	-3.36E-05	0.94226373
V3	-0.000241	0.74401188
V3A	0.00031572	0.33617086
V3B	0.0008191	0.1850266
V3CD	0.00064895	0.276009

V4	-0.0084617	0.01415976
V4t	0.00063202	0.484999
V6	-1.20E-04	0.68656348
V6A	0.00014674	0.08970808
V7	0.00014331	0.16184257
v23ab	0.00031909	1.46E-11
VIP	-4.45E-05	0.59524904
VMV1	0.00104255	5.55E-08

Supplementary Table 4. Development of Endpoint Density for OTS-words in the left hemisphere. Slopes and significance for the models relating pOTS endpoint density in each Glasser ROI to age. Model: endpoint density in Glasser ROI ~ log10(age in days).

Glasser ROI	Slope	p-value
1	-2.65E-05	0.26761147
2	-0.0003624	0.00166429
3a	-0.0003147	0.02554091
3b	-0.0003762	2.64E-05
4	3.34E-05	0.56820547
5L	-0.0001797	0.00087161
5m	-3.10E-05	0.00382829
5mv	-0.0002391	3.56E-05
6a	0.00035784	0.00013971
6d	6.38E-05	0.02384247
6ma	4.35E-05	0.02854697
6mp	-7.46E-05	0.08882267
6r	4.36E-05	0.04202929
6v	4.34E-05	7.53E-05
7AL	-0.000356	0.00083796
7Am	-0.0002316	0.03331897
7m	0.00036462	0.00201761

7PC	-0.0001507	0.0028716
7PL	0.00016528	0.01583931
7Pm	4.14E-05	0.52473975
8Ad	0.00020286	6.58E-07
8Av	0.0002871	6.60E-14
8BL	2.34E-05	0.00054113
8BM	1.40E-05	0.04265927
8C	0.00043331	1.88E-14
9-46d	0.00013462	1.30E-06
9a	2.73E-05	0.01975729
9m	9.62E-06	0.26952225
9p	2.86E-05	0.00019573
10d	1.00E-05	0.13298772
10pp	1.46E-05	0.02598574
10r	-4.20E-06	0.56494537
10v	6.05E-06	0.23345281
11l	6.57E-05	0.00056967
13l	-2.60E-06	0.89231748
23c	-0.0001155	0.13323476
23d	-2.55E-05	0.12984337
24dd	8.71E-07	0.97636546
24dv	4.83E-05	0.00547808
25	-1.35E-05	0.02960392
31a	3.79E-06	0.86212625
31pd	-7.48E-05	0.43274233
31pv	0.00010308	0.17648061
33pr	1.72E-06	0.45492514
43	1.38E-05	0.01564612
44	3.85E-05	4.09E-05
45	5.91E-05	2.11E-06
46	0.0001744	4.94E-08

47l	7.74E-06	0.00214227
47m	6.77E-06	0.03311264
47s	2.97E-06	0.46911409
52	-0.0004083	0.00280468
55b	0.00011373	2.09E-06
A1	-2.90E-05	0.00661352
A4	-7.06E-05	0.00244598
A5	-5.35E-05	0.020791
a9-46v	4.84E-05	0.00175688
a10p	3.30E-05	0.00075119
a24	-1.84E-06	0.71751707
a24pr	1.31E-05	0.00078292
a32pr	9.70E-06	0.05589053
a47r	5.36E-05	1.76E-05
AAIC	-6.15E-05	0.00238964
AIP	-0.0006372	7.97E-05
AVI	5.27E-05	0.00257184
d23ab	7.80E-05	0.28430917
d32	4.54E-06	0.47295839
DVT	4.73E-05	0.85475735
EC	-4.50E-06	0.58301595
FEF	1.09E-04	6.77E-05
FOP1	9.01E-06	0.00537511
FOP2	4.43E-05	0.00407796
FOP3	5.09E-05	0.01254299
FOP4	0.00011622	1.90E-05
FOP5	0.00012289	8.80E-10
FST	-0.0019634	0.43234508
H	0.00018939	0.29156638
i6-8	0.00014761	3.41E-09
IFJa	0.00017054	4.29E-10

IFJp	0.00014506	0.00230991
IFSa	0.00014263	2.35E-07
IFSp	8.93E-05	4.34E-10
Ig	1.88E-05	0.22219836
IP0	0.00012852	0.69997448
IP1	-0.0008109	0.00213795
IP2	-0.0009613	1.24E-06
IPS1	-0.0004299	0.15782761
LBelt	-0.0001387	0.00901026
LIPd	-0.0010383	0.00011456
LIPv	-0.0001791	0.023753
LO1	-0.0015539	0.38739515
LO2	0.00386545	0.08763558
LO3	0.00018701	0.67065123
MBelt	-0.0001694	0.0001485
MI	-6.35E-05	0.00324279
MIP	-0.0002266	0.28455485
MST	-0.0052575	0.0072882
MT	-0.0012403	0.17383943
OFC	1.01E-05	0.39214674
OP1	-3.13E-05	0.23623648
OP2-3	-1.10E-05	0.71864328
OP4	2.86E-06	0.6814291
p9-46v	0.00022249	1.70E-12
p10p	1.50E-05	0.05403318
p24	3.24E-06	0.20842725
p24pr	6.18E-06	0.24312754
p32	-2.82E-06	0.58160945
p32pr	2.96E-05	0.0003801
p47r	5.43E-05	0.00013813
PBelt	-0.0001576	8.13E-05

PCV	-0.0003023	0.00034656
PeEc	-1.09E-05	0.74668023
PEF	1.83E-05	0.45566478
PF	-0.0002297	0.00100597
PFcm	-9.59E-05	0.01026343
PFm	-0.0009737	0.00014435
PFop	-3.36E-05	0.06747014
PFt	-0.0002437	0.00071361
PGi	-0.0025078	1.47E-06
PGp	0.00050469	0.16858995
PGs	-0.0008165	0.02346779
PHA1	0.0005485	3.02E-12
PHT	-0.0014131	0.0017162
PI	-8.14E-05	0.11967755
Pir	-9.55E-05	0.00023405
pOFC	-3.78E-05	0.00080481
PoI1	-4.72E-05	0.25255449
PoI2	-0.0001141	5.48E-06
POS1	0.00060619	2.45E-10
POS2	0.00044982	0.01262105
PreS	0.00088531	4.46E-15
ProS	-0.0004977	0.22819163
PSL	-0.0001564	8.84E-05
RI	-0.0002181	4.74E-05
RSC	0.00079201	9.74E-10
s6-8	3.78E-05	1.85E-06
s32	-5.88E-06	0.08401212
SCEF	1.77E-05	0.35687849
SFL	3.15E-05	0.02602392
STGa	-3.84E-05	0.00506822
STSda	-0.0001989	0.00172309

STSdp	-0.0004197	5.29E-06
STSva	-0.000296	0.01442929
STSvp	-0.0008698	0.00013761
STV	-2.26E-05	0.03755479
TA2	-4.77E-05	0.00195014
TE1a	-0.0002618	0.0639053
TE1m	-0.0002246	0.040491
TE1p	-0.0005502	0.09495084
TE2a	7.75E-05	0.67927798
TGd	-0.0002593	0.02679301
TGv	-5.61E-05	0.26543937
TPOJ1	-0.0003175	0.00490712
TPOJ2	-0.001097	0.00127906
TPOJ3	-0.0011158	0.00962074
V1	-0.002028	0.26222115
V2	-0.0009303	0.3353736
V3	-0.0042583	0.03963417
V3A	0.00088292	0.05060855
V3B	0.00045082	0.52042951
V3CD	0.00074277	0.12894642
V4	-0.014837	0.00024997
V4t	-0.0017957	0.35045646
V6	7.36E-05	0.8534415
V6A	0.00027238	0.00073752
V7	0.00021102	0.04994608
v23ab	0.00032375	1.76E-09
VIP	-9.10E-05	0.24159397
VMV1	0.00101473	3.82E-05

Supplementary Table 5. Development of Endpoint Density for CoS-places in the left hemisphere. Slopes and significance for linear models (lm in R https://www.rdocumentation.org/packages/stats/versions/3.6.2/topics/lm) for each

Glasser ROI, relating CoS-places endpoint density in each Glasser ROI to age. Model: endpoint density in Glasser ROI ~ log10(age in days). p-values are uncorrected for multiple comparisons.

Glasser ROI	slope	p-value
1	-2.75E-05	0.21519045
2	-0.000136	0.01127528
3a	-0.000101	0.04367822
3b	-0.0001444	0.00996932
4	-7.27E-05	0.07539221
5L	-4.22E-05	0.00163511
5m	-9.92E-06	0.01542328
5mv	-8.17E-05	0.00016011
6a	6.81E-05	0.01911534
6d	5.58E-06	0.60828319
6ma	-4.94E-06	0.48309606
6mp	-3.72E-05	0.00165086
6r	-9.22E-06	0.40323232
6v	5.96E-06	0.33595803
7AL	-0.000121	0.00186461
7Am	-8.60E-05	0.09309486
7m	5.70E-05	0.20295238
7PC	-2.99E-05	0.17920482
7PL	-6.24E-06	0.87176637
7Pm	-3.39E-05	0.38850457
8Ad	1.11E-06	0.96126002
8Av	6.07E-05	0.00550741
8BL	-1.39E-05	0.04759706
8BM	-2.29E-05	0.00160949
8C	9.36E-05	0.02077319
9-46d	-0.0001833	0.01157035
9a	-6.48E-05	0.04566399

9m	-5.92E-05	0.00857369
9p	-3.39E-05	0.02528392
10d	-7.88E-05	0.00064751
10pp	-0.0001095	0.00061652
10r	-9.16E-05	0.00105604
10v	-8.00E-05	0.00057029
11l	-0.0003008	4.77E-05
13l	-0.0003577	0.00012793
23c	-0.0001261	0.00118794
23d	-3.09E-05	0.05214842
24dd	-1.69E-05	0.04931556
24dv	-1.16E-05	0.25295472
25	-7.54E-05	0.00213719
31a	-2.31E-06	0.79686303
31pd	-0.0001062	0.10238728
31pv	-0.0002121	0.20643315
33pr	-6.81E-06	0.03792324
43	3.99E-06	0.13049542
44	-5.60E-06	0.39385133
45	-1.96E-05	0.02155223
46	-0.0001185	0.02287843
47l	-8.39E-06	0.12974232
47m	-3.73E-05	0.01417484
47s	-4.08E-05	0.01394026
52	-0.0001259	0.39970834
55b	-4.72E-06	0.7420379
A1	-2.27E-05	0.00768765
A4	-5.28E-05	0.04799626
A5	-2.75E-05	0.23454044
a9-46v	-0.0001554	0.00033418
a10p	-0.0001801	0.00010936

a24	-2.64E-05	0.02876316
a24pr	-4.22E-06	0.46310687
a32pr	-1.28E-05	0.0141394
a47r	-0.00013	0.00014568
AAIC	-1.00E-04	0.01000909
AIP	-0.0001911	0.00346255
AVI	-0.0001289	0.02118192
d23ab	-5.60E-05	0.32746306
d32	-1.89E-05	0.11868347
DVT	-0.0002508	0.30493163
EC	-0.0002918	5.11E-05
FEF	1.04E-05	0.51037577
FOP1	-4.61E-07	0.84057363
FOP2	2.90E-06	0.66380968
FOP3	-2.13E-06	0.80032299
FOP4	-4.45E-05	0.05960754
FOP5	-2.23E-05	0.13691087
FST	-0.0004553	0.63029913
H	-0.0058596	0.00177475
i6-8	3.46E-05	0.00330155
IFJa	2.16E-05	0.11353914
IFJp	5.90E-06	0.82730584
IFSa	-7.56E-05	0.01487092
IFSp	-1.71E-05	0.17883893
Ig	5.20E-06	0.41456732
IP0	0.00012932	0.45528767
IP1	-0.0001104	0.26373042
IP2	-0.0002541	0.00507329
IPS1	-0.0002351	0.03339326
LBelt	-8.68E-05	0.00152744
LIPd	-0.0002684	0.00825674

LIPv	-9.66E-05	0.03910791
LO1	-0.0003652	0.41071589
LO2	0.00087045	0.01180142
LO3	8.81E-05	0.74923298
MBelt	-0.0001256	0.00043786
MI	-0.0001018	0.00015153
MIP	-0.0001968	0.04880457
MST	-0.0011039	0.02128533
MT	-0.0003428	0.10647977
OFC	-0.0001923	0.00033047
OP1	-1.81E-05	0.2198457
OP2-3	2.08E-06	0.90628994
OP4	-8.73E-06	0.074588
p9-46v	-4.11E-05	0.14311801
p10p	-9.97E-05	0.00099175
p24	-4.68E-06	0.18836018
p24pr	-8.15E-06	0.12700169
p32	-5.38E-05	0.00295877
p32pr	-1.40E-05	0.0983749
p47r	-0.0001112	0.00167036
PBelt	-0.0001078	0.01038015
PCV	-8.18E-05	0.03948914
PeEc	-0.0002929	0.17913937
PEF	-1.14E-05	0.40213917
PF	-8.31E-05	0.08362275
PFcm	-4.25E-05	0.09543694
PFm	-0.0002547	0.02105968
PFop	-1.34E-05	0.10741174
PFt	-7.25E-05	0.01878706
PGi	-0.0007353	0.01052192
PGp	0.00040037	0.15251842

PGs	-1.26E-05	0.93481143
PHA1	0.00249768	0.00820388
PHT	-0.000361	0.27853945
PI	-6.66E-05	0.38511008
Pir	-0.0001184	0.01462204
pOFC	-0.0002206	0.00034507
PoI1	-7.73E-05	0.27541271
PoI2	-0.0001238	0.00216916
POS1	-8.66E-05	0.81263991
POS2	-0.0001073	0.37518384
PreS	-0.0052084	1.50E-05
ProS	3.50E-04	0.83476386
PSL	-6.20E-05	0.00305524
RI	-8.29E-05	0.07455268
RSC	-0.0009773	0.00060478
s6-8	1.95E-06	0.68377205
s32	-4.39E-05	0.00383964
SCEF	-2.27E-05	0.00796147
SFL	-2.23E-05	0.02068972
STGa	-6.71E-05	0.0080951
STSda	-0.0002431	0.0014616
STSdp	-0.0003377	0.00591163
STSva	-0.0004033	0.08758512
STSvp	-0.000776	0.00168482
STV	-1.74E-05	0.02906888
TA2	-7.16E-05	0.00921531
TE1a	-0.000503	0.13514632
TE1m	-0.0002613	0.30325695
TE1p	0.00075419	0.01838276
TE2a	0.00059829	0.2685112
TGd	-0.0003173	0.40646868

TGv	-0.0001262	0.66455138
TPOJ1	-0.0002435	0.00027368
TPOJ2	-0.0008556	0.00814426
TPOJ3	-0.00042	0.13173245
V1	-0.0060931	0.08345273
V2	0.00125124	0.33351673
V3	-0.0026854	0.05285109
V3A	0.00024869	0.13887691
V3B	-0.0003863	0.04890702
V3CD	-0.0002354	0.30825418
V4	-0.0041386	0.02435586
V4t	1.85E-05	0.95504518
V6	3.98E-05	0.80307863
V6A	0.00010519	0.00184301
V7	9.24E-05	0.01012315
v23ab	8.51E-06	0.89364107
VIP	-4.23E-05	0.40289246
VMV1	0.01662464	3.91E-12

Supplementary Table 6. Development of Endpoint Density for mFus-faces in the left hemisphere. Slopes and significance for linear models (lm in R https://www.rdocumentation.org/packages/stats/versions/3.6.2/topics/lm) for each Glasser ROI, relating mFus-faces endpoint density in each Glasser ROI to age. Model: endpoint density in Glasser ROI ~ log10(age in days). p-values are uncorrected for multiple comparisons.

Glasser ROI	Slope	p-value
1	-2.51E-05	0.54428889
2	-0.0002125	0.06088184
3a	-8.61E-05	0.49406147
3b	-0.0003283	0.00023081
4	9.44E-05	0.16647953
5L	-0.0001131	0.00091245

5m	-2.15E-05	0.00647186
5mv	-0.0001912	2.08E-05
6a	0.00029426	0.0004866
6d	3.62E-05	0.11851896
6ma	5.41E-06	0.74233159
6mp	-6.15E-05	0.00880254
6r	0.00012143	0.00025202
6v	8.52E-05	1.72E-05
7AL	-0.000217	0.04774239
7Am	-2.45E-05	0.8378981
7m	0.00057721	1.86E-05
7PC	-4.57E-05	0.39492267
7PL	0.0002835	0.00374137
7Pm	0.00013155	0.1069562
8Ad	0.00011268	0.00185402
8Av	0.00043927	1.21E-08
8BL	1.29E-05	0.16217596
8BM	-7.11E-06	0.50091822
8C	0.00079673	2.74E-10
9-46d	0.0001194	0.00216124
9a	-1.93E-06	0.93571014
9m	1.49E-05	0.36078677
9p	7.79E-06	0.53193903
10d	1.65E-05	0.26888011
10pp	6.02E-06	0.69634574
10r	-1.63E-05	0.23967191
10v	-8.59E-06	0.40235367
11l	7.57E-05	0.04501512
13l	-4.50E-06	0.88914576
23c	-0.0002595	0.00300653
23d	-6.45E-05	0.02107262

24dd	-2.89E-05	0.06991563
24dv	-6.73E-06	0.69697357
25	-1.29E-05	0.20389175
31a	-2.08E-05	0.49416554
31pd	0.0001568	0.16097452
31pv	0.00027742	0.00924252
33pr	-1.41E-06	0.74734143
43	5.03E-05	1.57E-05
44	0.00011227	4.14E-08
45	8.33E-05	7.46E-08
46	0.00023351	1.66E-07
47l	2.70E-05	0.00017676
47m	3.35E-06	0.60641206
47s	2.03E-05	0.06652223
52	-0.000137	0.39766563
55b	0.00014195	7.20E-06
A1	-1.11E-05	0.05296647
A4	-8.98E-05	0.17375687
A5	-8.36E-05	0.05130074
a9-46v	5.02E-05	0.06194484
a10p	2.12E-05	0.28276824
a24	-3.79E-06	0.68765354
a24pr	-3.66E-06	0.46784541
a32pr	3.92E-08	0.99507823
a47r	9.45E-05	0.00038108
AAIC	-5.57E-05	0.08932402
AIP	-0.0005363	0.00619157
AVI	0.00014827	0.00022609
d23ab	0.00030292	0.00201282
d32	-1.09E-05	0.2031556
DVT	-4.09E-05	0.81277685

EC	-7.78E-06	0.76173672
FEF	0.00020268	0.00011422
FOP1	2.52E-05	0.00144453
FOP2	0.00012255	1.16E-05
FOP3	0.00012649	8.84E-05
FOP4	0.00026068	1.55E-07
FOP5	0.00026459	8.38E-07
FST	0.00178219	0.16290276
H	-9.12E-05	0.82157075
i6-8	0.00018254	9.49E-09
IFJa	0.00035942	1.74E-11
IFJp	0.00028828	2.54E-06
IFSa	0.00020274	1.36E-08
IFSp	0.00018632	1.76E-07
Ig	8.11E-05	0.00239512
IP0	0.00047479	0.08313963
IP1	-8.11E-05	0.73797684
IP2	-0.000719	0.00045079
IPS1	-0.0002018	0.30779081
LBelt	-0.0001025	0.00671563
LIPd	-0.0006219	0.00450367
LIPv	-4.53E-05	0.61897237
LO1	0.00029145	0.46075332
LO2	0.00075523	0.00124903
LO3	0.0004818	0.28367489
MBelt	-0.0001236	0.00104081
MI	-7.48E-05	0.03893573
MIP	9.04E-05	0.69327315
MST	-0.0006774	0.23102651
MT	0.00027631	0.26656011
OFC	-1.96E-05	0.36674314

OP1	4.58E-05	0.23140088
OP2-3	0.00012314	0.02642348
OP4	2.21E-05	0.06186222
p9-46v	0.00030602	1.32E-07
p10p	-1.82E-07	0.9913342
p24	2.67E-06	0.55229697
p24pr	-1.87E-05	0.03802315
p32	-1.81E-05	0.09029329
p32pr	-1.38E-05	0.22236076
p47r	9.10E-05	0.00089205
PBelt	-0.0001305	0.13274833
PCV	-9.77E-05	0.25630982
PeEc	4.91E-05	0.70605738
PEF	5.63E-05	0.05650041
PF	-0.000189	0.05900935
PFcm	-8.34E-05	0.18970053
PFm	-0.0007072	0.01811461
PFop	-6.02E-06	0.82496099
PFt	-0.0001734	0.03223103
PGi	-0.0012346	0.02653926
PGp	0.00083994	0.05288936
PGs	0.00055468	0.15199313
PHA1	0.00057088	5.49E-06
PHT	-0.0015715	0.00491371
PI	-0.0001305	0.23639014
Pir	-9.60E-05	0.06828074
pOFC	-5.18E-05	0.00396892
PoI1	-3.60E-05	0.68069214
PoI2	-9.84E-05	0.01085657
POS1	0.00063512	3.49E-11
POS2	0.00095007	8.46E-05

PreS	0.00053586	0.00046359
ProS	-0.0005157	0.0882672
PSL	-0.0002115	0.00604394
RI	-0.0003503	0.00553181
RSC	0.00084313	3.80E-10
s6-8	2.09E-05	0.03173768
s32	-1.19E-05	0.09738145
SCEF	-2.64E-05	0.07490733
SFL	6.17E-06	0.65730012
STGa	-8.69E-05	0.00859461
STSda	-0.0002696	0.0021254
STSdp	-0.0003695	0.0496406
STSva	-0.0005315	0.09665279
STSvp	-0.0013162	0.0002906
STV	-3.43E-05	0.05544183
TA2	-0.0001268	0.00159838
TE1a	-0.0005883	0.03309857
TE1m	-0.0005811	0.03491273
TE1p	-0.0009084	0.10061759
TE2a	0.00044917	0.31684037
TGd	-0.0001702	0.53836094
TGv	1.19E-05	0.90923757
TPOJ1	-0.000474	0.00156419
TPOJ2	-0.0016643	0.00086792
TPOJ3	4.82E-05	0.9054877
V1	0.00019988	0.77586529
V2	0.00077056	0.00809789
V3	0.00064478	0.06296363
V3A	-5.43E-05	0.67989568
V3B	-5.84E-05	0.84914685
V3CD	-1.77E-05	0.95083746

V4	-0.0009192	0.27854304
V4t	0.00025705	0.43723411
V6	-0.0001569	0.17955203
V6A	2.24E-05	0.68598632
V7	1.97E-05	0.71477063
v23ab	0.00032347	3.87E-11
VIP	0.00014078	0.15751247
VMV1	0.00051827	0.00080369

Supplementary Table 7. Development of Endpoint Density for OTS-bodies in the left hemisphere. Slopes and significance for linear models (lm in R https://www.rdocumentation.org/packages/stats/versions/3.6.2/topics/lm) for each Glasser ROI, relating OTS-bodies endpoint density in each Glasser ROI to age. Model: endpoint density ~ log10(age in days). p-values are uncorrected for multiple comparisons.

Glasser ROI	Slope	p-value
1	-6.45E-05	0.13908334
2	-0.0005186	0.00153575
3a	-0.0002293	0.11628034
3b	-0.0005556	1.05E-05
4	6.70E-05	0.3548793
5L	-0.0001977	0.0001712
5m	-4.26E-05	0.00018571
5mv	-0.000323	9.14E-08
6a	0.00026739	0.00425353
6d	2.63E-05	0.37072785
6ma	-8.39E-06	0.61035814
6mp	-0.0001112	0.00032089
6r	0.00015627	1.26E-04
6v	0.00010114	6.41E-07
7AL	-0.0003708	0.00724287
7Am	-0.0001631	0.21585673
7m	0.00069435	9.55E-08

7PC	-0.0002135	0.01692127
7PL	0.00028369	0.00137163
7Pm	0.00019526	0.0136224
8Ad	0.00013811	1.91E-04
8Av	0.00055751	1.04E-08
8BL	7.16E-06	0.4147813
8BM	-9.86E-06	0.33224966
8C	0.00095166	8.91E-11
9-46d	8.79E-05	0.06611318
9a	3.23E-06	0.89052381
9m	1.75E-06	0.9183377
9p	8.52E-06	0.51186993
10d	3.92E-06	0.7949581
10pp	5.02E-06	0.78021381
10r	-3.12E-05	0.03914266
10v	-8.24E-06	0.48543045
11l	6.85E-05	0.10256702
13l	2.78E-05	0.41713342
23c	-0.0004138	2.26E-05
23d	-8.77E-05	0.00855996
24dd	-7.28E-05	0.00073487
24dv	-3.71E-05	0.08942162
25	-2.08E-05	0.06389866
31a	-2.67E-05	0.46956713
31pd	0.00015295	0.16486499
31pv	0.00028666	0.0122369
33pr	-3.86E-06	0.37615885
43	5.18E-05	1.94E-05
44	0.00013328	1.62E-11
45	0.00014347	1.28E-07
46	0.00024569	3.15E-06

47l	3.39E-05	0.00043306
47m	4.51E-06	0.43978416
47s	2.72E-05	0.00240921
52	-1.09E-04	0.38670621
55b	0.00018945	3.50E-06
A1	-2.80E-05	0.20730787
A4	-9.77E-05	0.07299988
A5	-6.51E-05	0.06040978
a9-46v	5.64E-05	0.05571752
a10p	2.15E-05	0.40023281
a24	-1.21E-05	0.30179695
a24pr	2.55E-06	0.66127171
a32pr	2.07E-06	0.72411644
a47r	0.00010961	0.00029989
AAIC	-3.07E-05	0.3915287
AIP	-0.0009152	9.52E-05
AVI	0.00024376	2.70E-06
d23ab	0.00030602	0.00185482
d32	-3.98E-06	0.71986763
DVT	-3.25E-05	0.83559168
EC	-4.82E-05	0.34195887
FEF	0.00024477	0.00028226
FOP1	3.17E-05	9.00E-06
FOP2	0.00018242	1.75E-07
FOP3	0.00022244	4.05E-08
FOP4	0.0003873	1.12E-10
FOP5	0.00039092	4.33E-07
FST	0.00302444	0.10937567
H	-0.0005297	0.36587235
i6-8	0.00019959	3.68E-09
IFJa	0.00049759	5.96E-11

IFJp	0.00040629	9.23E-07
IFSa	0.00027011	5.85E-08
IFSp	0.0002503	2.56E-09
Ig	0.00010056	5.67E-05
IP0	0.0001022	0.6644022
IP1	-8.43E-05	0.73800056
IP2	-0.0009646	7.41E-05
IPS1	-0.0001968	0.23736549
LBelt	-0.0001863	0.02954404
LIPd	-0.0008614	0.0008802
LIPv	-0.0001346	0.12880634
LO1	-6.81E-05	0.83966859
LO2	0.00046957	0.01182697
LO3	7.09E-05	0.84761546
MBelt	-0.0001257	0.00017307
MI	-2.37E-05	0.52569393
MIP	7.47E-05	0.7239528
MST	-0.0007891	0.28144286
MT	0.00025061	0.27024273
OFC	-1.53E-05	0.52670671
OP1	4.87E-05	0.3003206
OP2-3	0.00019484	0.0050414
OP4	2.93E-05	0.03774062
p9-46v	0.00035519	8.27E-09
p10p	-8.70E-06	0.67353565
p24	7.06E-06	0.11405032
p24pr	-2.51E-05	0.0347772
p32	-2.62E-05	0.06636492
p32pr	-1.66E-05	0.18475925
p47r	0.00010613	0.00279726
PBelt	-0.0001447	0.04578319

PCV	-0.000213	0.03642791
PeEc	9.20E-05	0.5726744
PEF	7.84E-05	0.02612348
PF	-0.0002645	0.01664226
PFcm	-0.0001068	0.15742922
PFm	-0.0008683	0.01447437
PFop	-4.96E-05	0.08827253
PFt	-0.0003199	0.00064802
PGi	-0.0012648	0.03975206
PGp	0.00058632	0.14890361
PGs	0.00062699	0.10622648
PHA1	0.00063637	0.00014716
PHT	-0.0029084	0.00278043
PI	-0.0001181	0.29385347
Pir	-9.08E-05	0.0926628
pOFC	-7.07E-05	0.01185913
PoI1	2.36E-05	0.80369685
PoI2	-7.15E-05	0.0508298
POS1	0.00077283	6.59E-15
POS2	0.00096983	5.20E-05
PreS	0.00042058	0.00324843
ProS	-0.0004021	0.16782986
PSL	-0.000394	0.0012306
RI	-0.0006529	0.00091456
RSC	0.00096836	6.47E-12
s6-8	2.32E-05	0.02492618
s32	-1.88E-05	0.02304909
SCEF	-3.77E-05	0.02914942
SFL	1.37E-06	0.9211945
STGa	-7.54E-05	0.00814143
STSda	-0.0002486	0.00147378

STSdp	-0.0004354	0.01087942
STSva	-0.0005172	0.04234007
STSvp	-0.0018401	0.00031428
STV	-7.79E-05	0.00275333
TA2	-9.06E-05	0.0040287
TE1a	-0.0007029	0.01006699
TE1m	-0.0011452	0.00061256
TE1p	-0.0027556	0.00290927
TE2a	0.0002624	0.68622923
TGd	-0.0002061	0.4634956
TGv	7.54E-05	0.44870677
TPOJ1	-0.0009931	0.00122355
TPOJ2	-0.0022402	6.84E-06
TPOJ3	-7.45E-05	0.86735994
V1	0.00064	0.29974788
V2	0.00097076	0.00075568
V3	0.00079367	0.01238726
V3A	1.86E-06	0.98738153
V3B	-0.0002508	0.41118423
V3CD	-1.03E-04	0.68743816
V4	-0.000657	0.41135711
V4t	1.05E-05	0.97675397
V6	-0.0001376	0.1707629
V6A	3.61E-05	0.42231442
V7	2.27E-05	0.5627882
v23ab	0.00042686	2.34E-15
VIP	3.13E-05	0.75061537
VMV1	0.00062193	2.24E-07

Supplementary Table 8. Development of Endpoint Density for mOTS-words in the left hemisphere. Slopes and significance for linear models (lm in R https://www.rdocumentation.org/packages/stats/versions/3.6.2/topics/lm) for each

Glasser ROI, relating mOTS-words endpoint density in each Glasser ROI to age. Model: endpoint density in Glasser ROI ~ log10(age in days). p-values are uncorrected for multiple comparisons.

Glasser ROI	Slope	p-value
1	-5.01E-05	0.31912944
2	-0.0007516	0.0001441
3a	-0.0003346	0.06030991
3b	-0.0007335	7.51E-07
4	6.16E-05	0.41928078
5L	-0.0002676	4.57E-05
5m	-5.84E-05	5.04E-06
5mv	-0.0004747	8.84E-08
6a	0.00029228	0.00803742
6d	2.64E-05	0.45323604
6ma	-2.51E-05	0.22494564
6mp	-0.0001871	0.00048509
6r	0.00023364	5.69E-06
6v	0.00015609	6.64E-13
7AL	-0.0004914	0.00154699
7Am	-0.0001385	0.31166373
7m	0.00090514	1.76E-10
7PC	-0.0002589	0.0053204
7PL	0.00035506	0.00031738
7Pm	0.00027577	0.00586826
8Ad	0.00023046	1.83E-05
8Av	0.00069378	7.33E-14
8BL	1.36E-05	0.31579802
8BM	-7.53E-06	0.59344588
8C	0.00123852	6.02E-18
9-46d	0.0001484	0.00453179
9a	1.23E-05	0.70010818

9m	-9.94E-06	0.68756939
9p	1.60E-05	0.35910664
10d	-2.51E-06	0.8951469
10pp	-5.94E-06	0.76027502
10r	-4.12E-05	0.01079641
10v	7.54E-06	0.56695419
11l	0.00013797	0.00014973
13l	8.33E-05	0.01190187
23c	-0.0006162	1.15E-05
23d	-0.0001187	0.01239856
24dd	-0.0001104	0.00014255
24dv	-4.30E-05	0.08216882
25	-2.42E-05	0.04768959
31a	-1.45E-05	0.7897534
31pd	0.00018683	0.21124315
31pv	0.00038137	0.00997513
33pr	-6.34E-06	0.27771936
43	8.54E-05	1.10E-08
44	0.0001873	6.77E-17
45	0.0001852	6.79E-18
46	0.00041603	3.90E-07
47l	4.29E-05	1.11E-07
47m	1.22E-05	0.01121792
47s	4.42E-05	4.94E-06
52	-0.0003054	0.04054037
55b	0.00029051	4.70E-08
A1	-7.58E-05	0.00316852
A4	-0.0001228	0.00920997
A5	-7.73E-05	0.00741113
a9-46v	1.03E-04	0.00058731
a10p	5.14E-05	0.05607663

a24	-1.36E-05	0.25652902
a24pr	2.41E-07	0.9707224
a32pr	6.98E-06	0.42963526
a47r	0.00016315	8.23E-10
AAIC	-3.64E-05	0.32554489
AIP	-0.0012427	4.21E-06
AVI	0.00031934	4.68E-12
d23ab	0.00045306	0.00074712
d32	1.69E-06	0.90817059
DVT	-6.09E-05	0.71898668
EC	-3.30E-05	0.22277025
FEF	0.00030531	1.61E-06
FOP1	4.26E-05	7.15E-08
FOP2	0.00023889	1.66E-12
FOP3	0.00032133	1.51E-13
FOP4	0.00057743	2.70E-18
FOP5	0.00044838	4.75E-23
FST	0.00189127	0.57485433
H	-0.0003914	0.27244508
i6-8	0.00026262	8.97E-10
IFJa	0.00057206	1.61E-21
IFJp	0.000601	1.97E-11
IFSa	0.00035512	1.56E-15
IFSp	0.00031032	1.88E-14
Ig	0.00014596	1.79E-05
IP0	-0.0002373	0.33727763
IP1	-0.0002231	0.42205831
IP2	-0.0010883	9.59E-05
IPS1	-0.0002761	0.11977922
LBelt	-0.0003114	4.66E-05
LIPd	-0.0010225	0.00011417

LIPv	-0.0002261	0.01262674
LO1	-0.000105	0.73532971
LO2	0.00015886	0.43236792
LO3	-0.0001619	0.71111038
MBelt	-0.0001761	1.65E-05
MI	-2.95E-05	0.4795723
MIP	-0.0001198	0.59698392
MST	-0.0019053	0.10560026
MT	-5.00E-05	0.80321014
OFC	4.67E-06	0.86084669
OP1	9.02E-05	0.06950491
OP2-3	0.00033956	4.54E-06
OP4	3.78E-05	0.01889582
p9-46v	0.00045705	4.93E-13
p10p	1.05E-05	0.65141707
p24	7.94E-06	0.09363854
p24pr	-2.92E-05	0.02850694
p32	-3.18E-05	0.04306651
p32pr	-2.20E-05	0.09234664
p47r	0.00020467	1.08E-09
PBelt	-0.000233	0.00250405
PCV	-0.0002873	0.03190017
PeEc	0.00010817	0.25730594
PEF	0.00015257	0.00127944
PF	-0.0001612	0.2633617
PFcm	-0.000124	0.13586392
PFm	-0.0005592	0.16728617
PFop	-5.30E-05	0.0741977
PFt	-0.0003819	6.85E-05
PGi	-0.0012543	0.05507022
PGp	0.00030764	0.41760601

PGs	0.0004068	0.47628724
PHA1	0.00064485	1.57E-08
PHT	-0.0043903	0.18618563
PI	-0.0001829	0.10136606
Pir	-0.0001286	0.01235508
pOFC	-5.37E-05	0.04817059
PoI1	-0.0001167	0.2773499
PoI2	-0.0001454	0.00361702
POS1	0.00092764	7.19E-14
POS2	0.00114263	3.56E-06
PreS	0.00049437	7.00E-05
ProS	6.31E-05	0.84518499
PSL	-0.0004863	0.0006279
RI	-0.0010146	0.00045891
RSC	0.00150717	1.44E-13
s6-8	2.47E-05	0.05265723
s32	-2.59E-05	0.00215959
SCEF	-5.35E-05	0.03498789
SFL	-1.57E-05	0.40431034
STGa	-7.71E-05	0.00230504
STSda	-0.0003044	0.00018277
STSdp	-0.0010247	0.00024895
STSva	-0.0004946	0.03753667
STSvp	-0.0037341	0.00702297
STV	-8.37E-05	0.00903208
TA2	-8.09E-05	0.00225101
TE1a	-0.0004248	0.02860474
TE1m	-0.0008979	0.01428361
TE1p	-0.011576	0.02754983
TE2a	0.00196591	0.04190409
TGd	-0.0003496	0.14150582

TGv	-1.99E-05	0.8368654
TPOJ1	-0.0016987	0.00071698
TPOJ2	-0.0033462	0.01534868
TPOJ3	-1.78E-05	0.96837091
V1	-0.0001104	0.86591573
V2	0.00053705	0.06228073
V3	0.0003475	0.22630971
V3A	2.81E-05	0.8130781
V3B	-0.0003955	0.12656164
V3CD	-1.35E-05	0.94319501
V4	-0.0013047	0.19220565
V4t	-0.0001216	0.70914826
V6	-8.78E-05	0.36303332
V6A	8.63E-05	0.03703133
V7	1.37E-05	0.68208929
v23ab	0.0006183	1.23E-13
VIP	2.93E-05	0.76466769
VMV1	0.00070459	3.53E-05
